# Supplementary figures and images for: Decoding the biogenesis of HIV-induced CPSF6 puncta and their fusion with nuclear speckles (part 1 of 2)
Source: eLife. 2026 Jan 6;13:RP103725. doi: 10.7554/eLife.103725 (PMC12774418; doi:10.7554/eLife.103725)

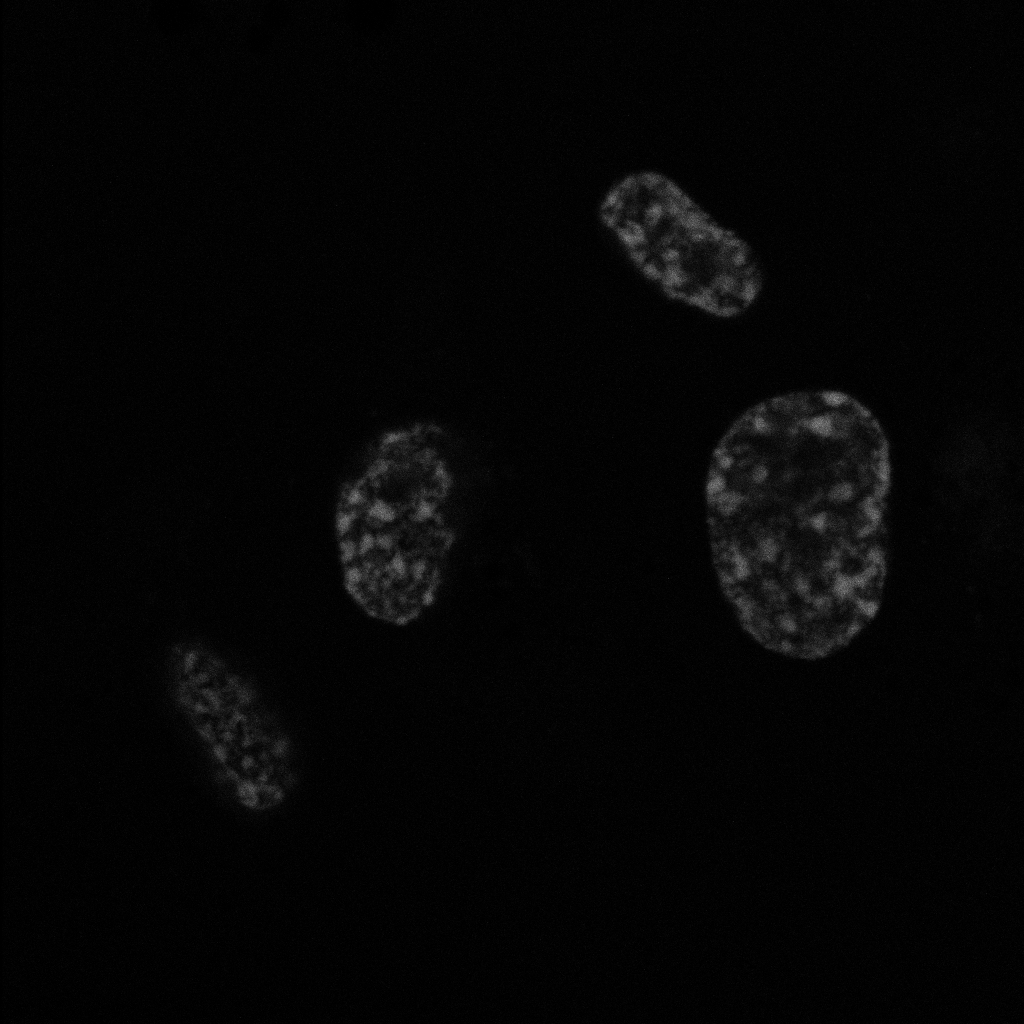

Supplement: Figure 1—source data 2. [file elife-103725-fig1-data2.zip › Figure 1-source data 2/Figure 1A/HIV + Nevirapine.tif]

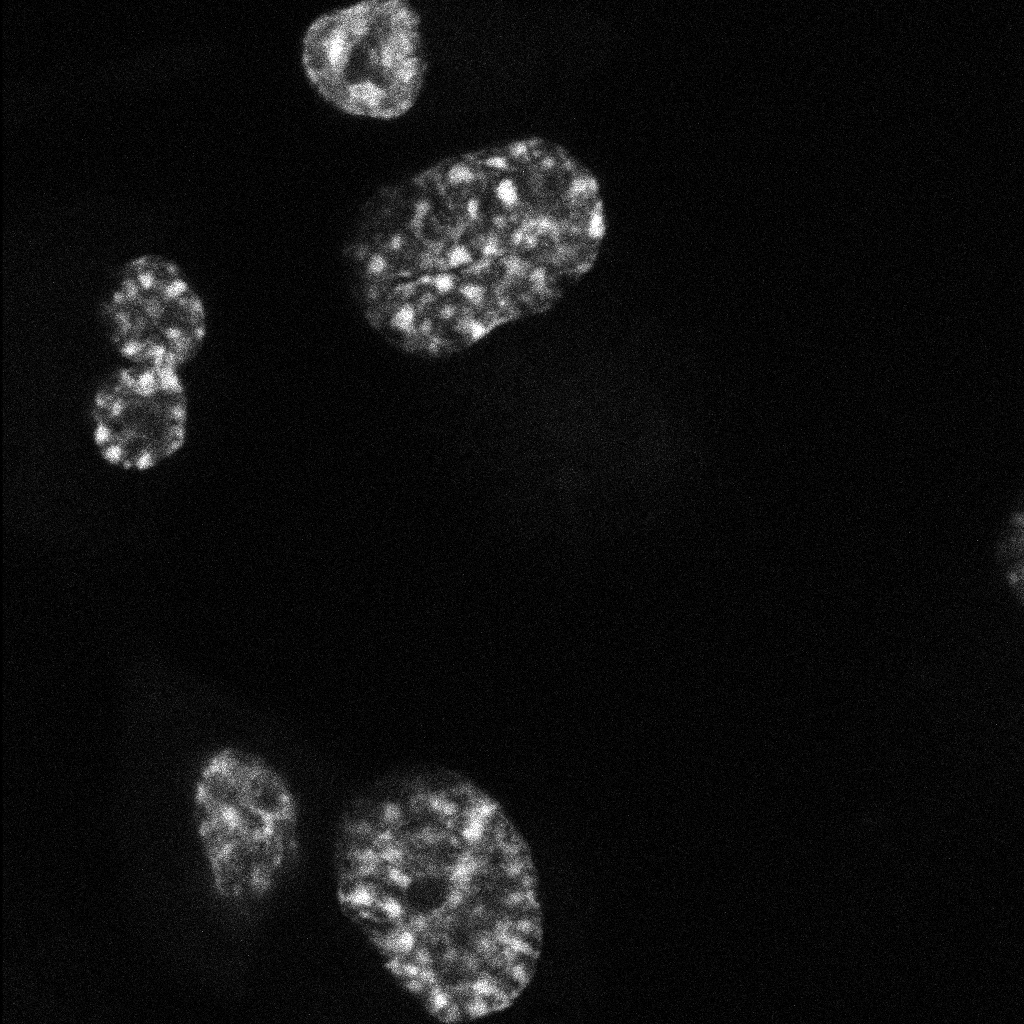

Supplement: Figure 1—source data 2. [file elife-103725-fig1-data2.zip › Figure 1-source data 2/Figure 1A/HIV+Nevirapine+Wash+PF74 25uM.tif]

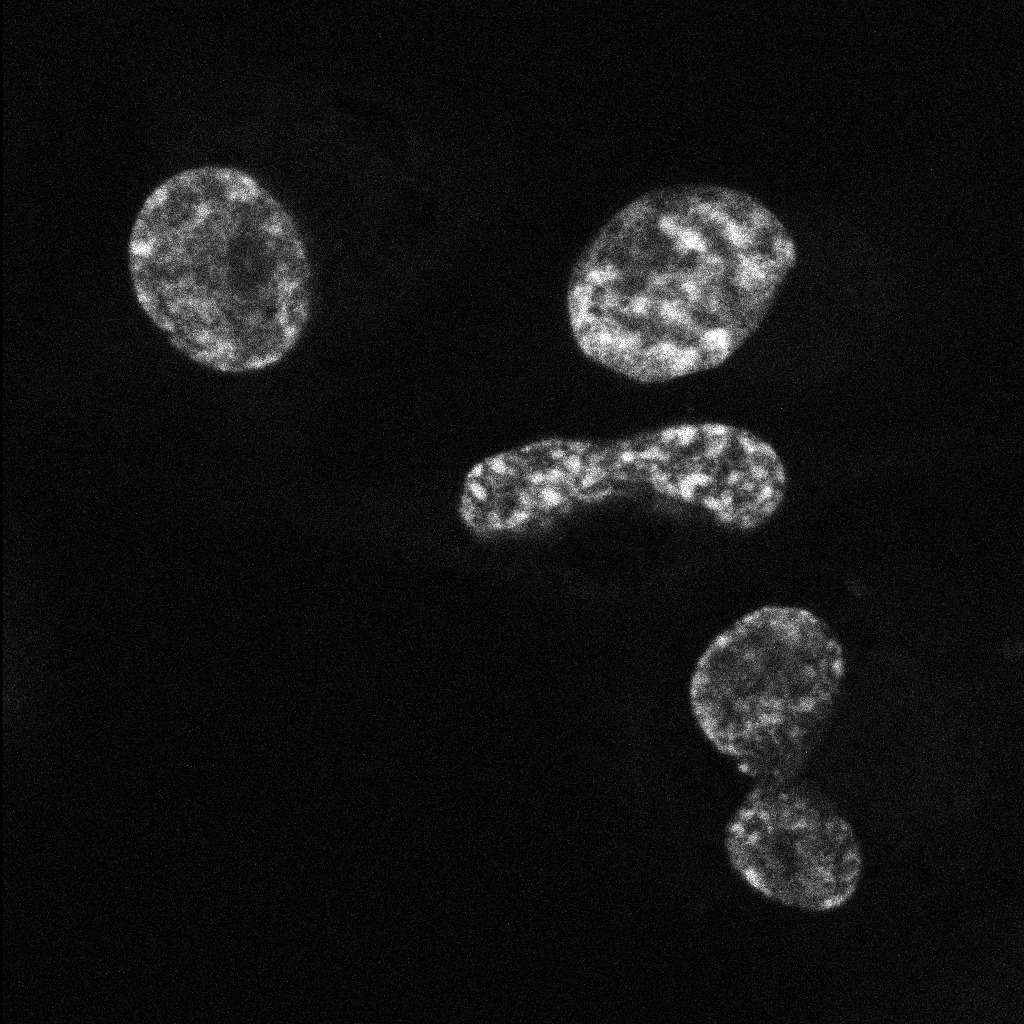

Supplement: Figure 1—source data 2. [file elife-103725-fig1-data2.zip › Figure 1-source data 2/Figure 1A/HIV+Nevirapine+wash.tif]

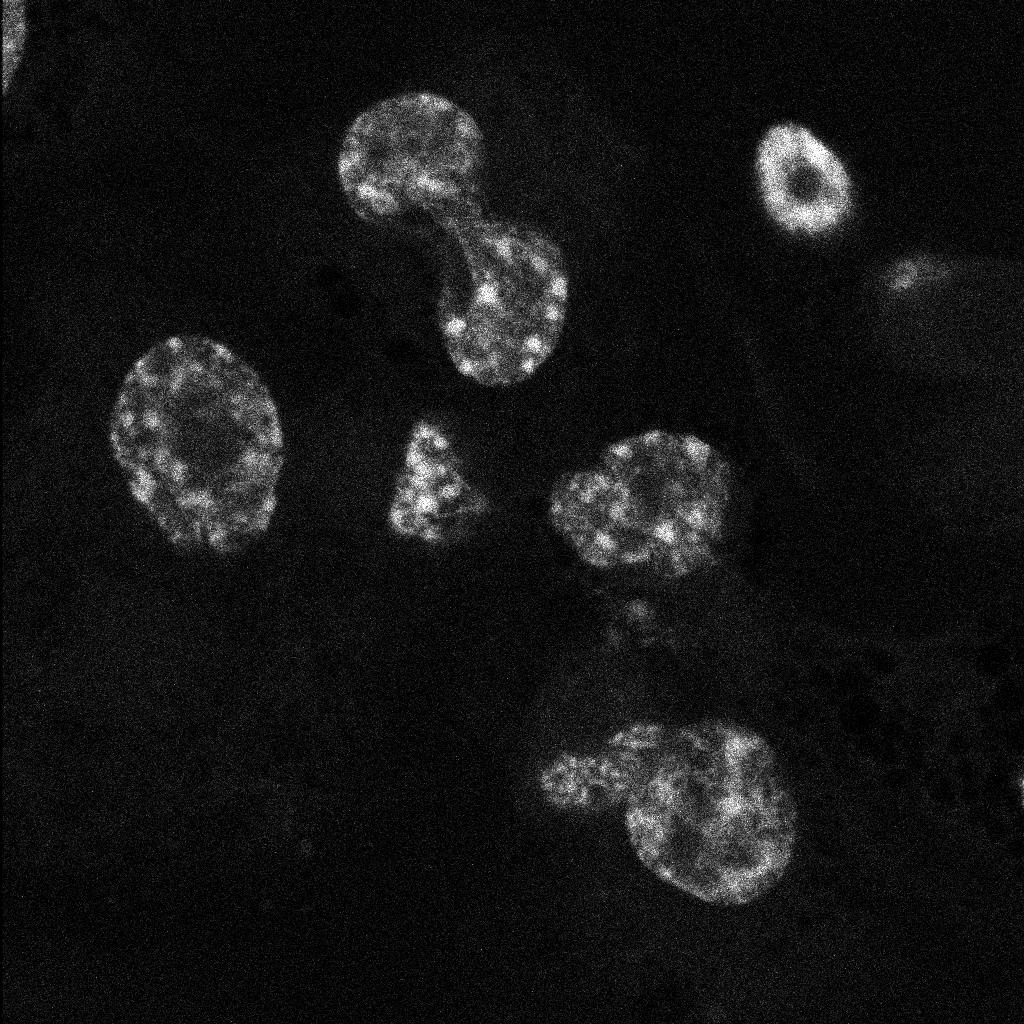

Supplement: Figure 1—source data 2. [file elife-103725-fig1-data2.zip › Figure 1-source data 2/Figure 1A/HIV.tif]

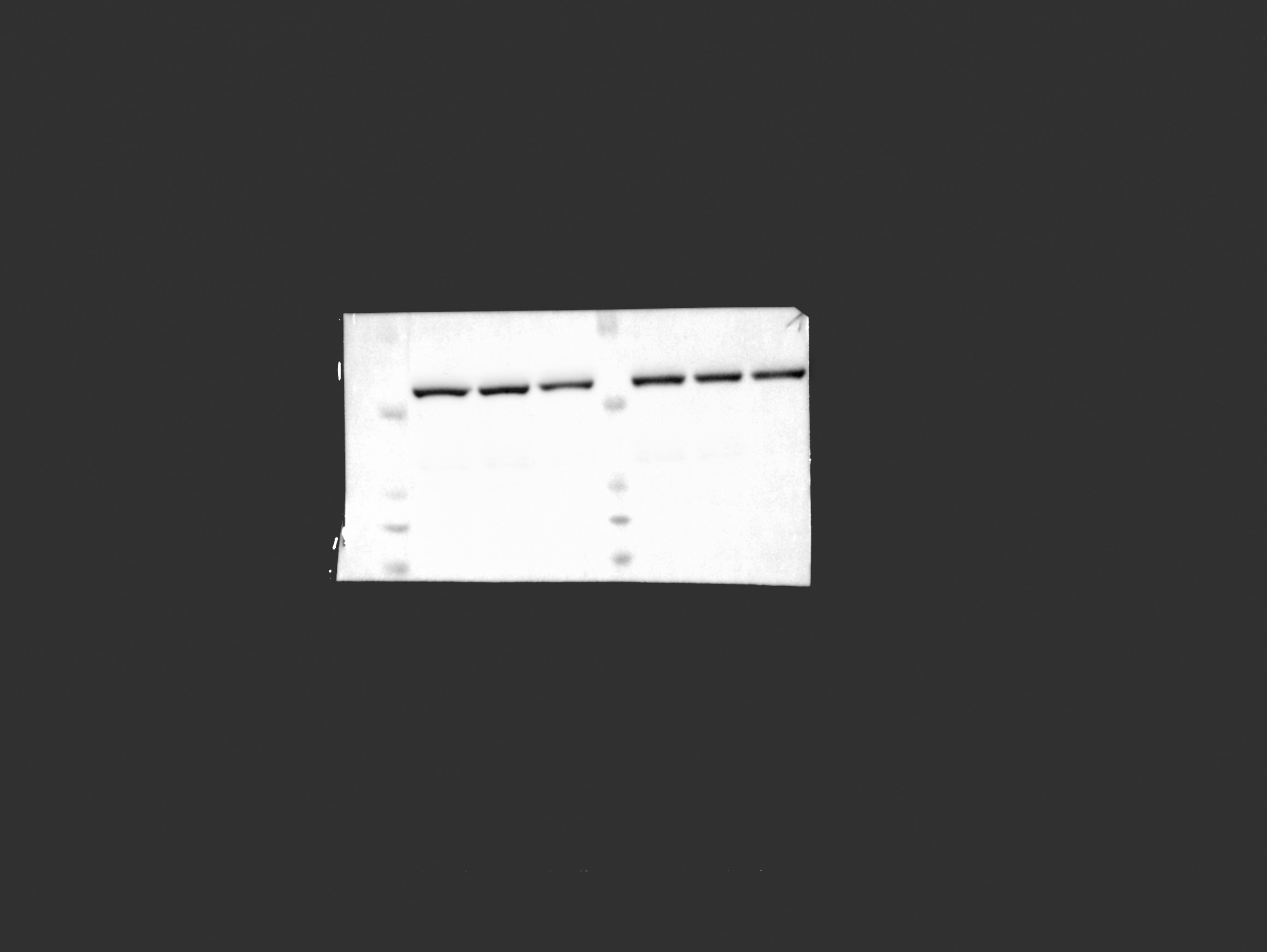

Supplement: Figure 1—source data 3. [file elife-103725-fig1-data3.zip › Figure 1-source data 3/Figure 1C/20230630 Actin.jpg]

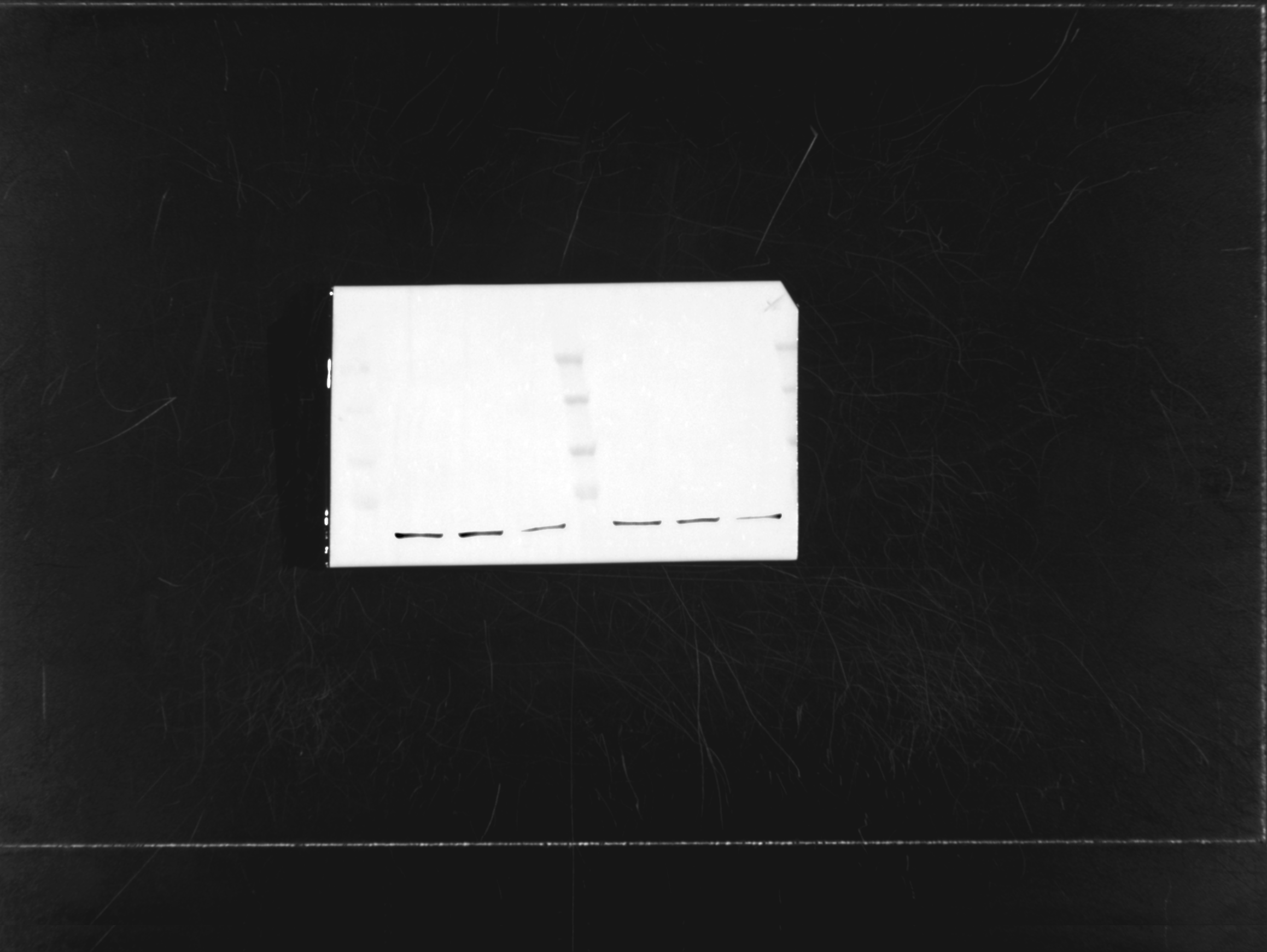

Supplement: Figure 1—source data 3. [file elife-103725-fig1-data3.zip › Figure 1-source data 3/Figure 1C/20230630 CPSF6.tif]

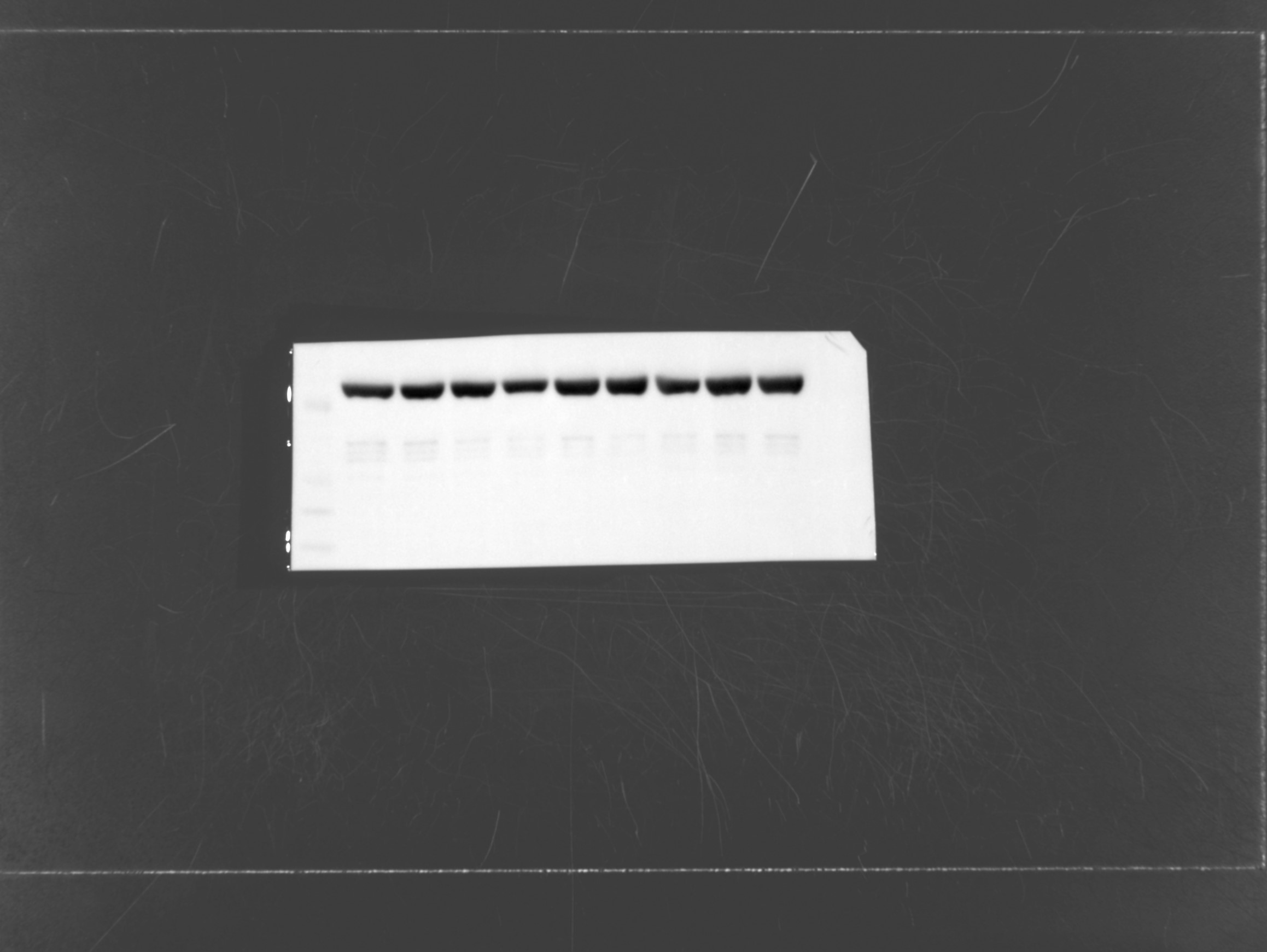

Supplement: Figure 1—source data 3. [file elife-103725-fig1-data3.zip › Figure 1-source data 3/Figure 1C/20230711 Actin.jpg]

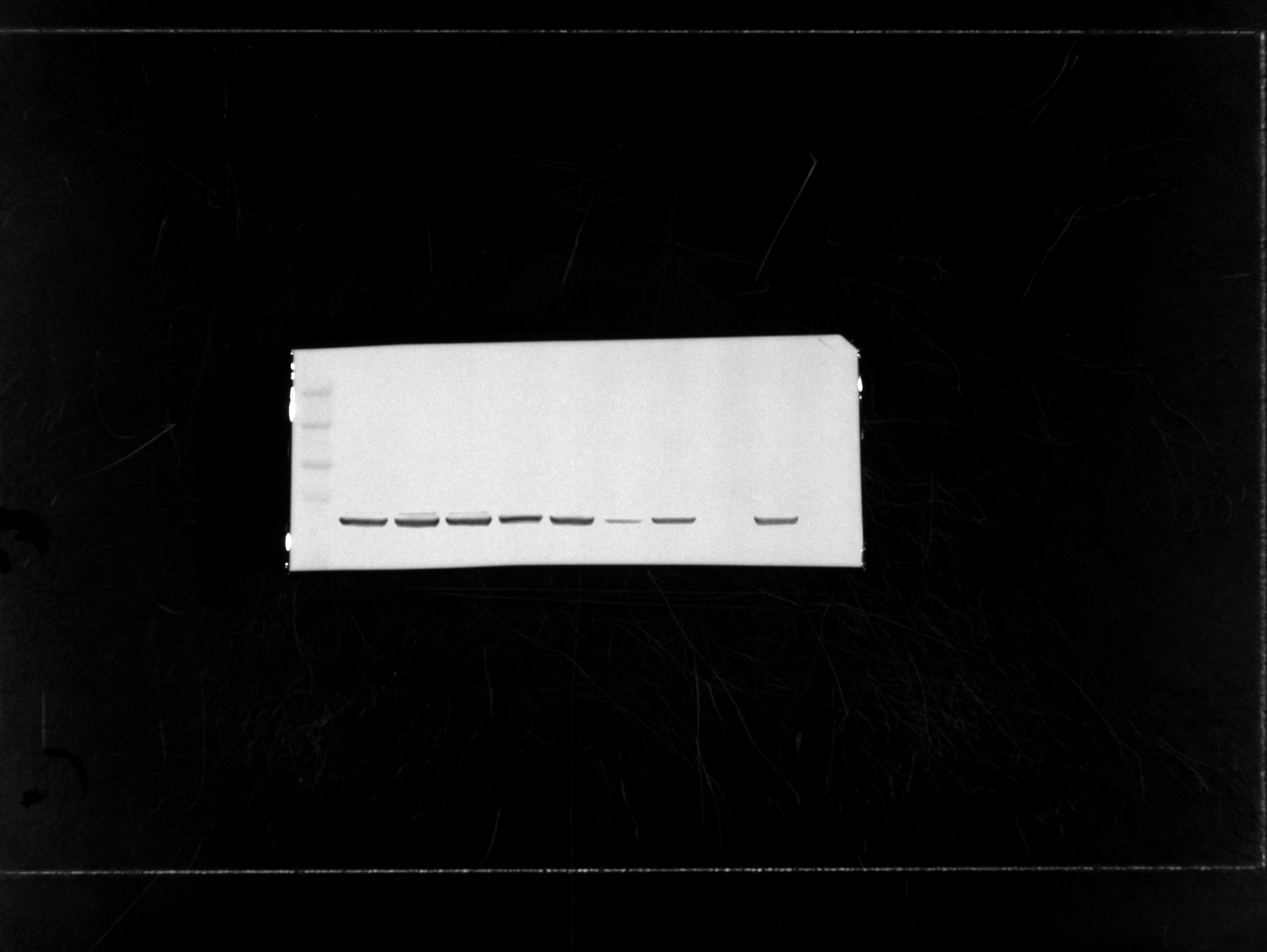

Supplement: Figure 1—source data 3. [file elife-103725-fig1-data3.zip › Figure 1-source data 3/Figure 1C/20230711 CPSF6.jpg]

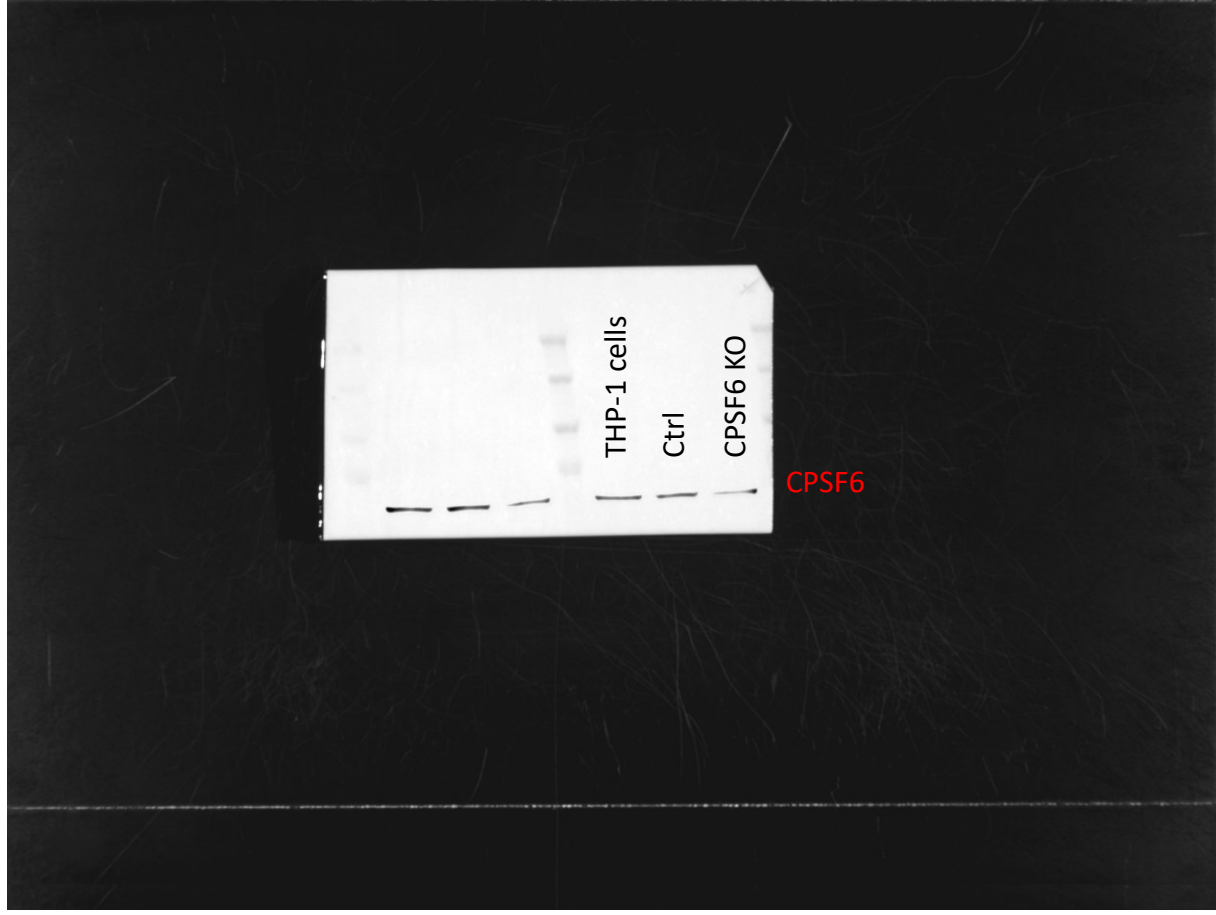

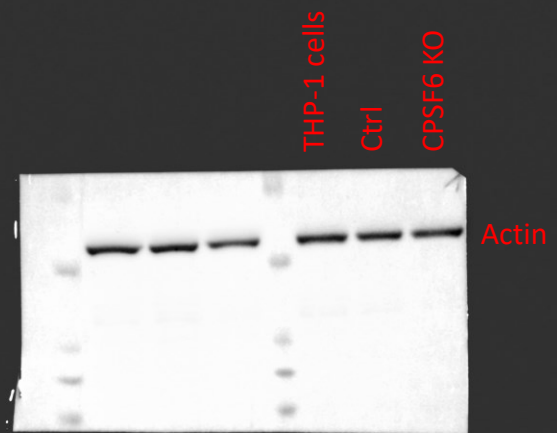

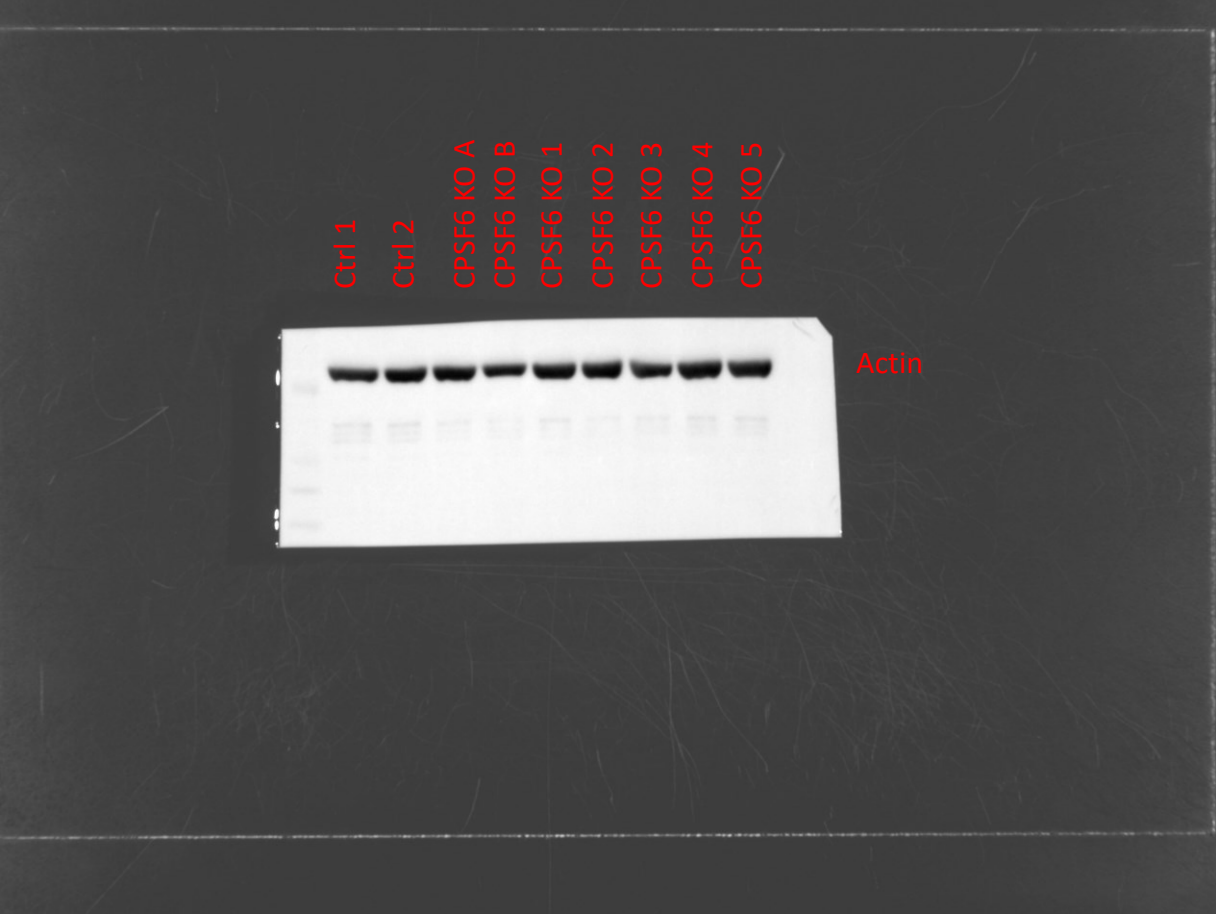

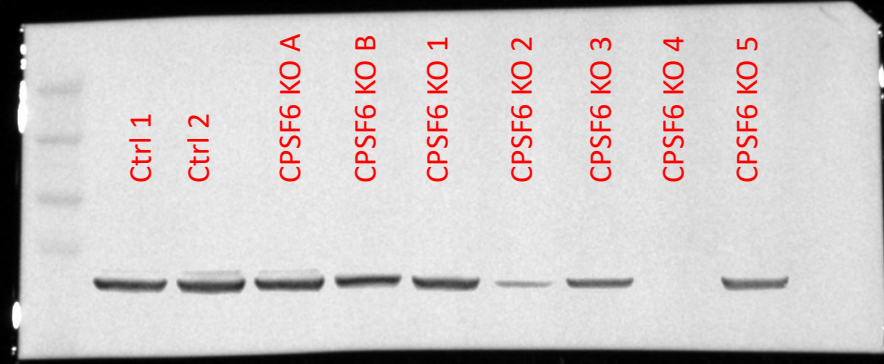

CPSF6

Supplement: Figure 1—source data 4. [file elife-103725-fig1-data4.zip › Figure 1-source data 4/Figure 1C-WB with annotations.pdf]

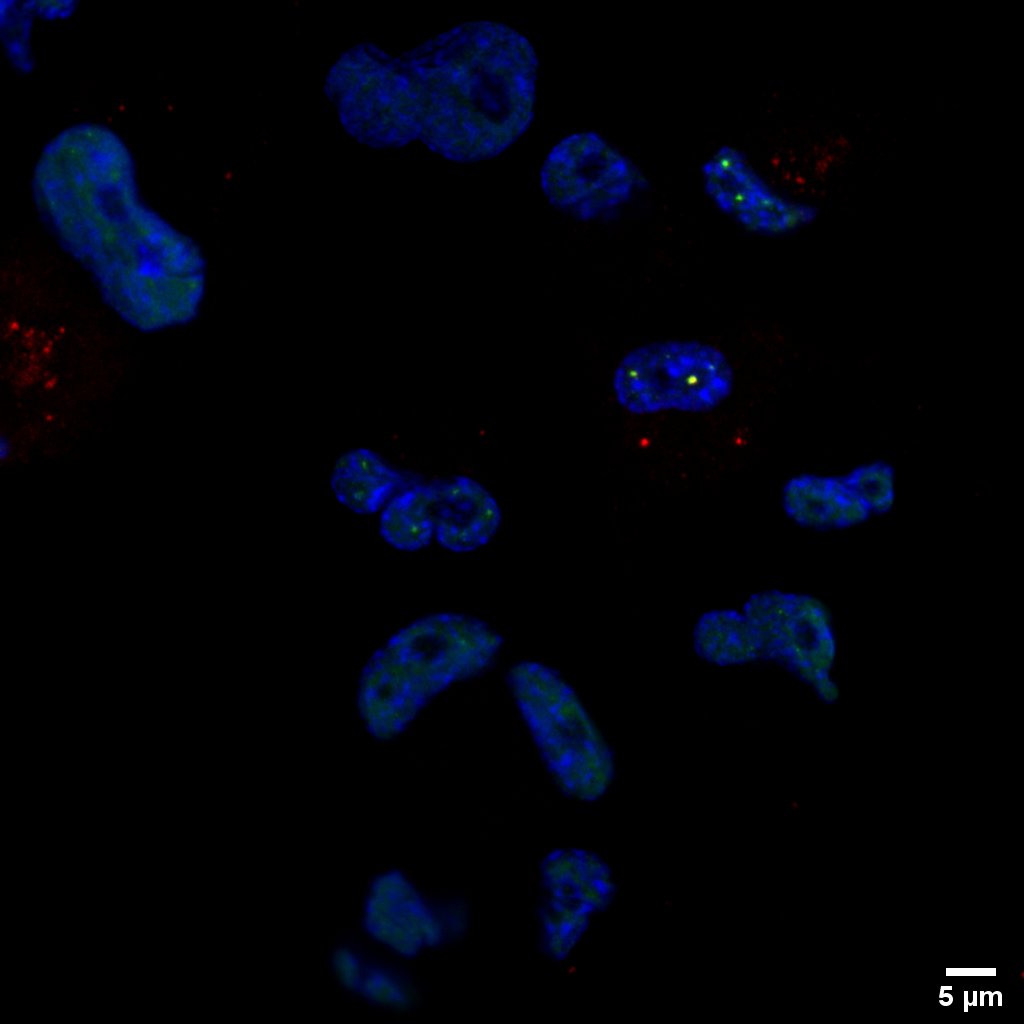

Supplement: Figure 1—source data 5. [file elife-103725-fig1-data5.zip › Figure 1-source data 5/Figure 1D/Control cells.jpg]

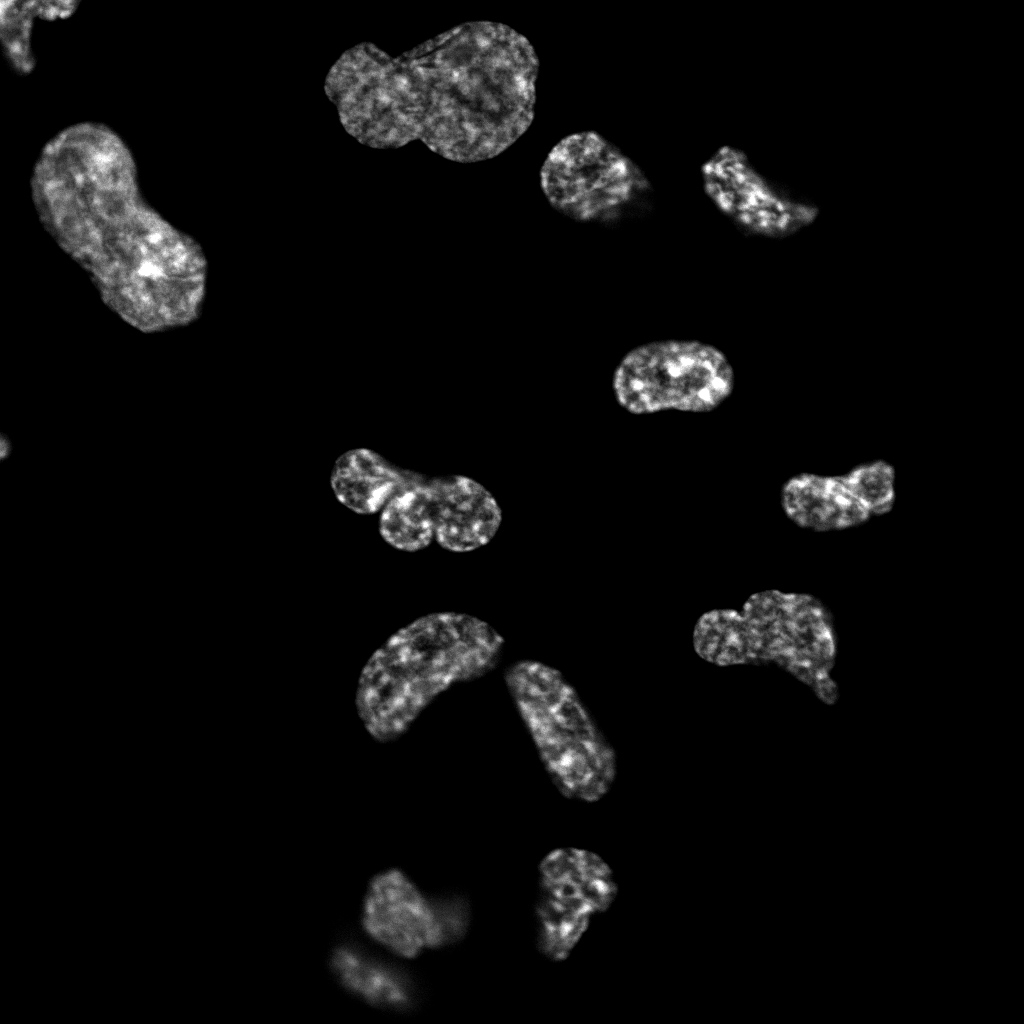

Supplement: Figure 1—source data 5. [file elife-103725-fig1-data5.zip › Figure 1-source data 5/Figure 1D/Control cells.tif]

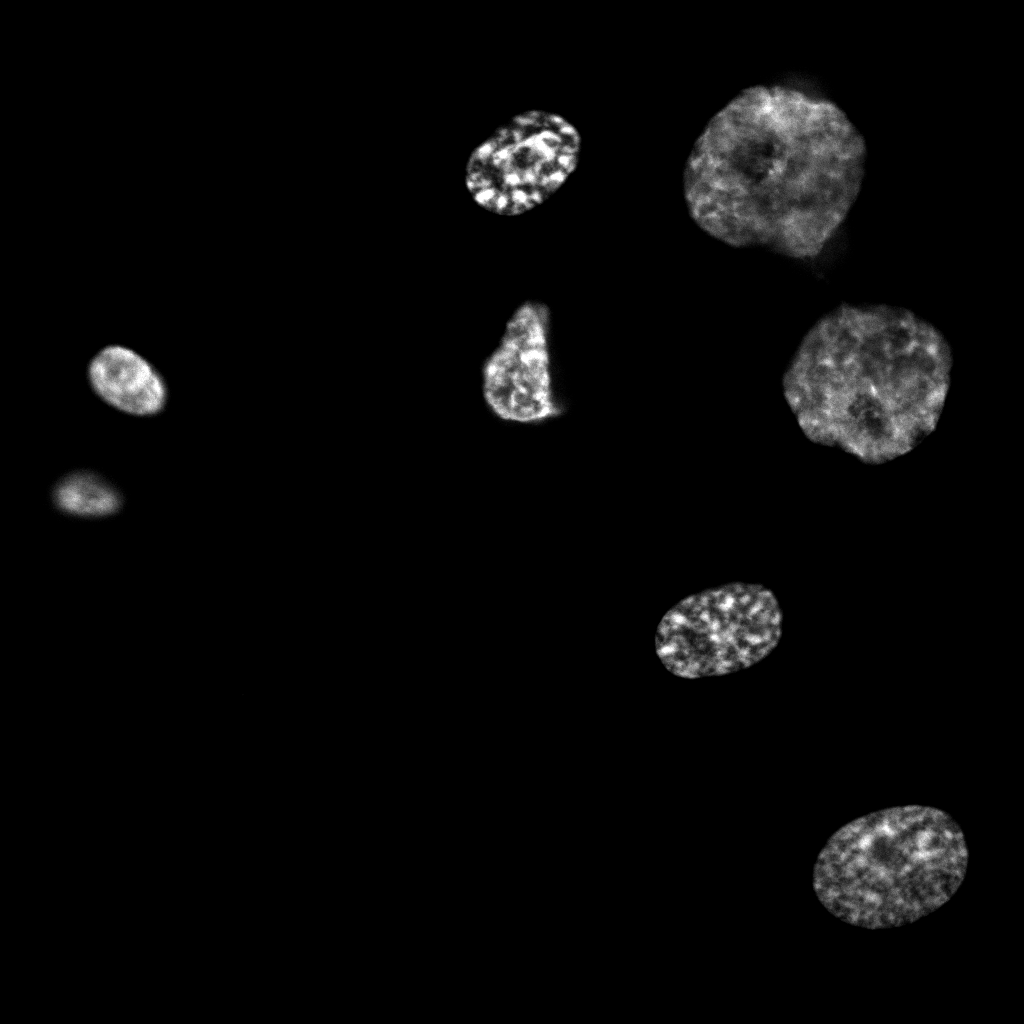

Supplement: Figure 1—source data 5. [file elife-103725-fig1-data5.zip › Figure 1-source data 5/Figure 1D/KO cells - NT - 447vpx n8Large.tif]

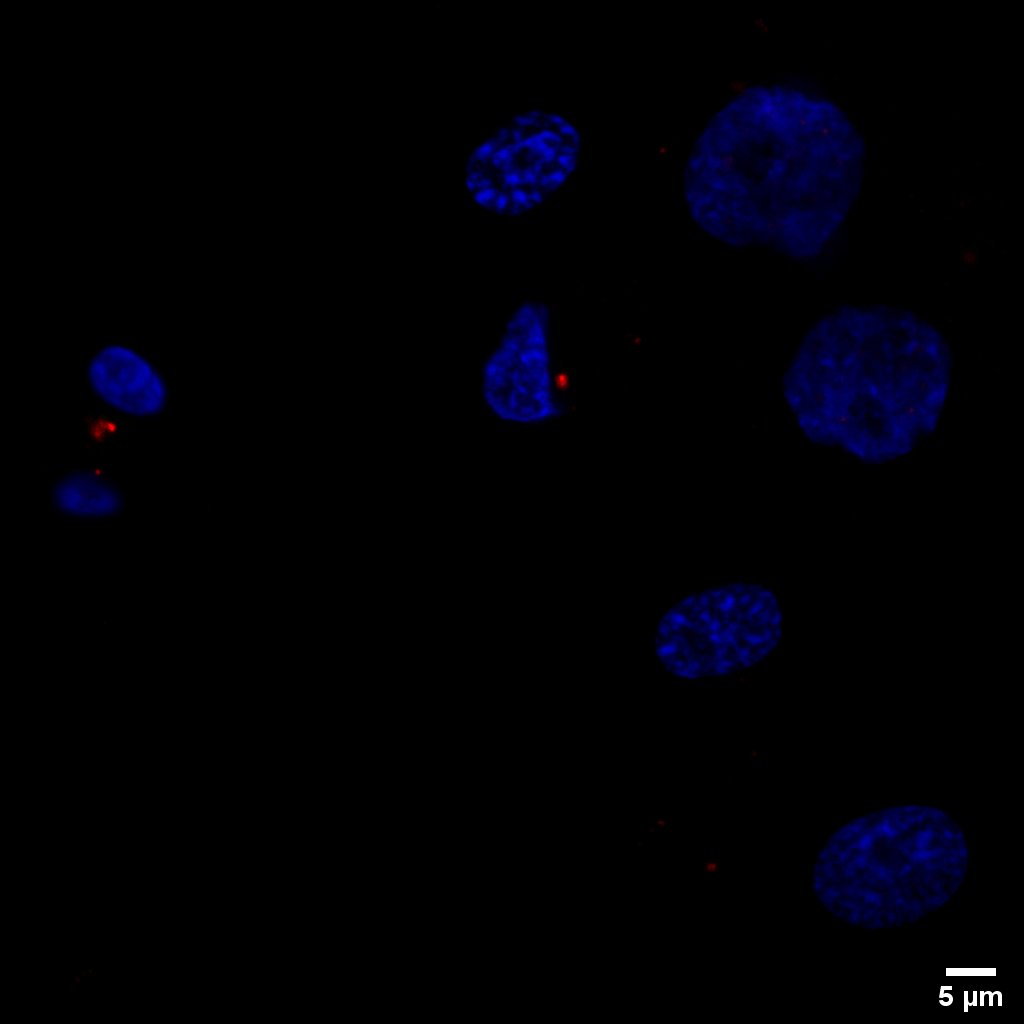

Supplement: Figure 1—source data 5. [file elife-103725-fig1-data5.zip › Figure 1-source data 5/Figure 1D/KO cells.jpg]

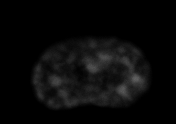

Supplement: Figure 1—source data 6. [file elife-103725-fig1-data6.zip › Figure 1-source data 6/Figure 1D-crops/Control cells - NT - 447vpx n8.tif]

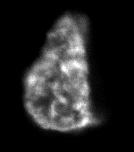

Supplement: Figure 1—source data 6. [file elife-103725-fig1-data6.zip › Figure 1-source data 6/Figure 1D-crops/KO cells - NT - 447vpx n8.tif]

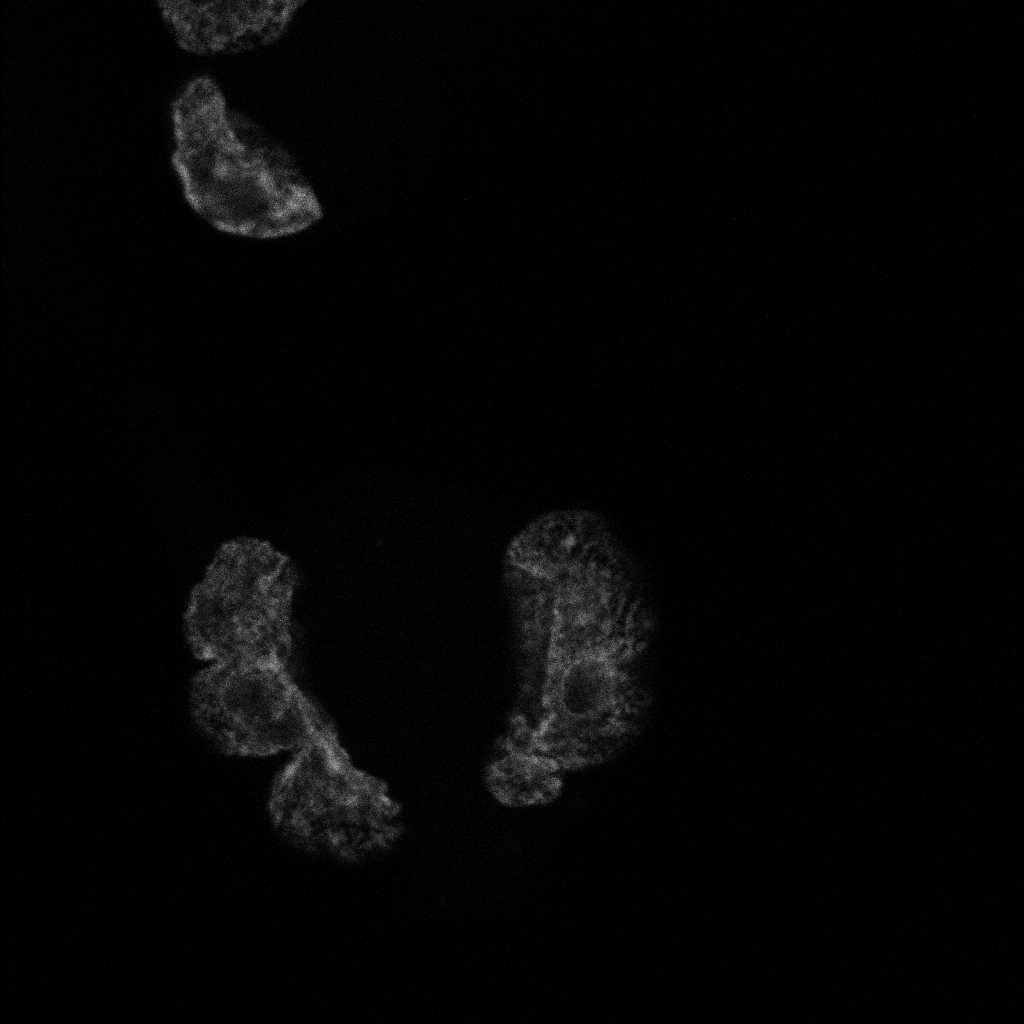

Supplement: Figure 2—source data 2. [file elife-103725-fig2-data2.zip › Figure 2-source data 2/Figure 2B/CPSF6 3XNLS deltaMCD.tif]

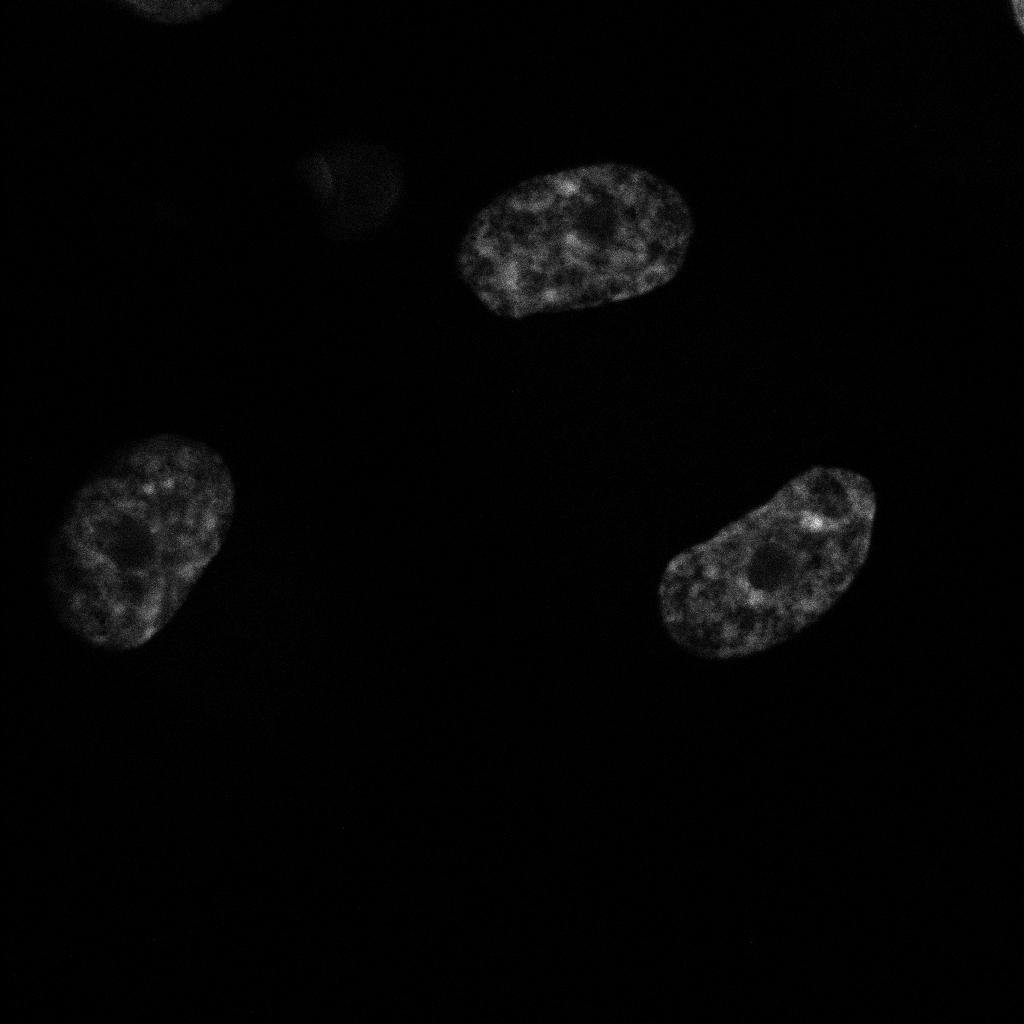

Supplement: Figure 2—source data 2. [file elife-103725-fig2-data2.zip › Figure 2-source data 2/Figure 2B/CPSF6 ADD2 delta LCR.tif]

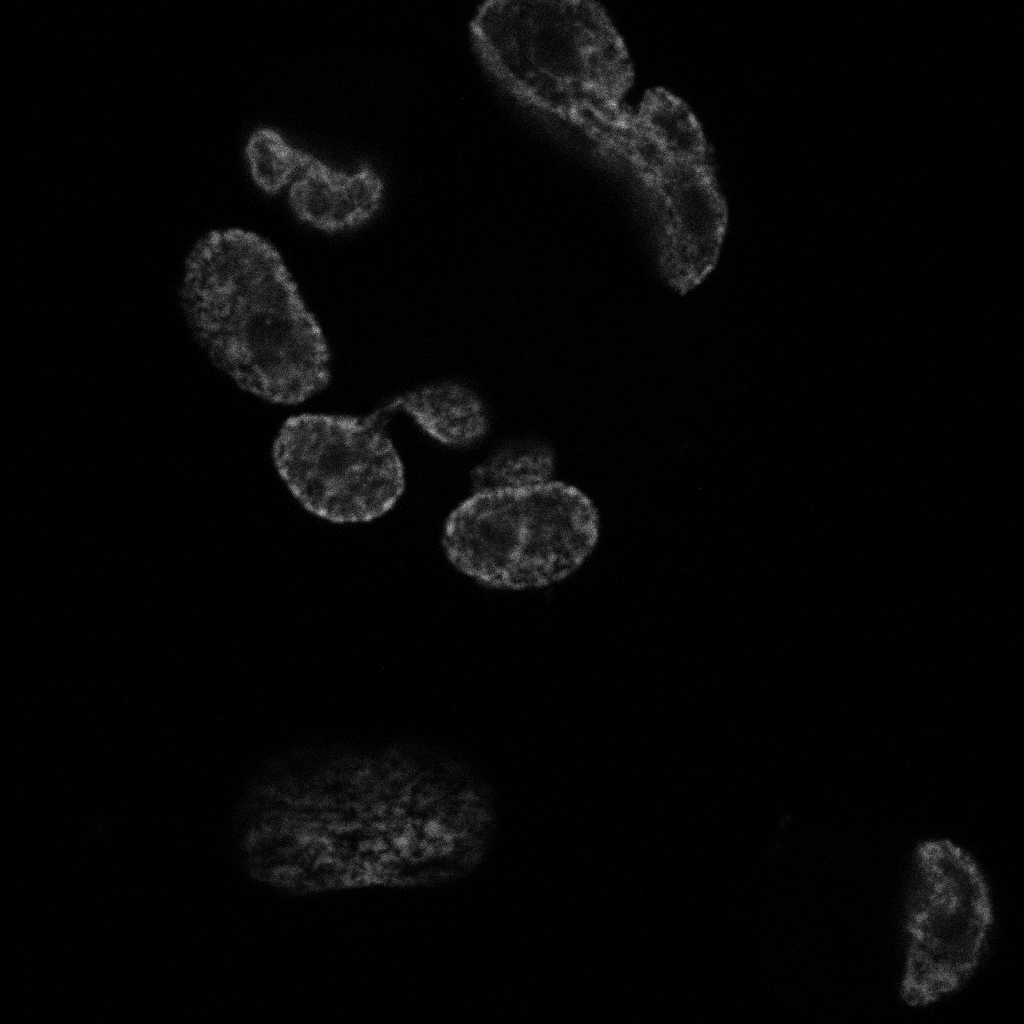

Supplement: Figure 2—source data 2. [file elife-103725-fig2-data2.zip › Figure 2-source data 2/Figure 2B/CPSF6 delta FG.tif]

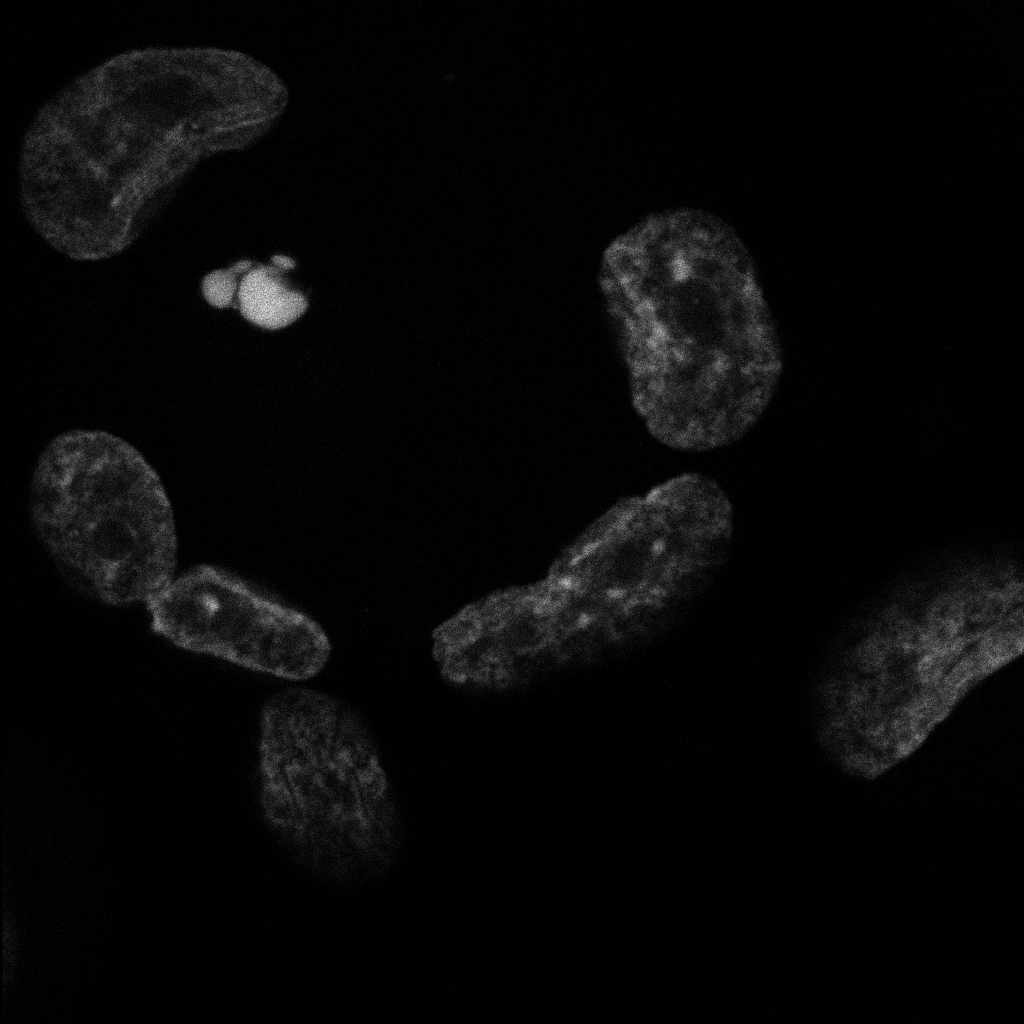

Supplement: Figure 2—source data 2. [file elife-103725-fig2-data2.zip › Figure 2-source data 2/Figure 2B/CPSF6 delta LCR.tif]

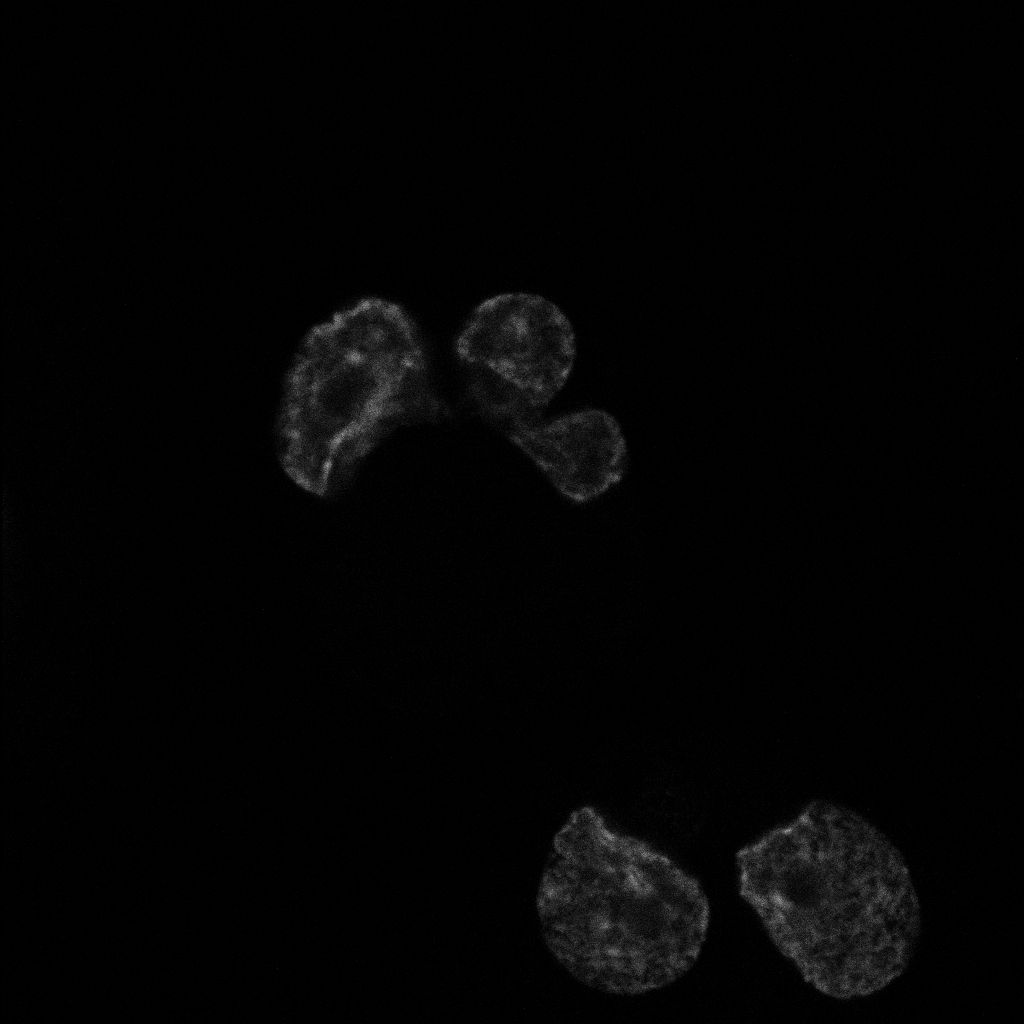

Supplement: Figure 2—source data 2. [file elife-103725-fig2-data2.zip › Figure 2-source data 2/Figure 2B/CPSF6 deltaFG delta LCR.tif]

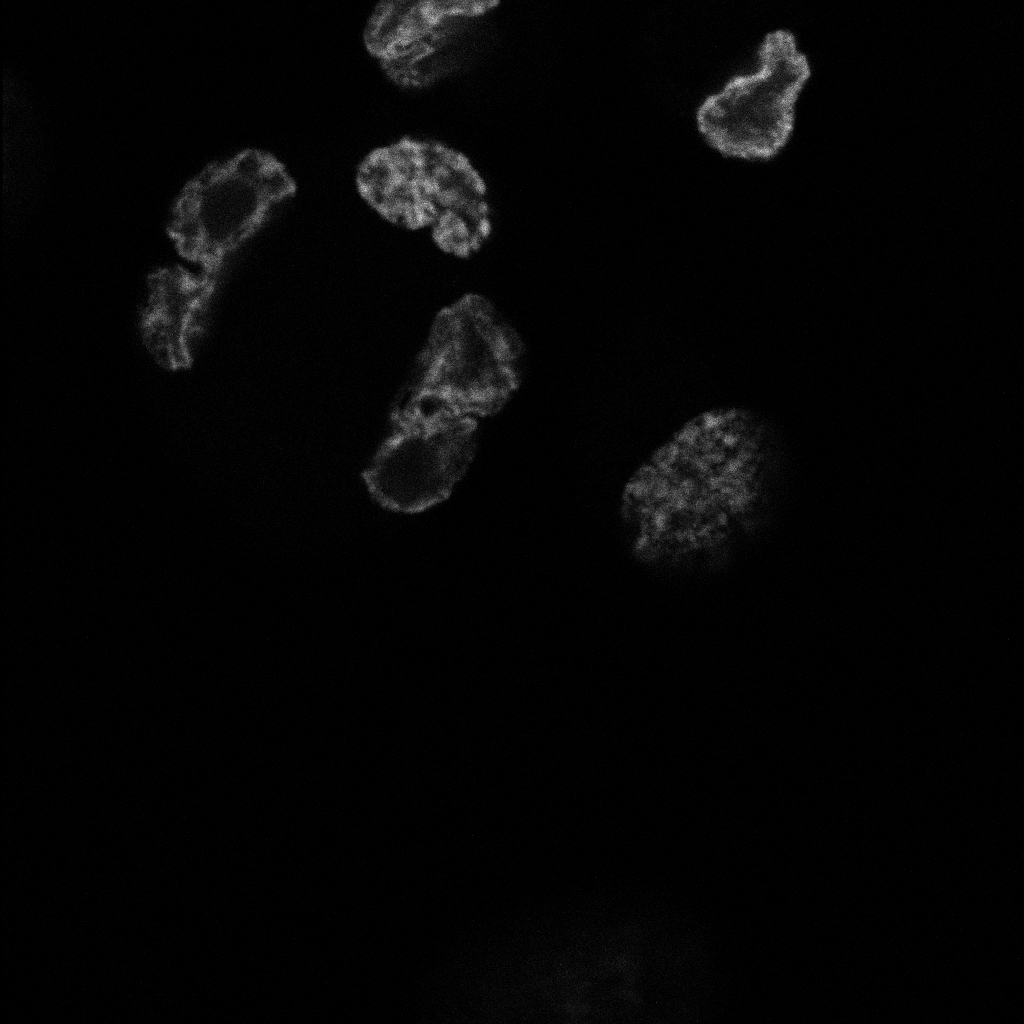

Supplement: Figure 2—source data 2. [file elife-103725-fig2-data2.zip › Figure 2-source data 2/Figure 2B/CPSF6 WT.tif]

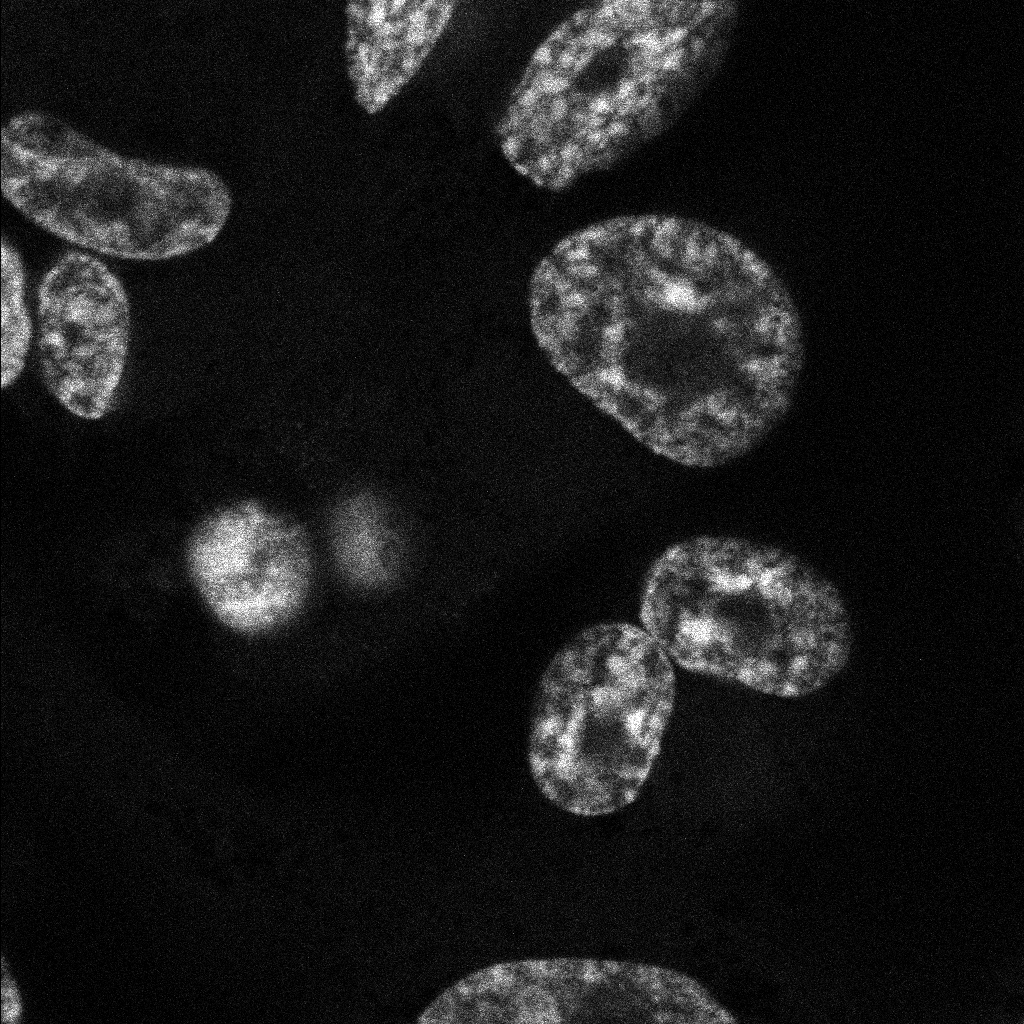

Supplement: Figure 2—source data 3. [file elife-103725-fig2-data3.zip › Figure 2-source data 3/Figure 2E/THP-1 CPSF6 DeltaMCD 3NLS.tif]

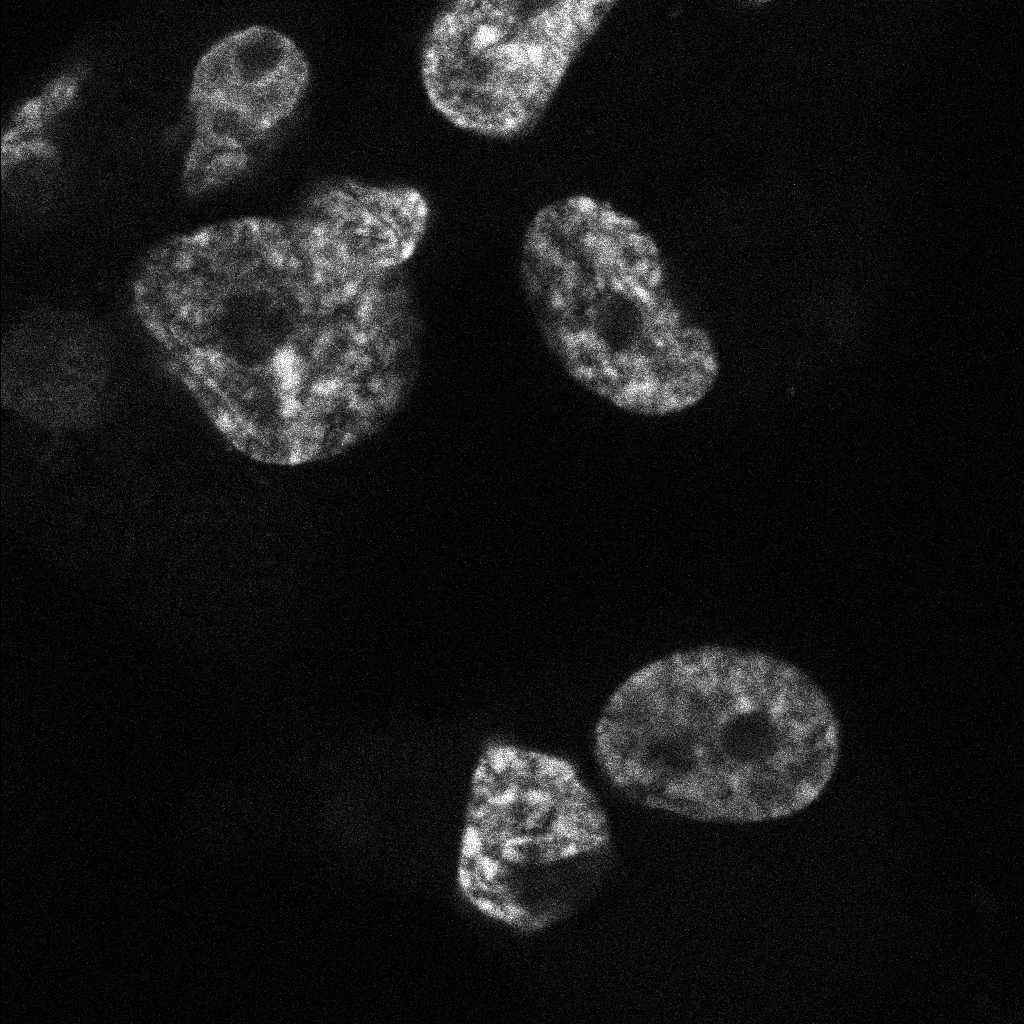

Supplement: Figure 2—source data 3. [file elife-103725-fig2-data3.zip › Figure 2-source data 3/Figure 2E/THP-1 CPSF6 KO.tif]

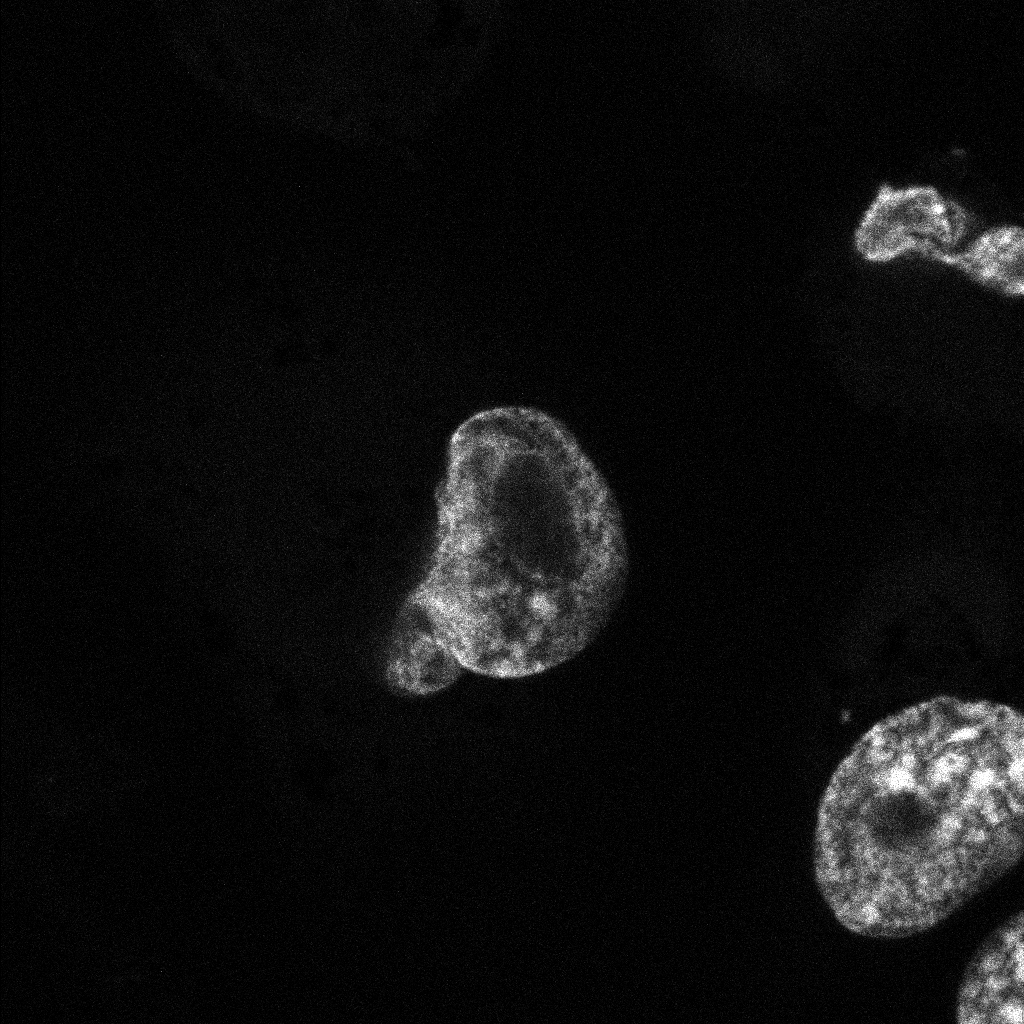

Supplement: Figure 2—source data 3. [file elife-103725-fig2-data3.zip › Figure 2-source data 3/Figure 2E/THP-1 CPSF6 WT.tif]

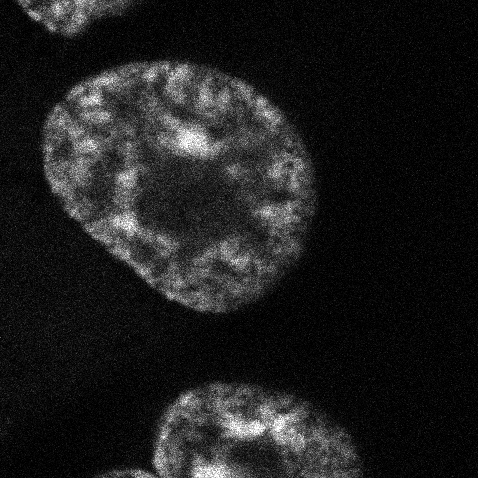

Supplement: Figure 2—source data 4. [file elife-103725-fig2-data4.zip › Figure 2-source data 4/Figure 2E crops/THP-1 CPSF6 DeltaMCD 3NLS-crop and scale.tif]

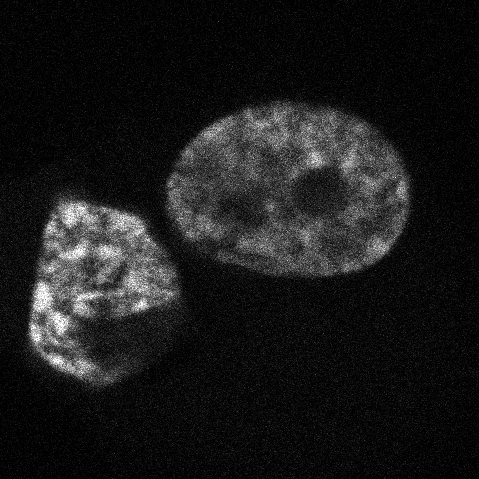

Supplement: Figure 2—source data 4. [file elife-103725-fig2-data4.zip › Figure 2-source data 4/Figure 2E crops/THP-1 CPSF6 KO-crop and scale.tif]

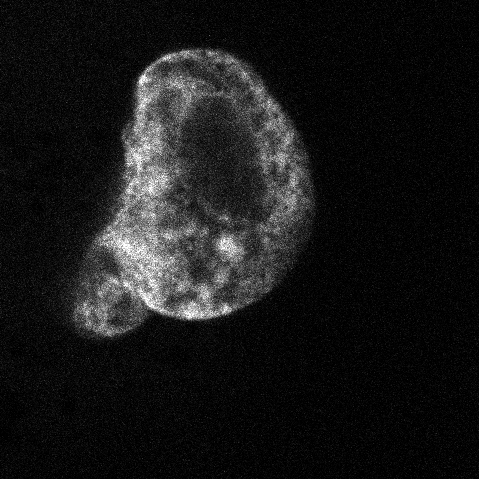

Supplement: Figure 2—source data 4. [file elife-103725-fig2-data4.zip › Figure 2-source data 4/Figure 2E crops/THP-1 CPSF6 WT-crop and scale.tif]

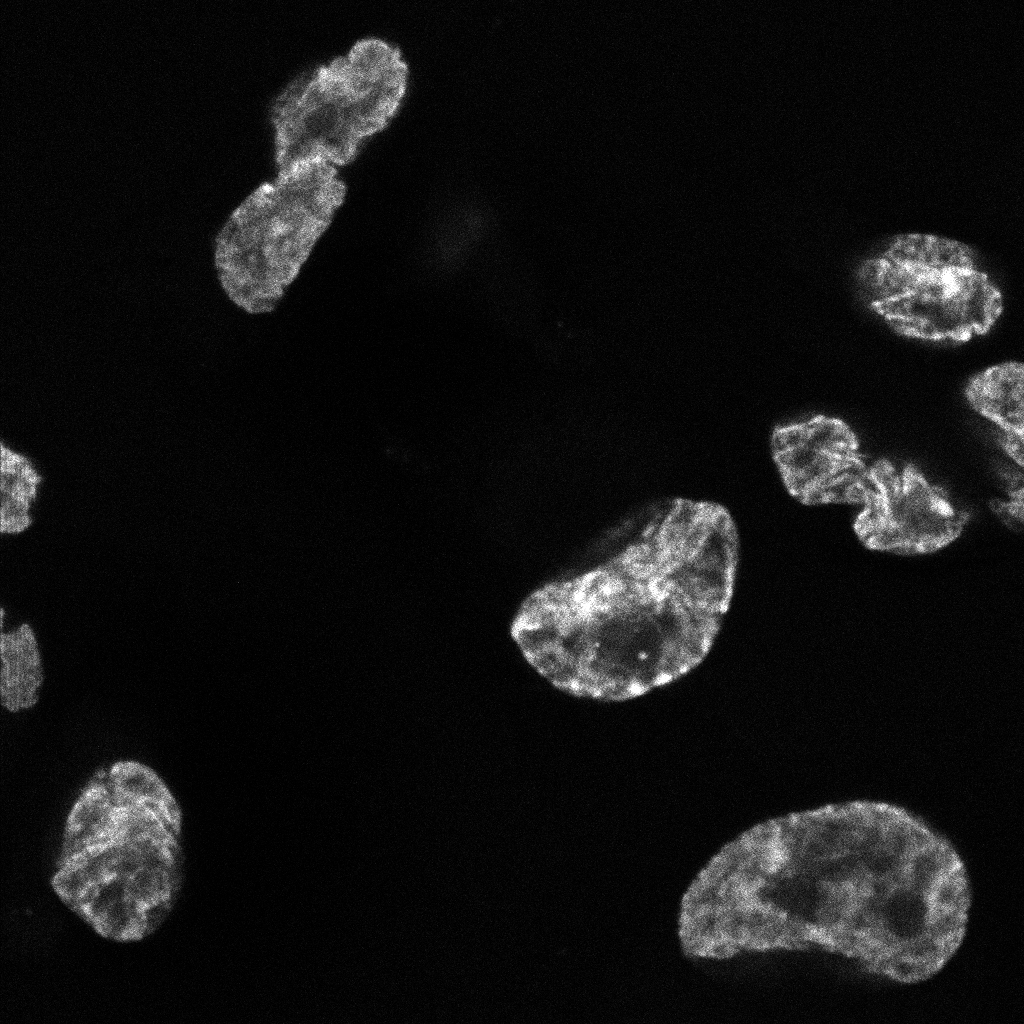

Supplement: Figure 2—source data 5. [file elife-103725-fig2-data5.zip › Figure 2-source data 5/Figure 2F/Inf_CPSF6 DeltaMCD 3xNLS.tif]

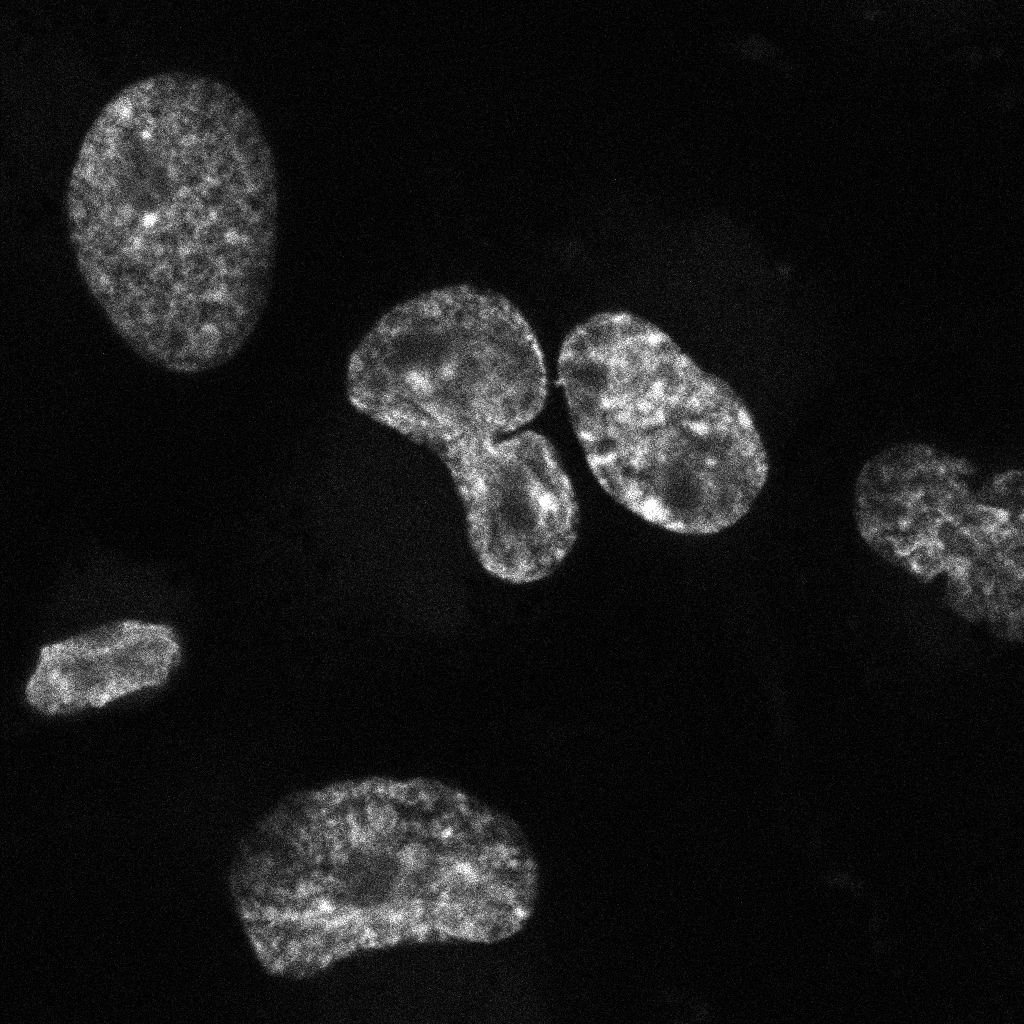

Supplement: Figure 2—source data 5. [file elife-103725-fig2-data5.zip › Figure 2-source data 5/Figure 2F/Inf_CPSF6 DeltaMCD PYNLS.tif]

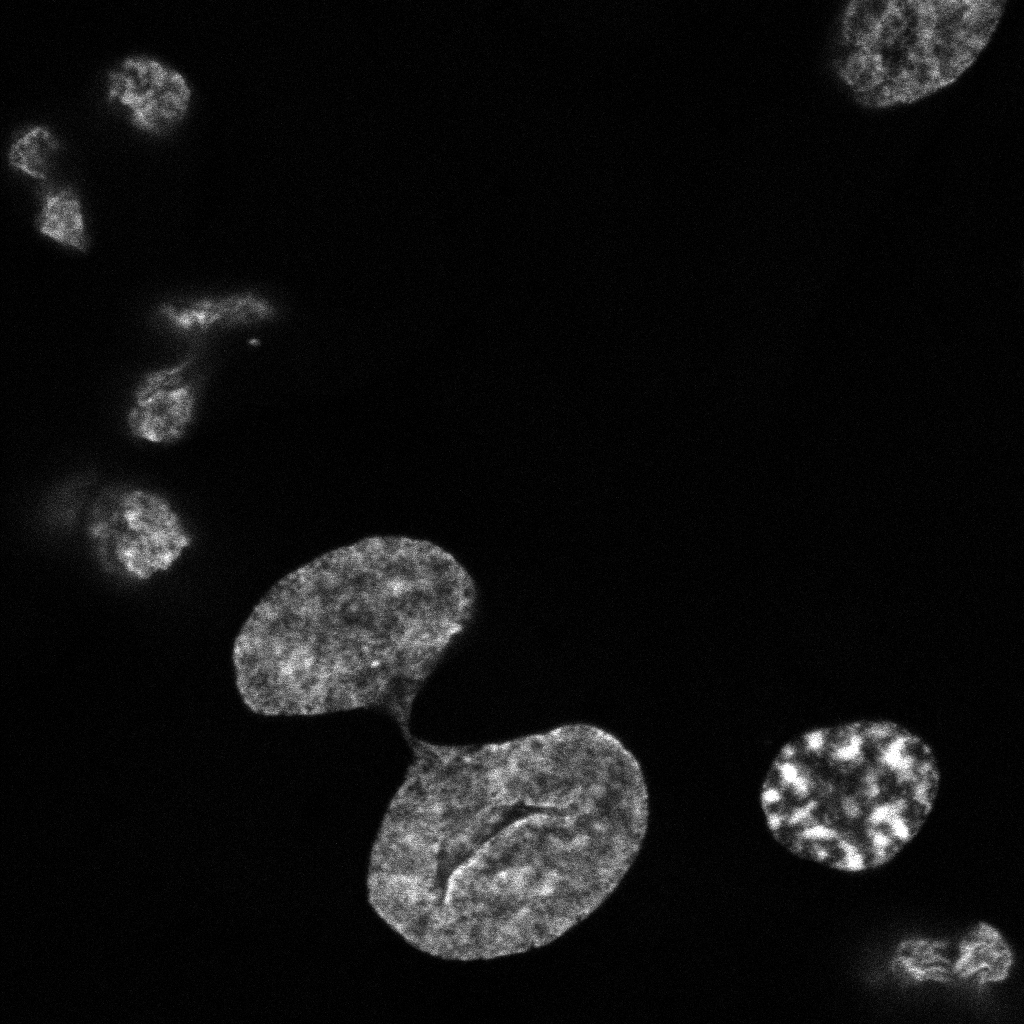

Supplement: Figure 2—source data 5. [file elife-103725-fig2-data5.zip › Figure 2-source data 5/Figure 2F/Inf_CPSF6 DeltaMCD.tif]

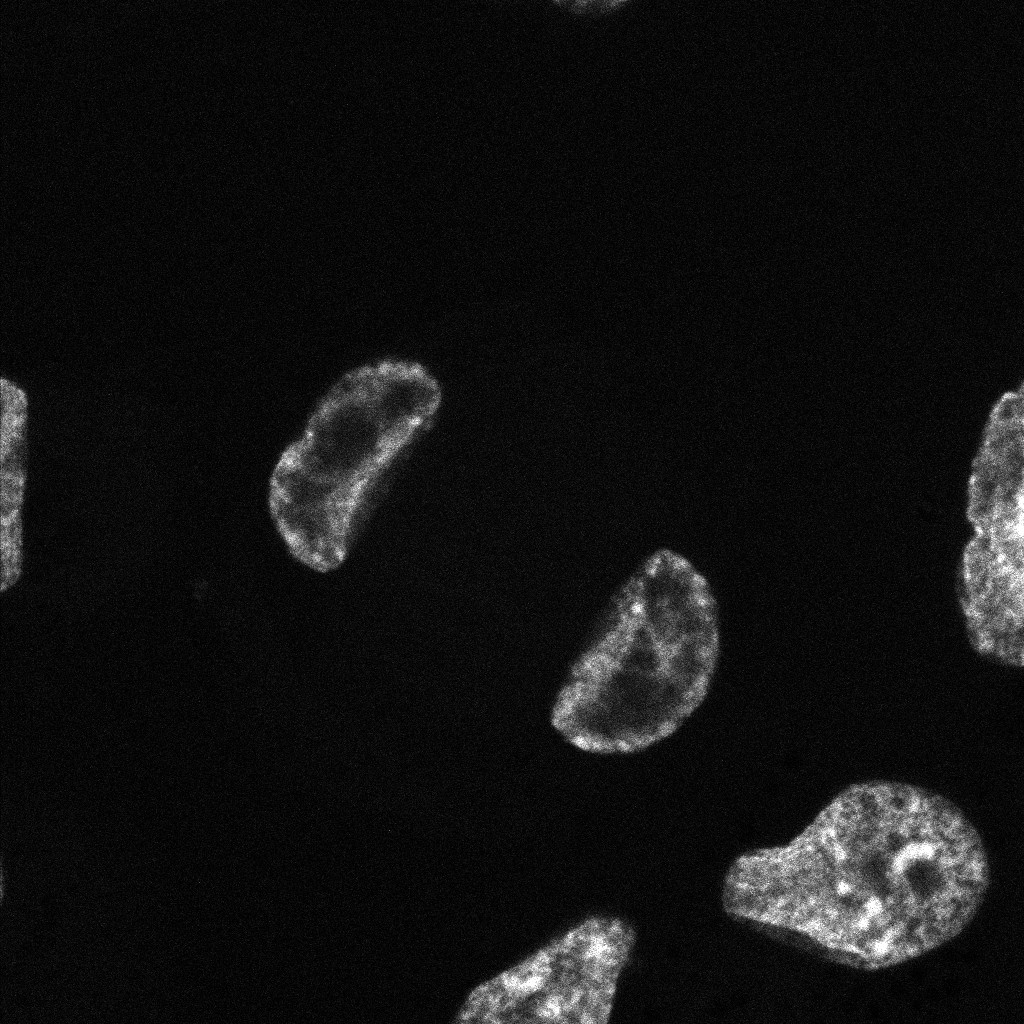

Supplement: Figure 2—source data 5. [file elife-103725-fig2-data5.zip › Figure 2-source data 5/Figure 2F/Inf_CPSF6 WT.tif]

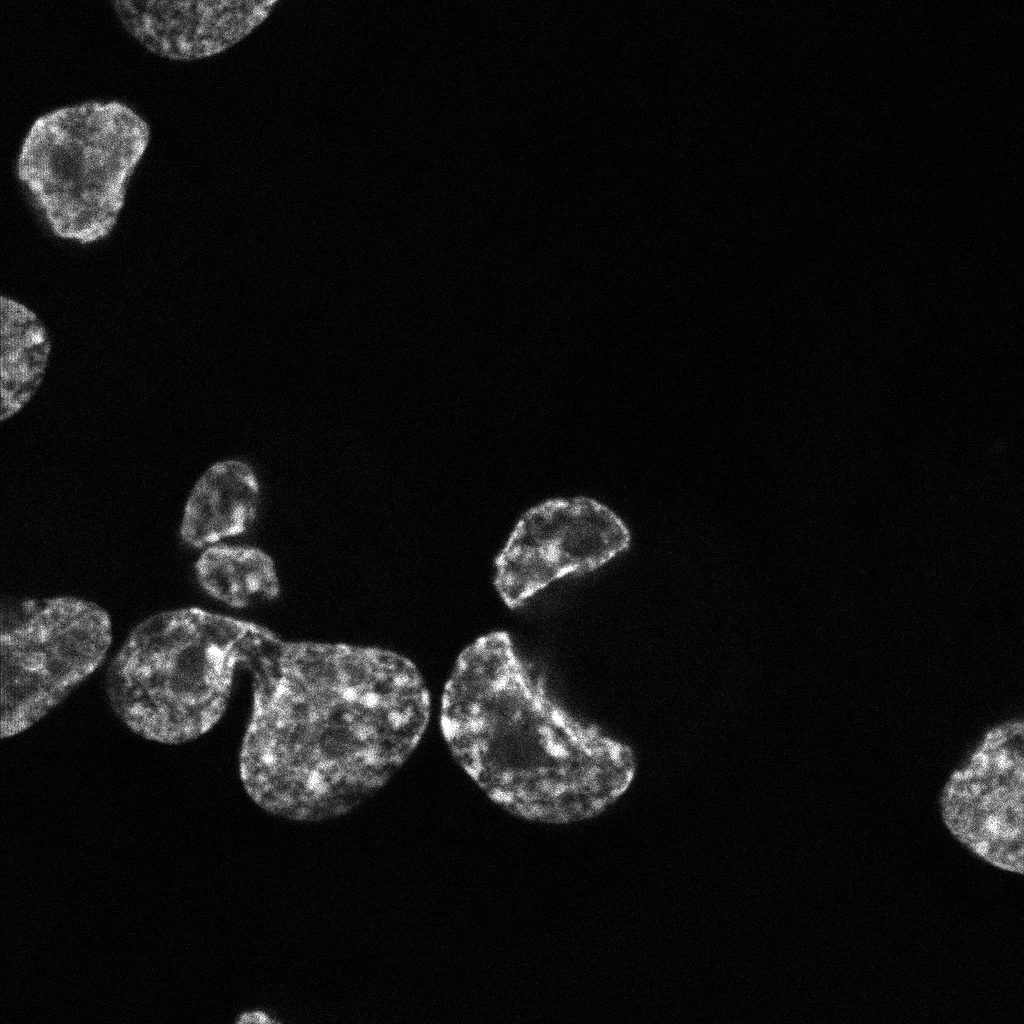

Supplement: Figure 2—source data 5. [file elife-103725-fig2-data5.zip › Figure 2-source data 5/Figure 2F/NonInf_CPSF6 DeltaMCD 3xNLS.tif]

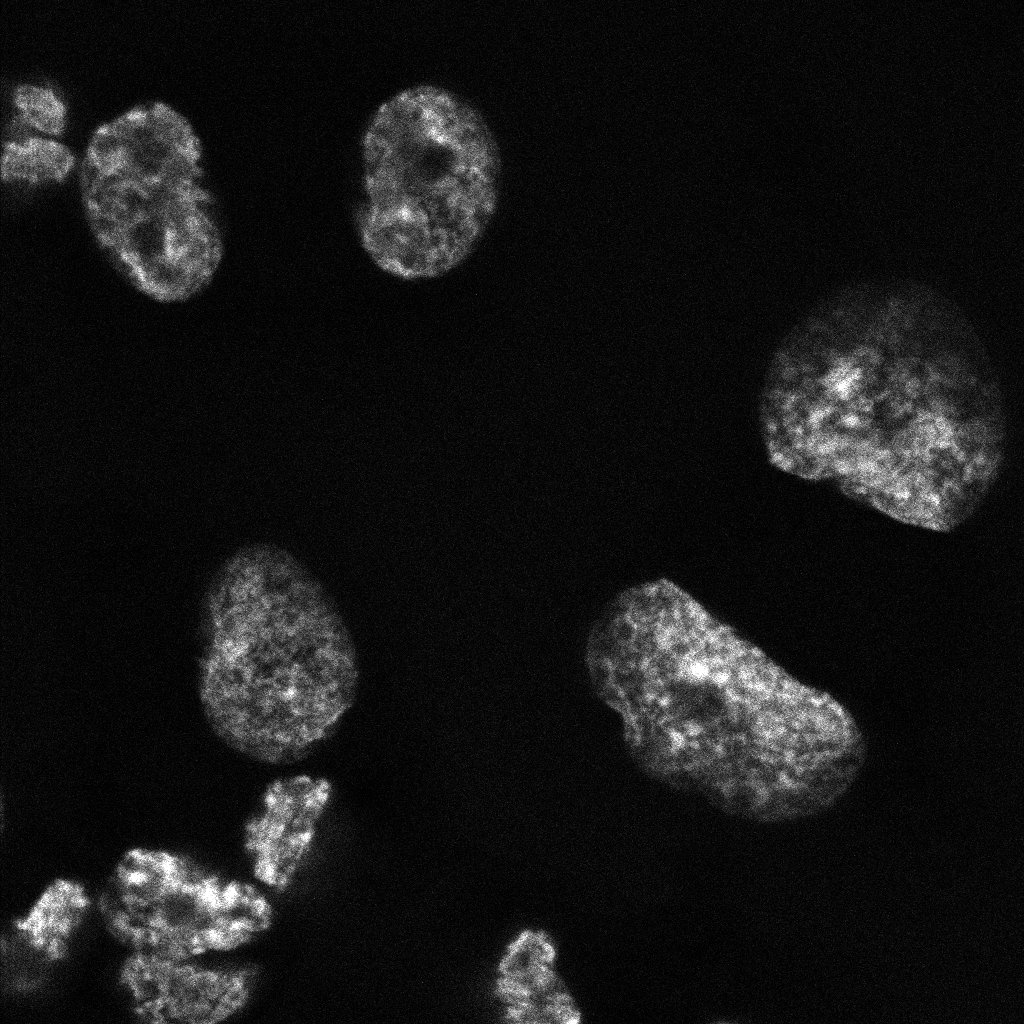

Supplement: Figure 2—source data 5. [file elife-103725-fig2-data5.zip › Figure 2-source data 5/Figure 2F/NonInf_CPSF6 DeltaMCD PY NLS.tif]

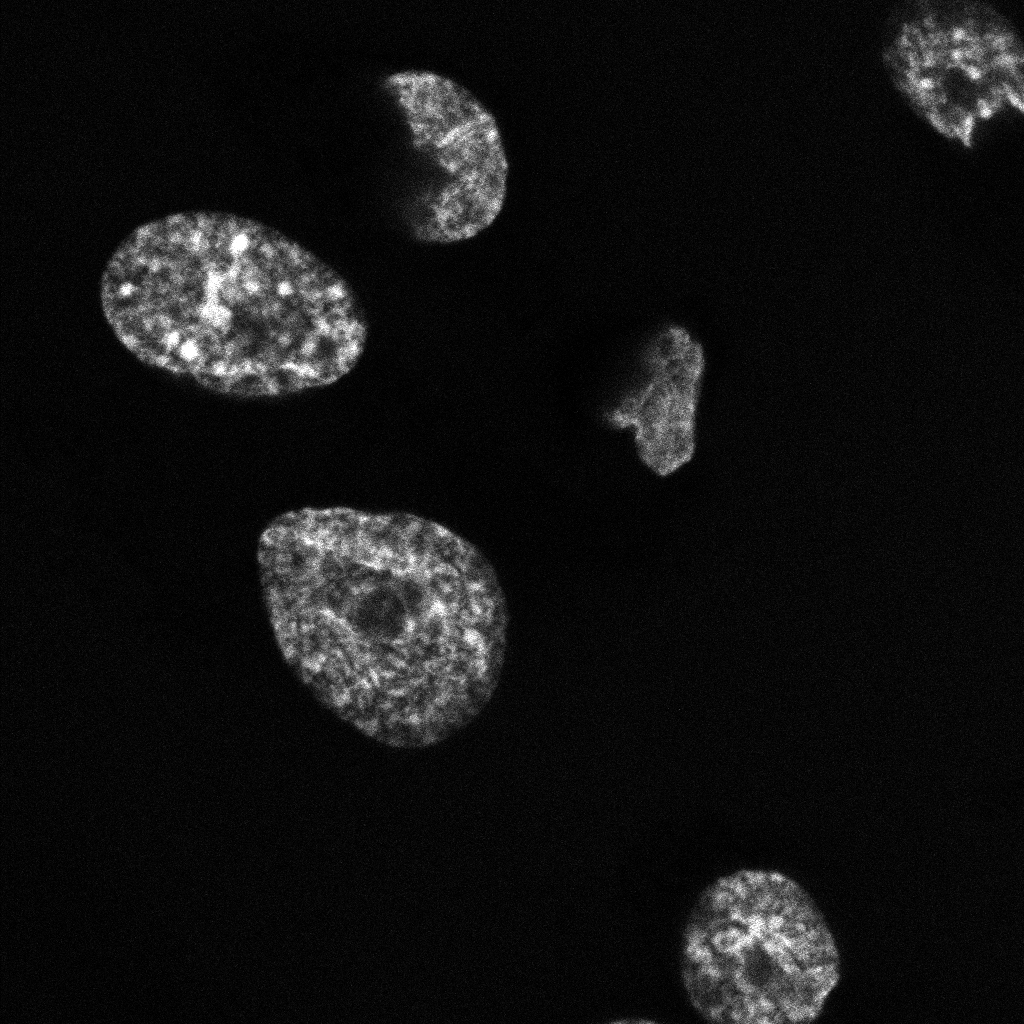

Supplement: Figure 2—source data 5. [file elife-103725-fig2-data5.zip › Figure 2-source data 5/Figure 2F/NonInf_CPSF6 DeltaMCD.tif]

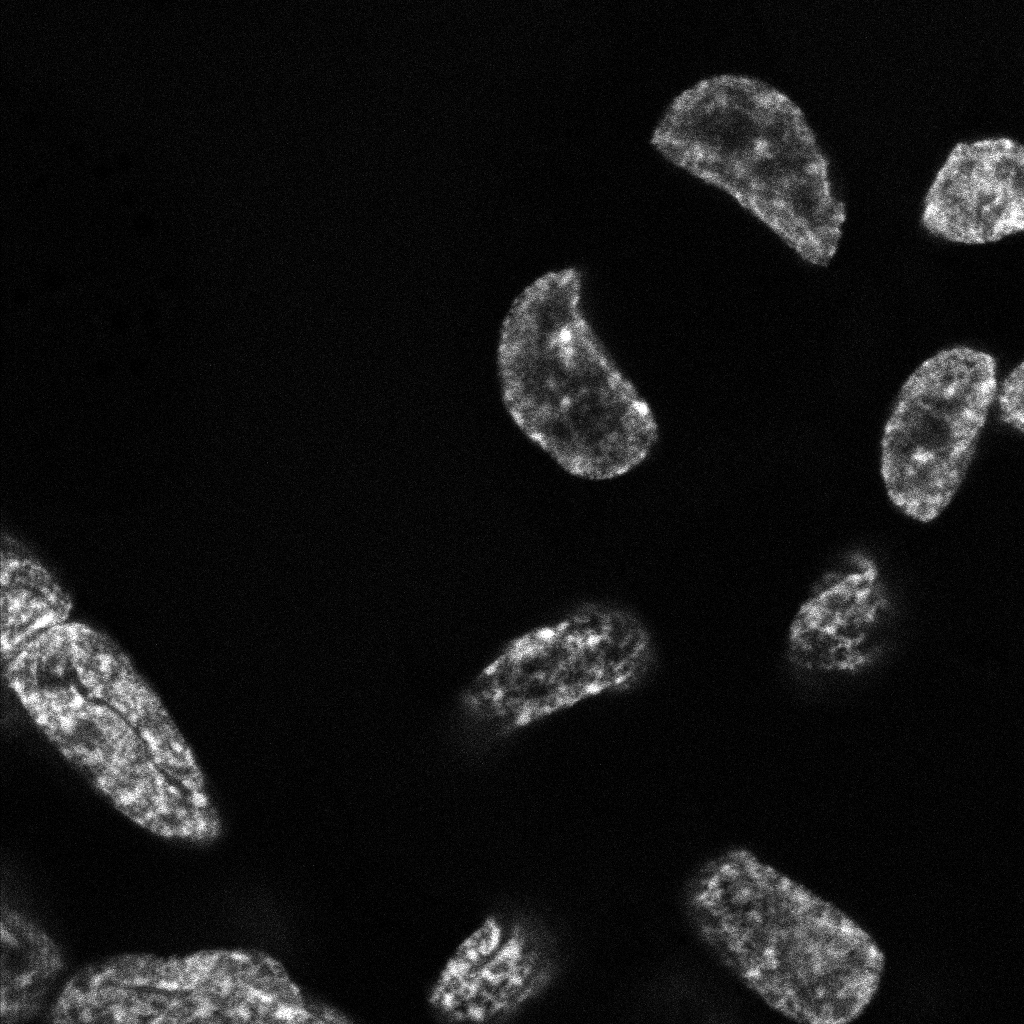

Supplement: Figure 2—source data 5. [file elife-103725-fig2-data5.zip › Figure 2-source data 5/Figure 2F/NonInf_CPSF6 WT.tif]

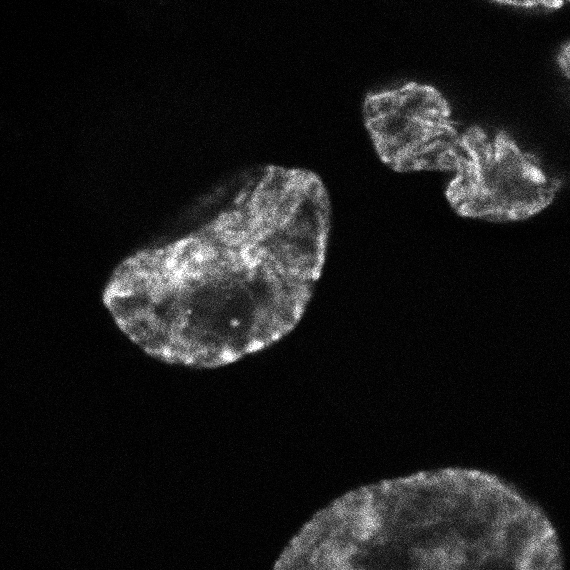

Supplement: Figure 2—source data 6. [file elife-103725-fig2-data6.zip › Figure 2-source data 6/Figure 2F crops/Inf_CPSF6 DeltaMCD 3xNLS.tif]

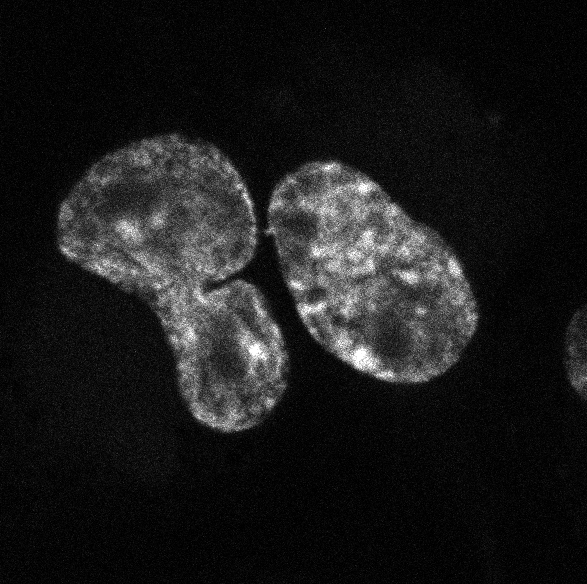

Supplement: Figure 2—source data 6. [file elife-103725-fig2-data6.zip › Figure 2-source data 6/Figure 2F crops/Inf_CPSF6 DeltaMCD PYNLS.tif]

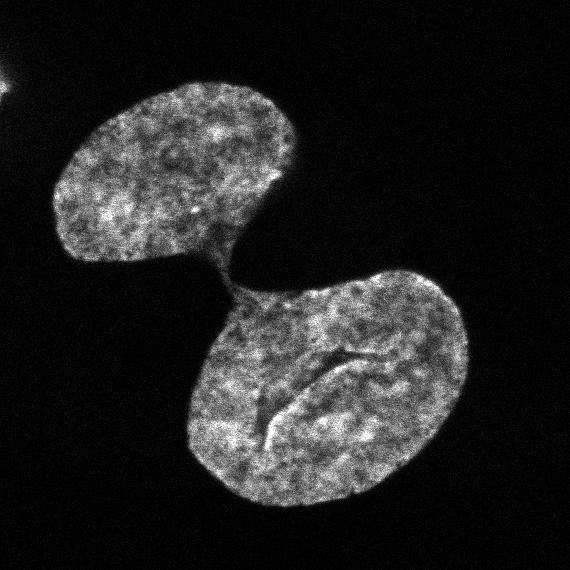

Supplement: Figure 2—source data 6. [file elife-103725-fig2-data6.zip › Figure 2-source data 6/Figure 2F crops/Inf_CPSF6 DeltaMCD.tif]

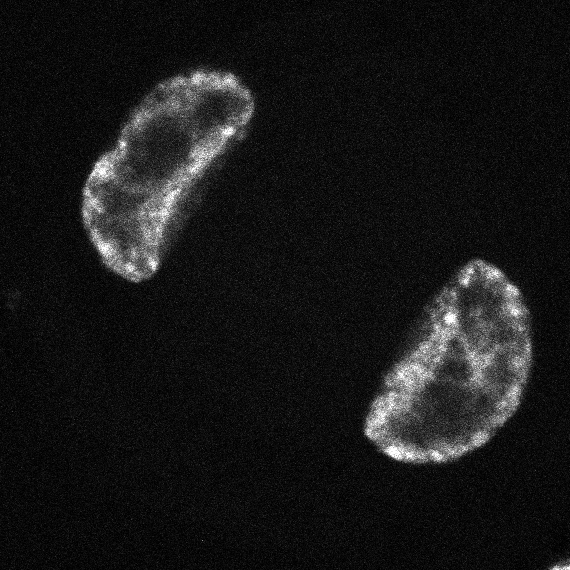

Supplement: Figure 2—source data 6. [file elife-103725-fig2-data6.zip › Figure 2-source data 6/Figure 2F crops/Inf_CPSF6 WT.tif]

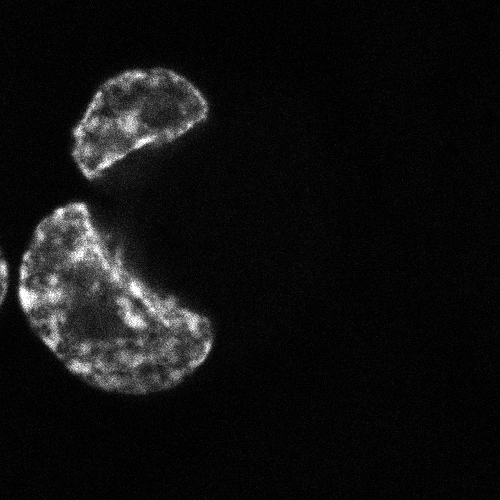

Supplement: Figure 2—source data 6. [file elife-103725-fig2-data6.zip › Figure 2-source data 6/Figure 2F crops/NonInf_CPSF6 DeltaMCD 3xNLS.tif]

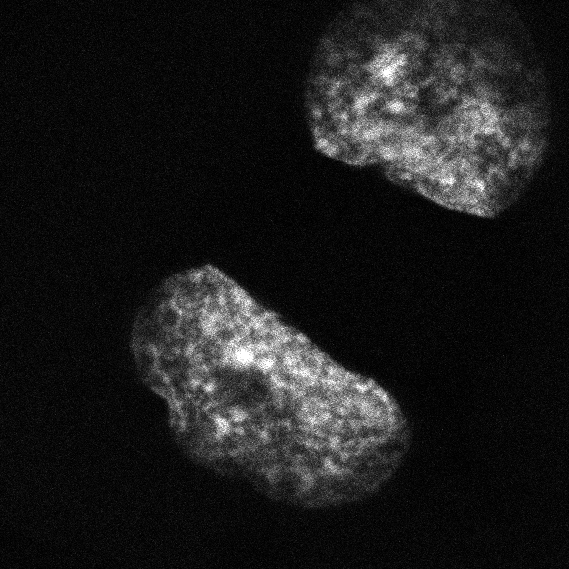

Supplement: Figure 2—source data 6. [file elife-103725-fig2-data6.zip › Figure 2-source data 6/Figure 2F crops/NonInf_CPSF6 DeltaMCD PY NLS.tif]

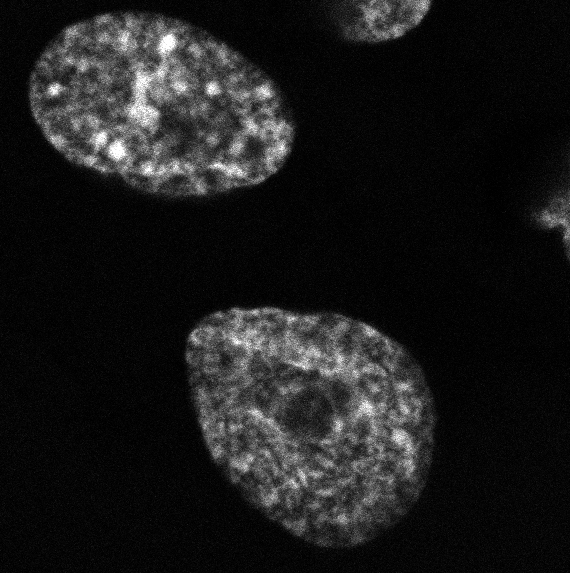

Supplement: Figure 2—source data 6. [file elife-103725-fig2-data6.zip › Figure 2-source data 6/Figure 2F crops/NonInf_CPSF6 DeltaMCD.tif]

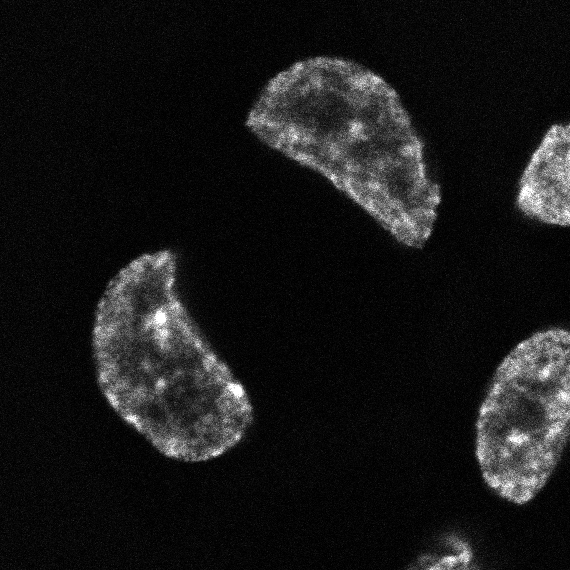

Supplement: Figure 2—source data 6. [file elife-103725-fig2-data6.zip › Figure 2-source data 6/Figure 2F crops/NonInf_CPSF6 WT.tif]

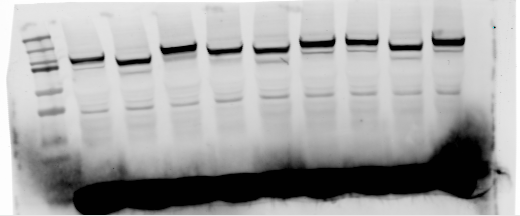

Supplement: Figure 3—source data 2. [file elife-103725-fig3-data2.zip › Figure 3-source data 2/Figure 3A WB/blot#1.tif]

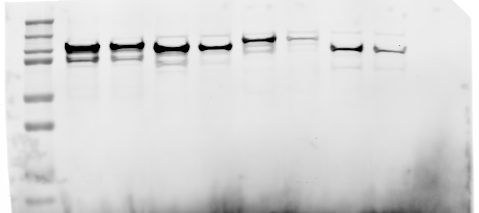

Supplement: Figure 3—source data 2. [file elife-103725-fig3-data2.zip › Figure 3-source data 2/Figure 3A WB/Blot#2.tif]

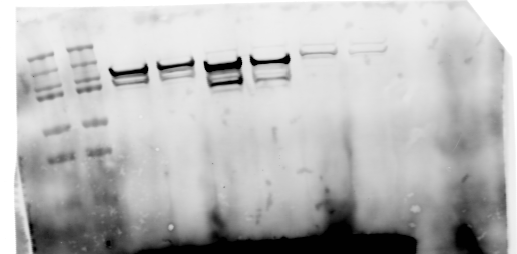

Supplement: Figure 3—source data 2. [file elife-103725-fig3-data2.zip › Figure 3-source data 2/Figure 3A WB/blot#3.tif]

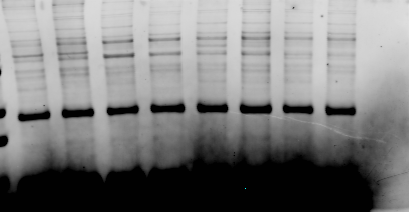

Supplement: Figure 3—source data 2. [file elife-103725-fig3-data2.zip › Figure 3-source data 2/Figure 3A WB/blot#4.tif]

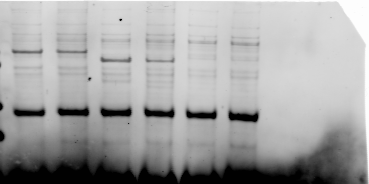

Supplement: Figure 3—source data 2. [file elife-103725-fig3-data2.zip › Figure 3-source data 2/Figure 3A WB/blot#5.tif]

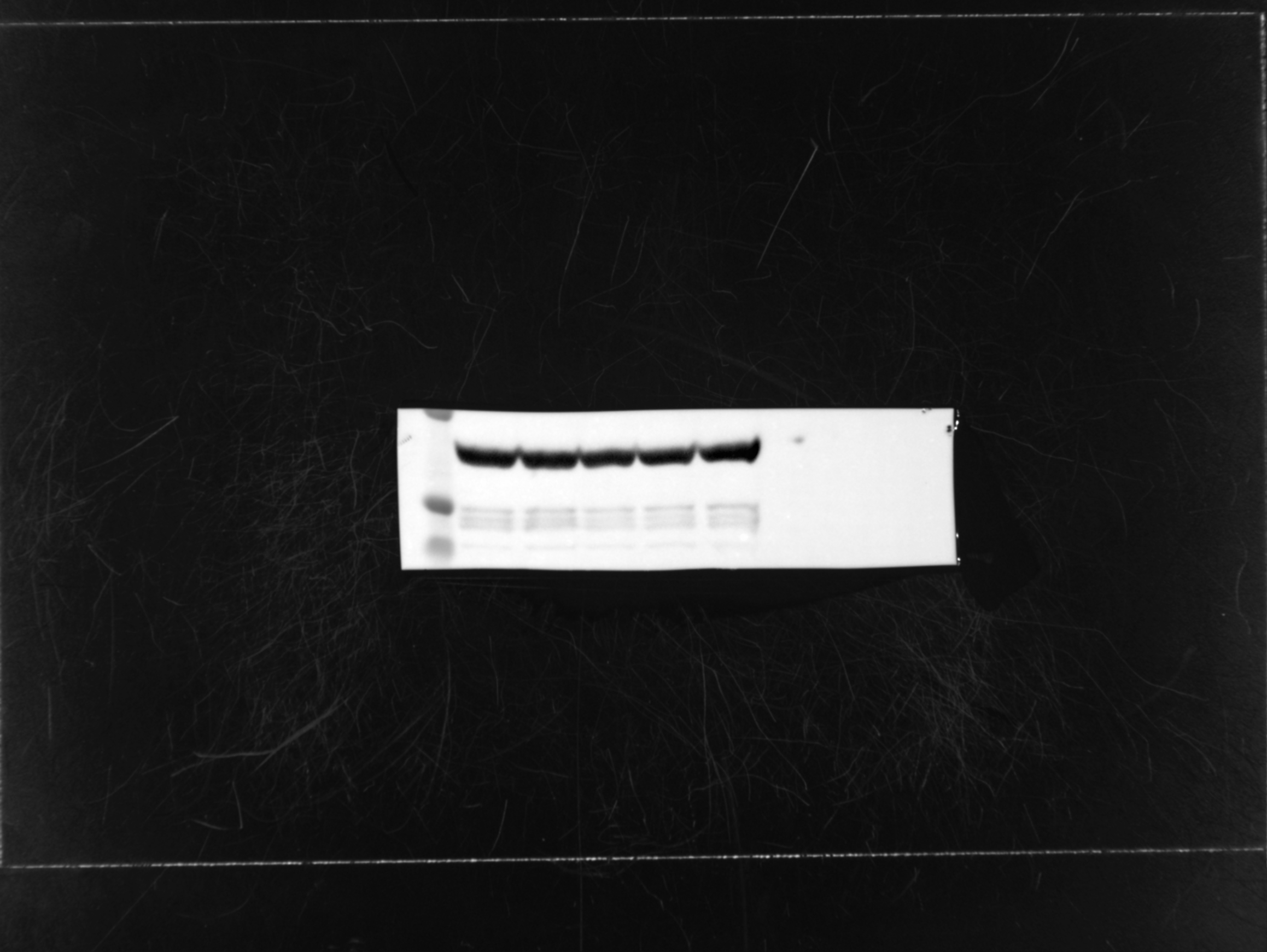

Supplement: Figure 5—source data 2. [file elife-103725-fig5-data2.zip › Figure 5-source data 2/Figure 5A/Actin_gel.tif]

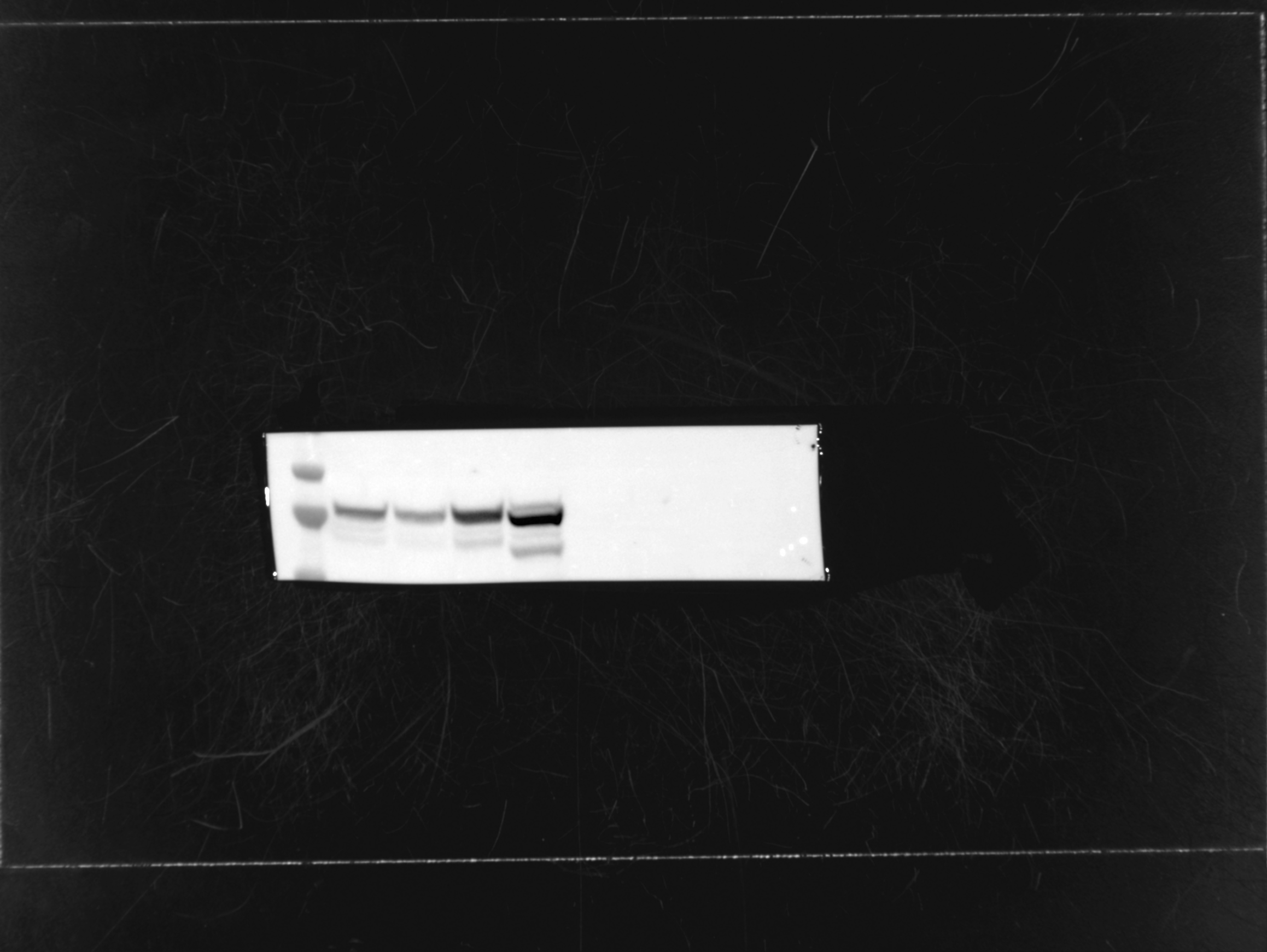

Supplement: Figure 5—source data 2. [file elife-103725-fig5-data2.zip › Figure 5-source data 2/Figure 5A/CPSF6_gel.tif]

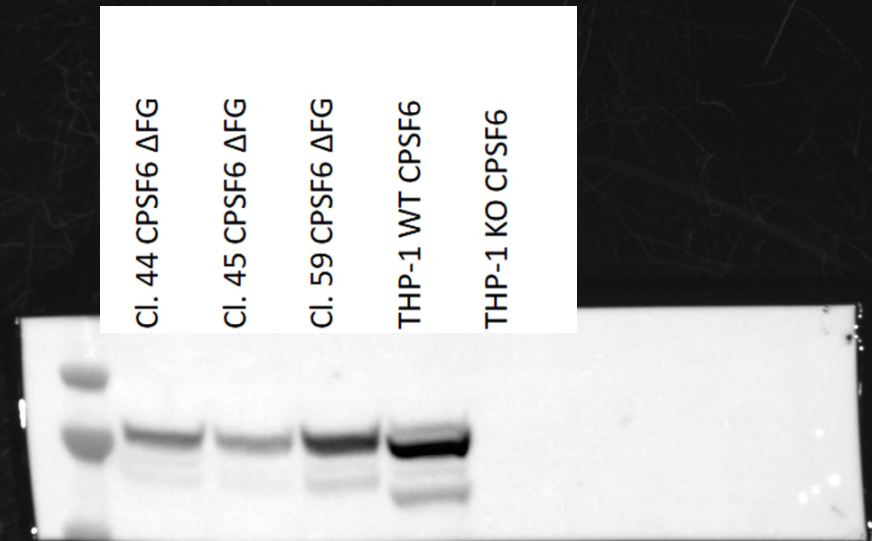

CPSF6

Cl. 44 CPSF6  $\Delta$ FG

Cl. 45 CPSF6  $\Delta$ FG

Cl. 59 CPSF6  $\Delta$ FG

THP-1 WT CPSF6

THP-1 KO CPSF6

Actin

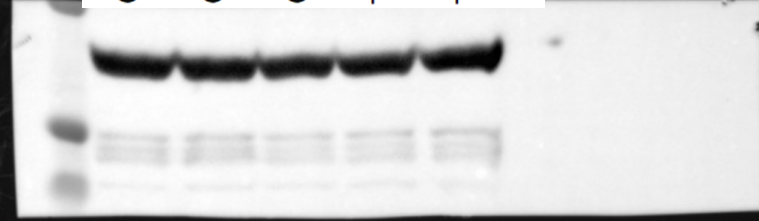

Supplement: Figure 5—source data 3. [file elife-103725-fig5-data3.zip › Figure 5-source data 3/Figure 5A annotated.pdf]

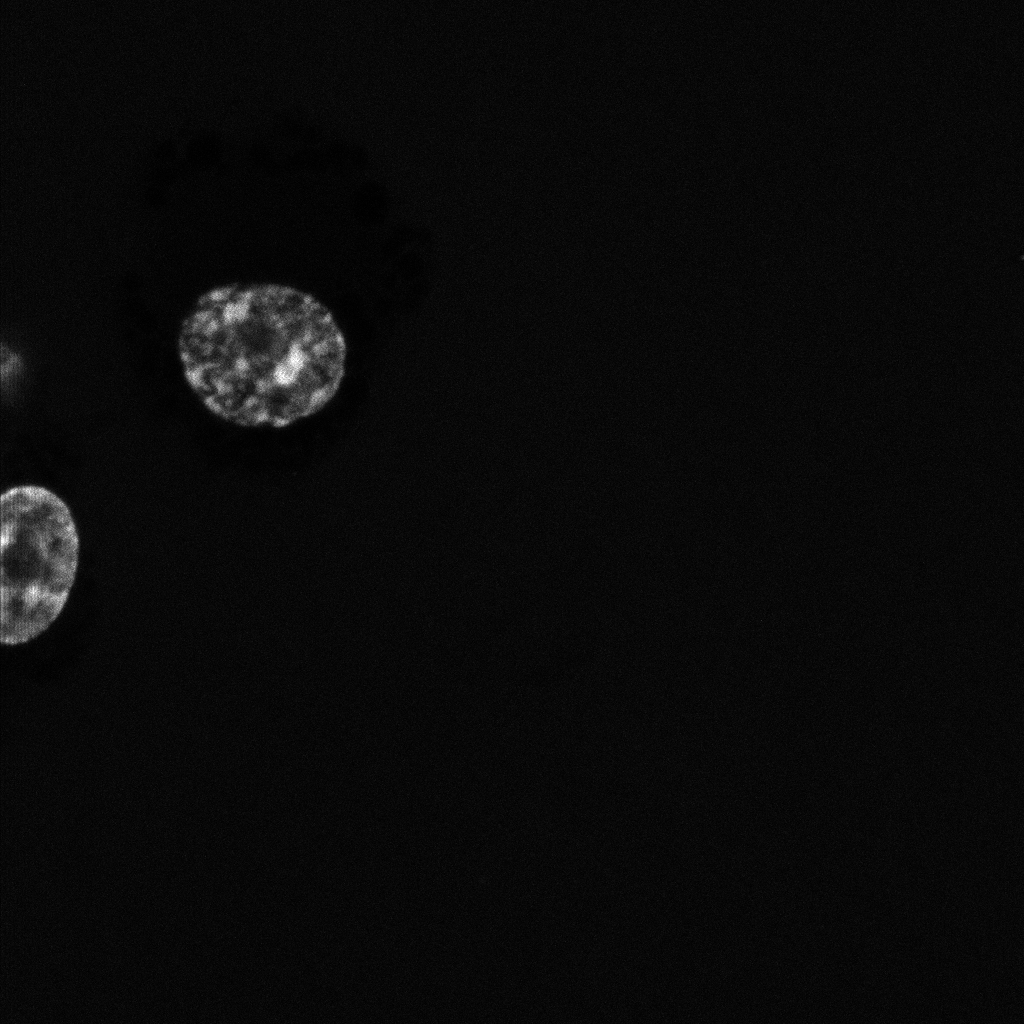

Supplement: Figure 6—source data 2. [file elife-103725-fig6-data2.zip › Figure 6-source data 2/Figure 6A/Infected.tif]

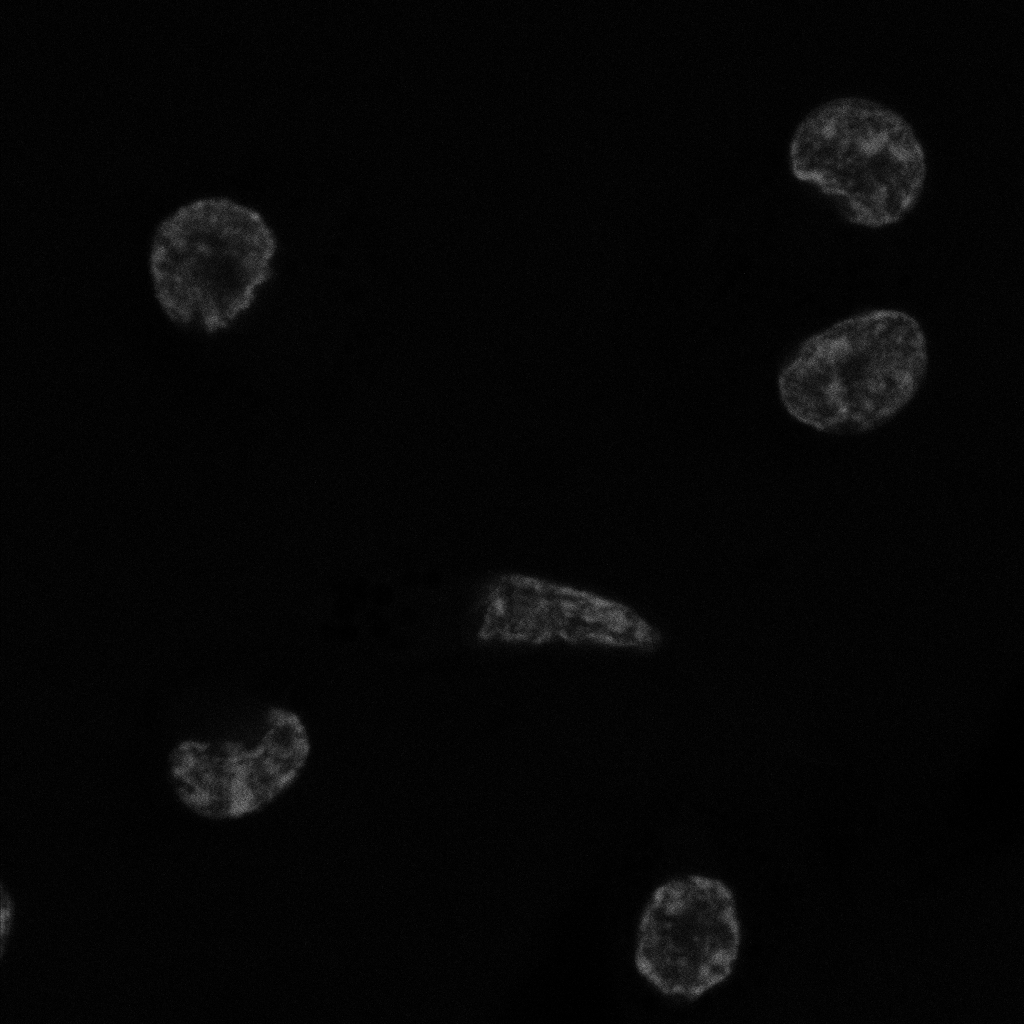

Supplement: Figure 6—source data 2. [file elife-103725-fig6-data2.zip › Figure 6-source data 2/Figure 6A/Non infected.tif]

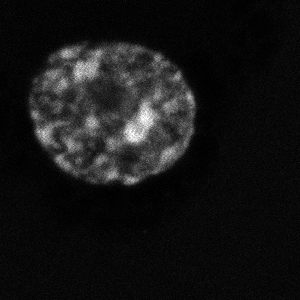

Supplement: Figure 6—source data 3. [file elife-103725-fig6-data3.zip › Figure 6-source data 3/Figure 6A-crops/Inf.tif]

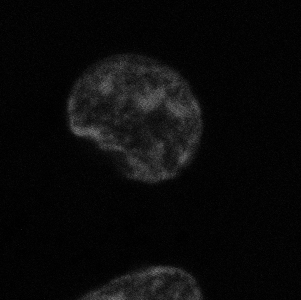

Supplement: Figure 6—source data 3. [file elife-103725-fig6-data3.zip › Figure 6-source data 3/Figure 6A-crops/NonInf.tif]

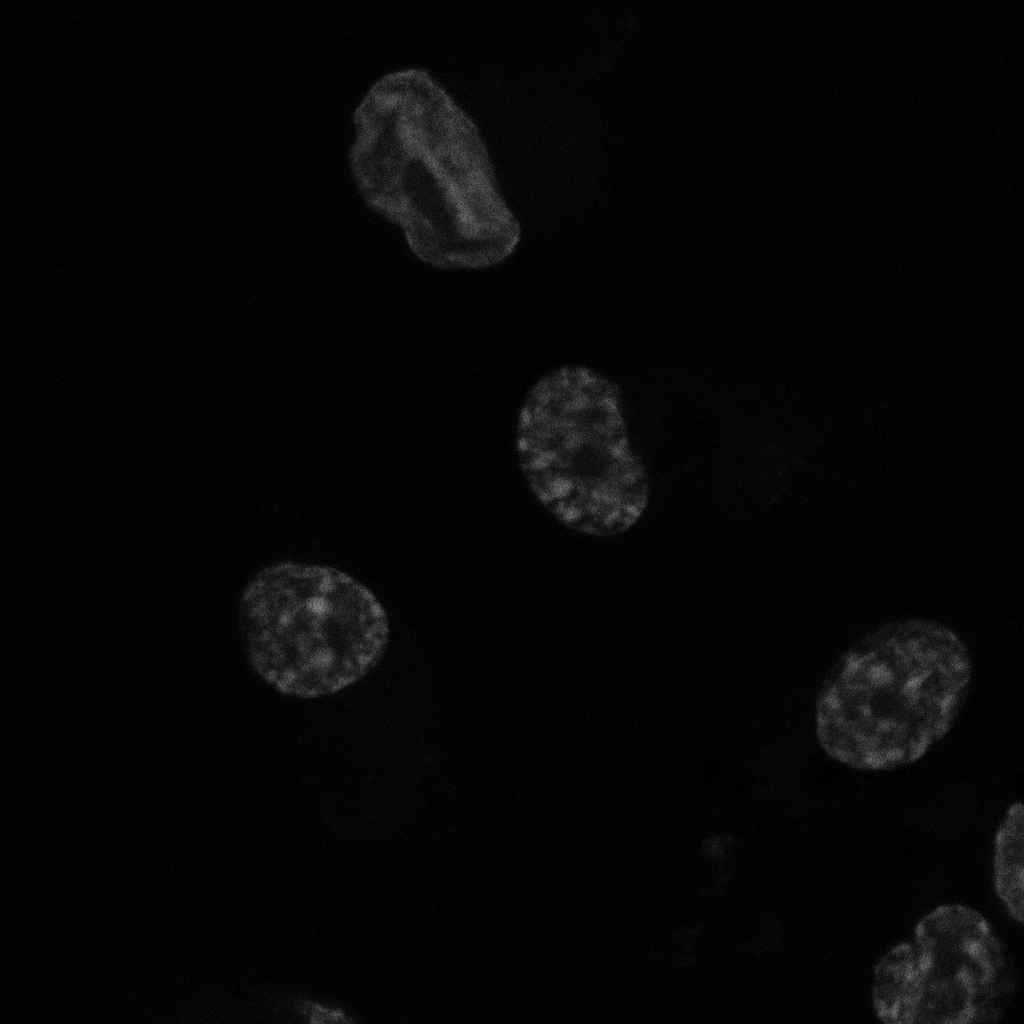

Supplement: Figure 6—source data 4. [file elife-103725-fig6-data4.zip › Figure 6-source data 4/Figure 6B/Inf_CPSF6 DeltaLCR.tif]

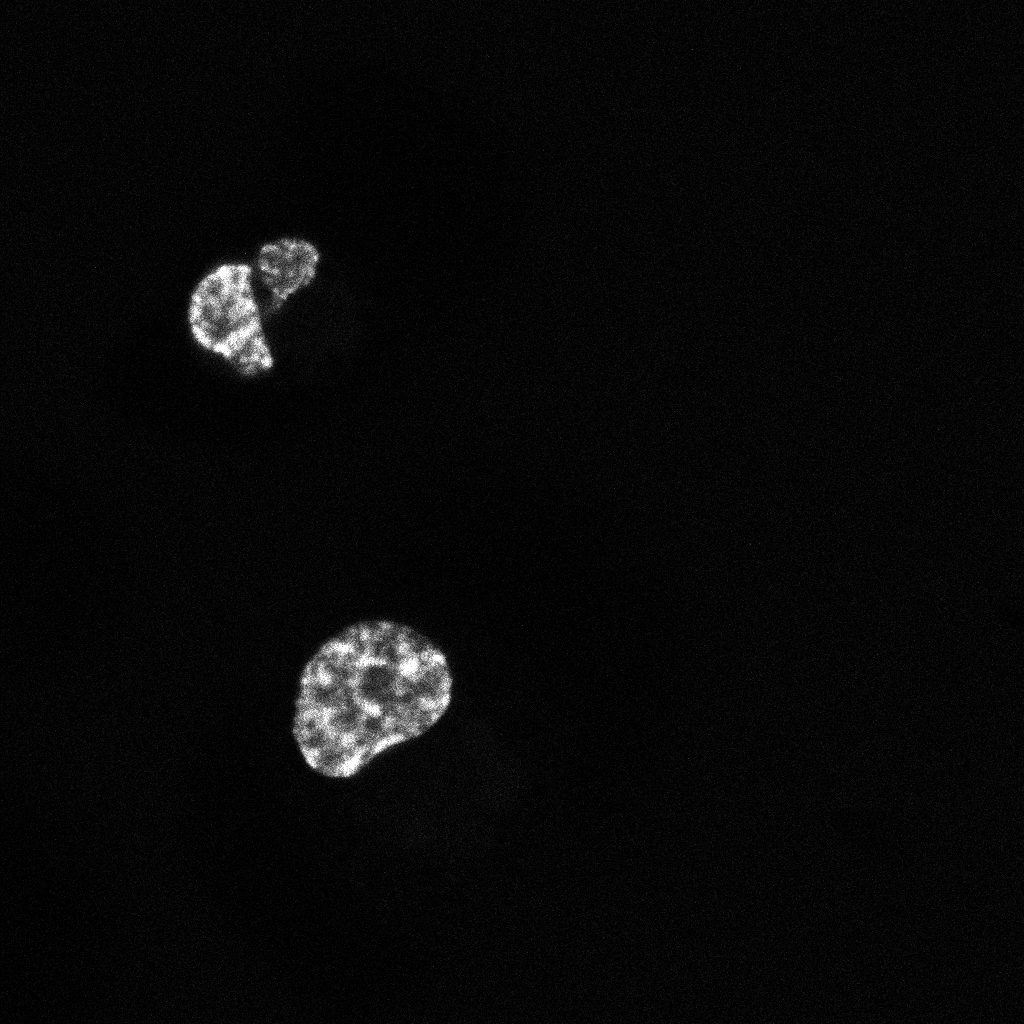

Supplement: Figure 6—source data 4. [file elife-103725-fig6-data4.zip › Figure 6-source data 4/Figure 6B/Inf_CPSF6 DeltaMCD 3xNLS.tif]

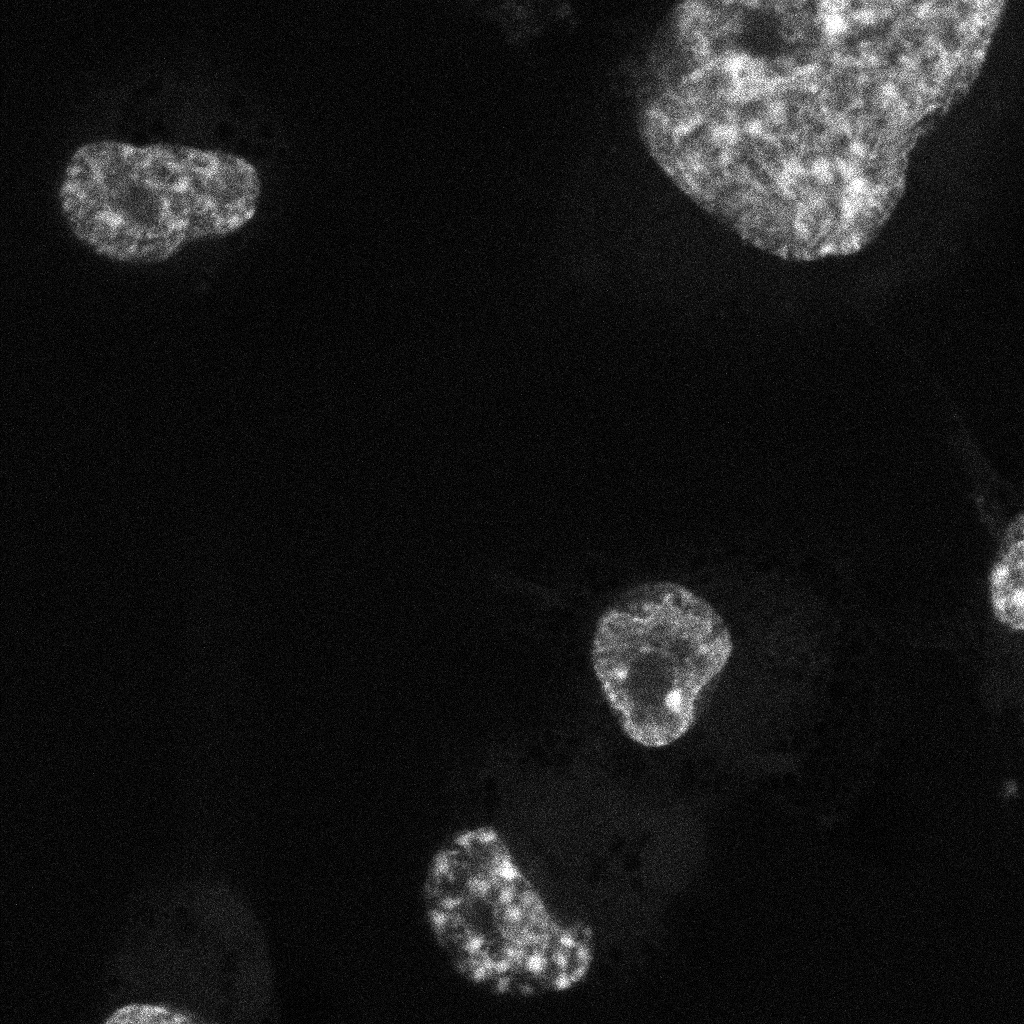

Supplement: Figure 6—source data 4. [file elife-103725-fig6-data4.zip › Figure 6-source data 4/Figure 6B/Inf_CPSF6 DeltaMCD PYNLS.tif]

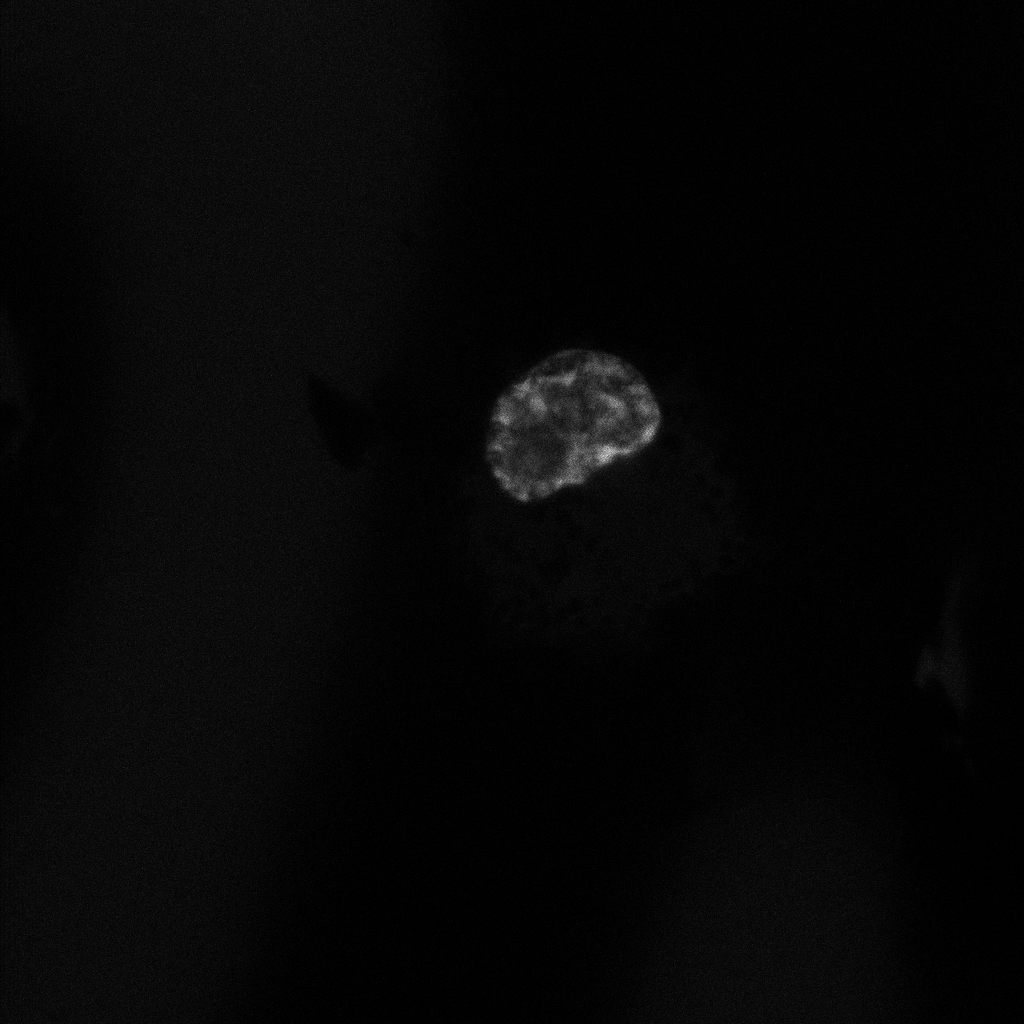

Supplement: Figure 6—source data 4. [file elife-103725-fig6-data4.zip › Figure 6-source data 4/Figure 6B/Inf_CPSF6 DeltaMCD.tif]

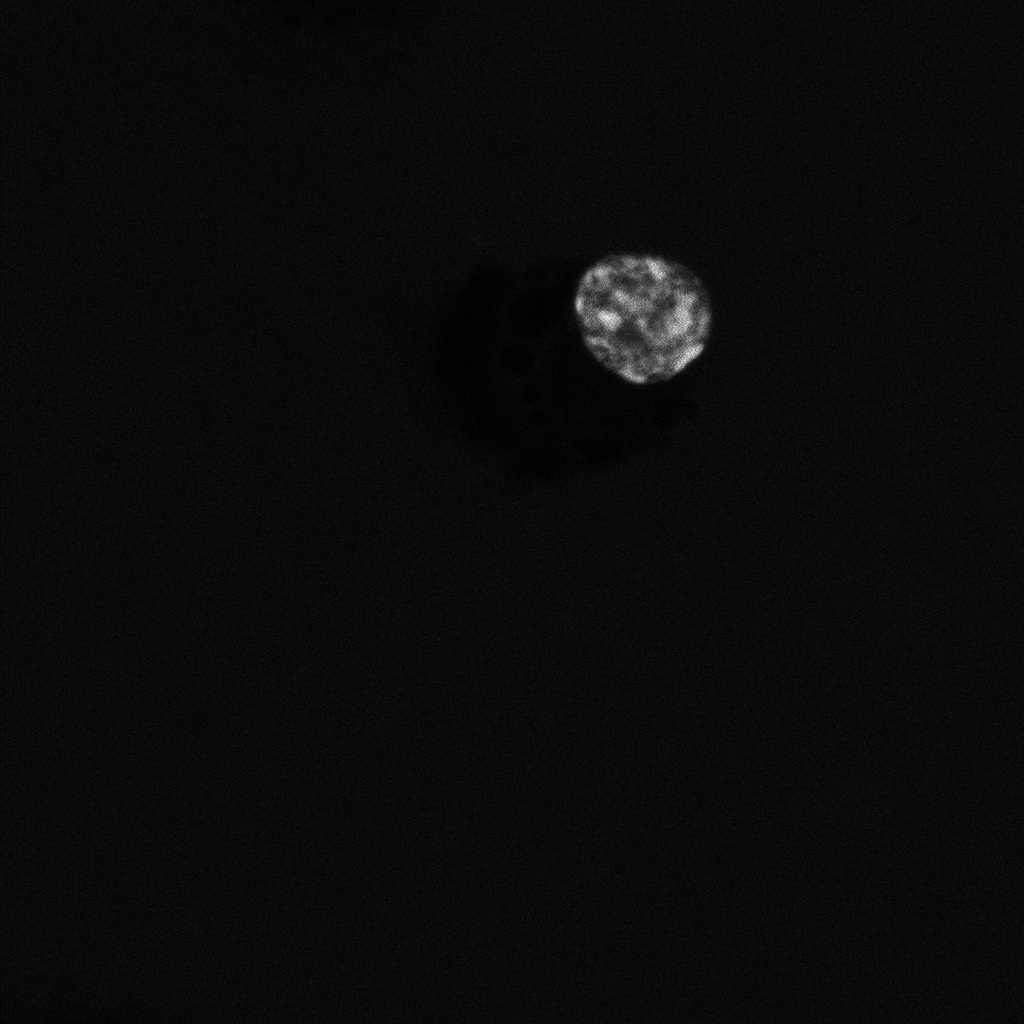

Supplement: Figure 6—source data 4. [file elife-103725-fig6-data4.zip › Figure 6-source data 4/Figure 6B/Inf_CPSF6 WT.tif]

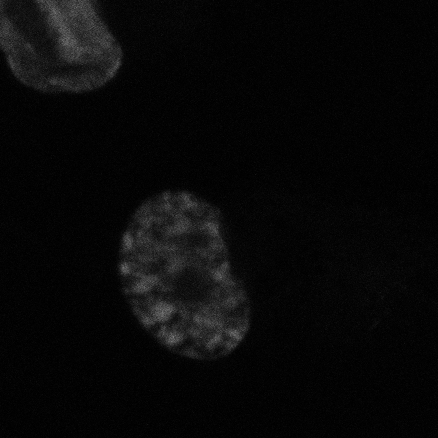

Supplement: Figure 6—source data 5. [file elife-103725-fig6-data5.zip › Figure 6-source data 5/Figure 6B-crops/Inf_CPSF6 DeltaLCR-crop and scale.tif]

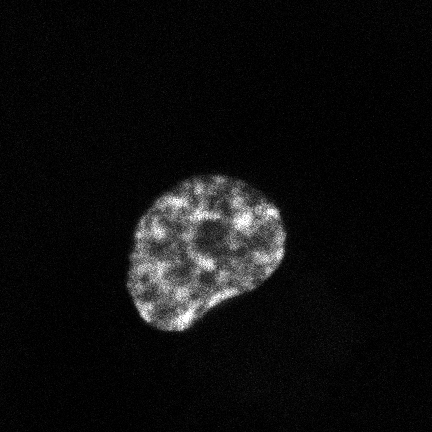

Supplement: Figure 6—source data 5. [file elife-103725-fig6-data5.zip › Figure 6-source data 5/Figure 6B-crops/Inf_CPSF6 DeltaMCD 3xNLS-crop and scale.tif]

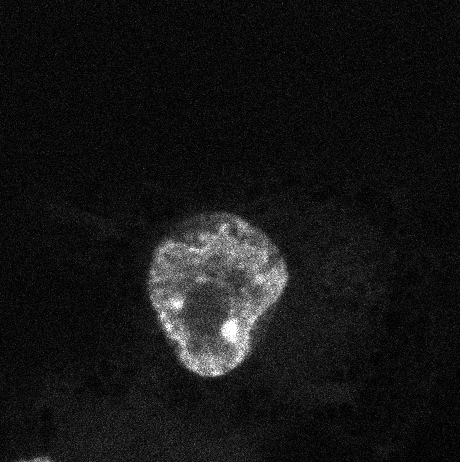

Supplement: Figure 6—source data 5. [file elife-103725-fig6-data5.zip › Figure 6-source data 5/Figure 6B-crops/Inf_CPSF6 DeltaMCD PYNLS-crop and scale.tif]

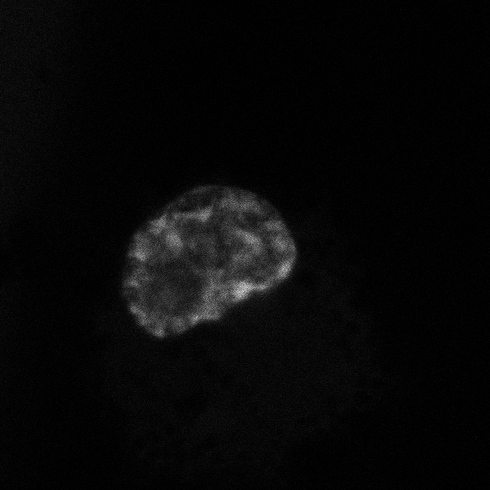

Supplement: Figure 6—source data 5. [file elife-103725-fig6-data5.zip › Figure 6-source data 5/Figure 6B-crops/Inf_CPSF6 DeltaMCD-crop and scale.tif]

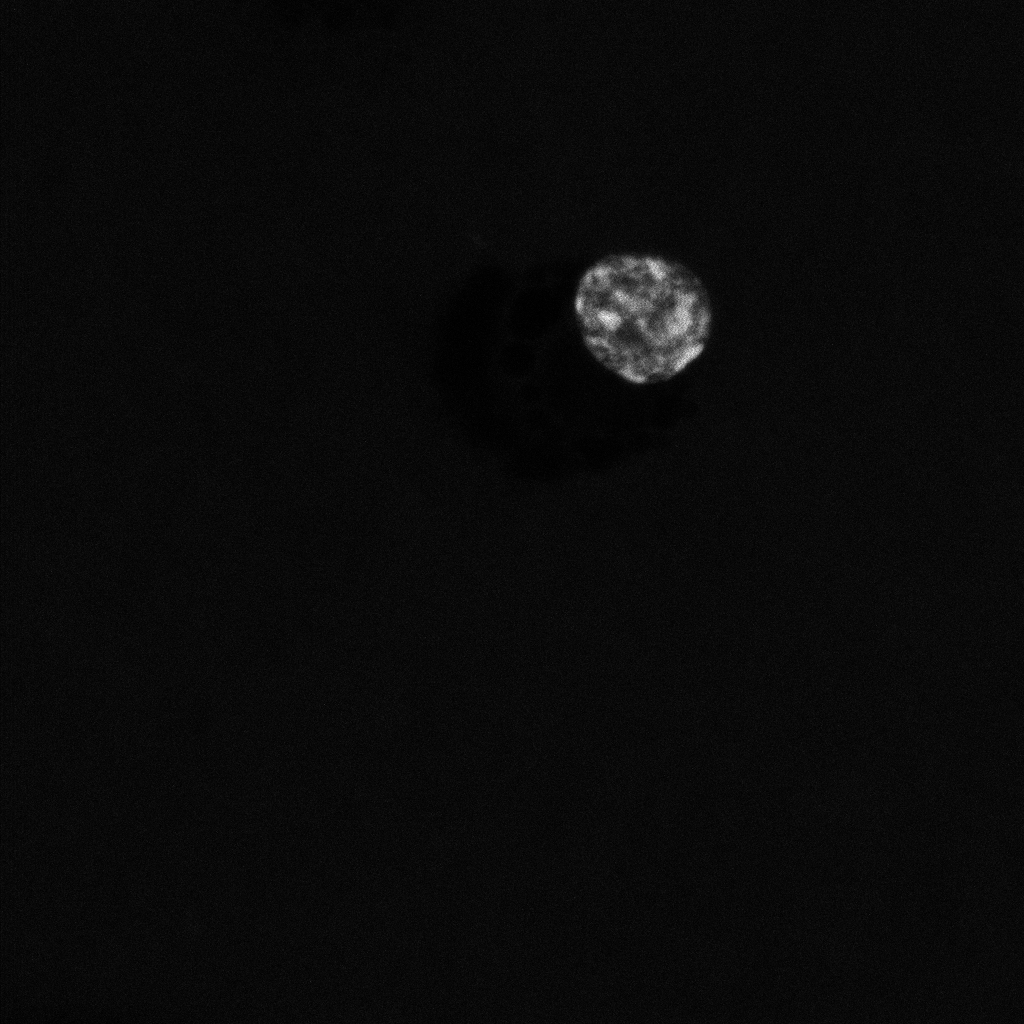

Supplement: Figure 6—source data 5. [file elife-103725-fig6-data5.zip › Figure 6-source data 5/Figure 6B-crops/Inf_CPSF6 WT-crop and scale.tif]

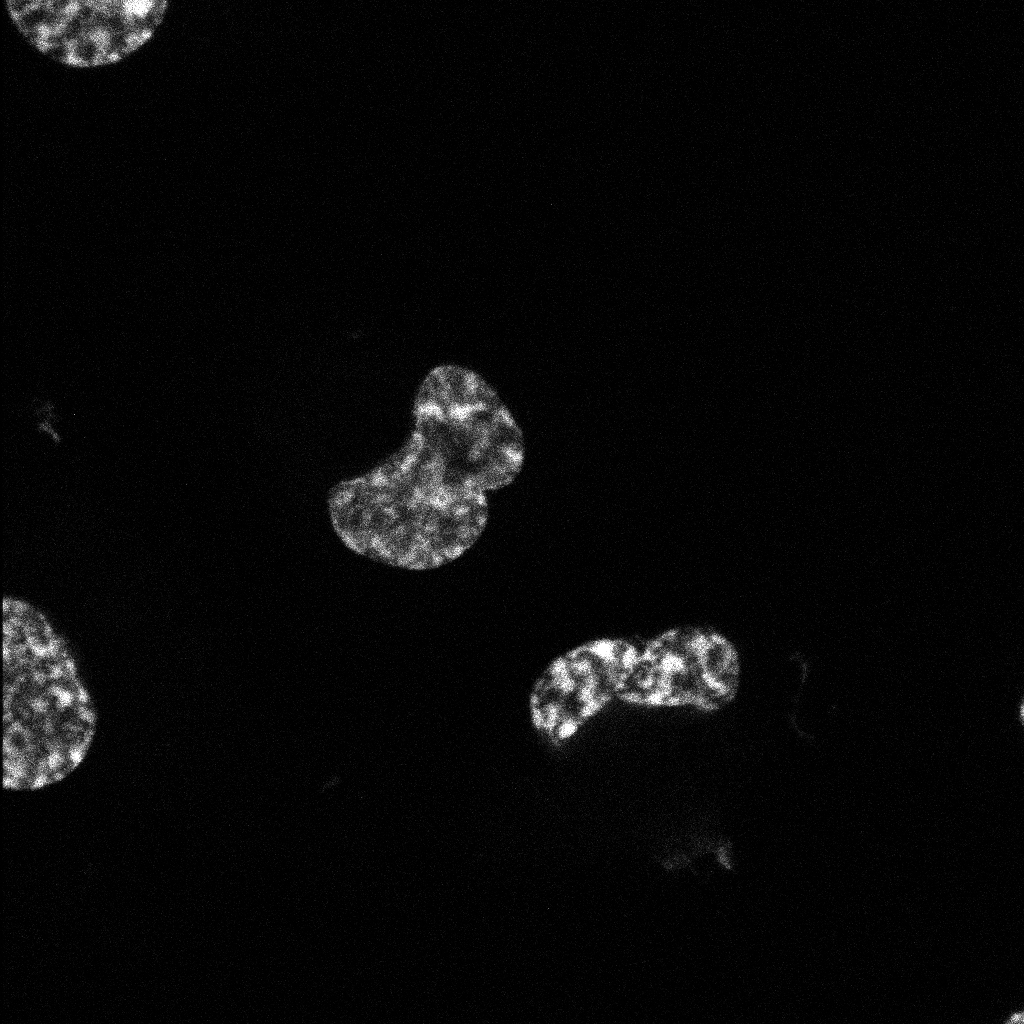

Supplement: Figure 7—source data 2. [file elife-103725-fig7-data2.zip › Figure 7-source data 2/Figure 7A-source data 2/12hpi.tif]

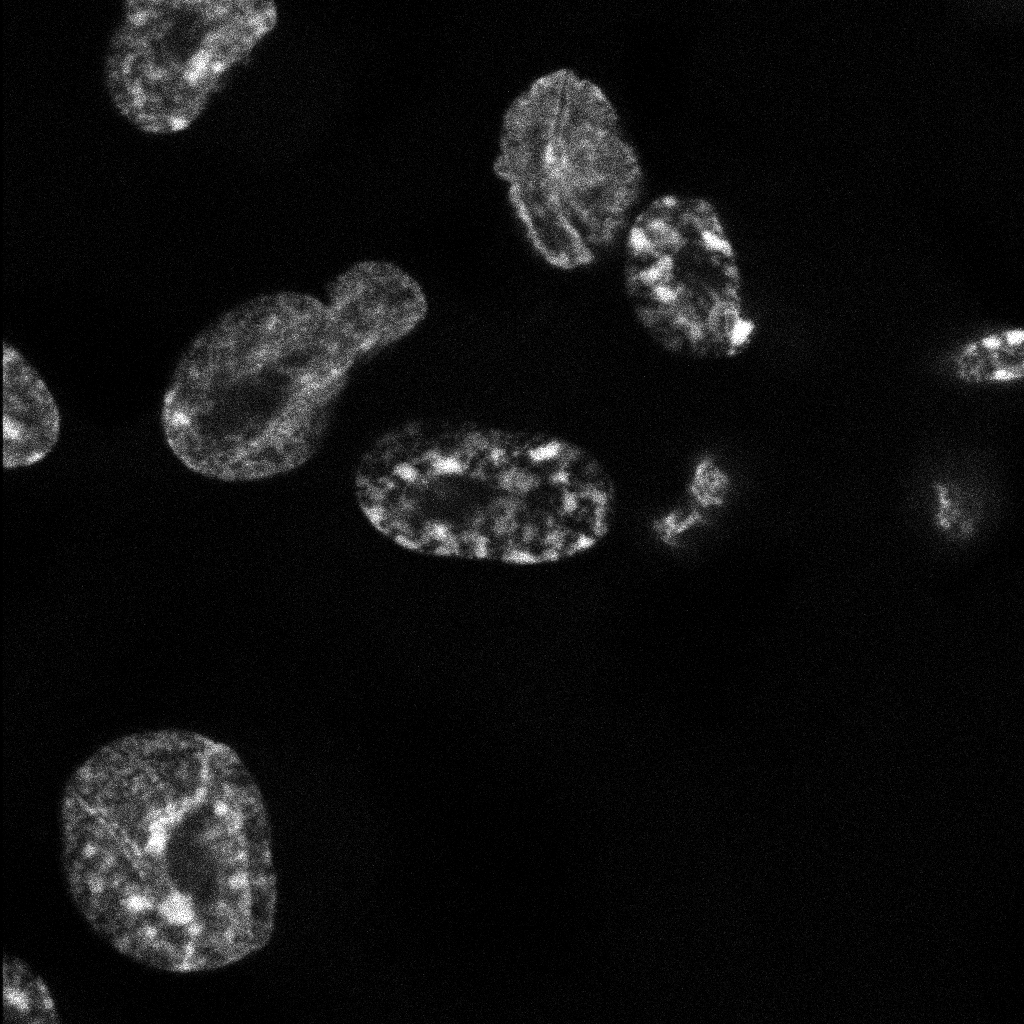

Supplement: Figure 7—source data 2. [file elife-103725-fig7-data2.zip › Figure 7-source data 2/Figure 7A-source data 2/30hpi.tif]

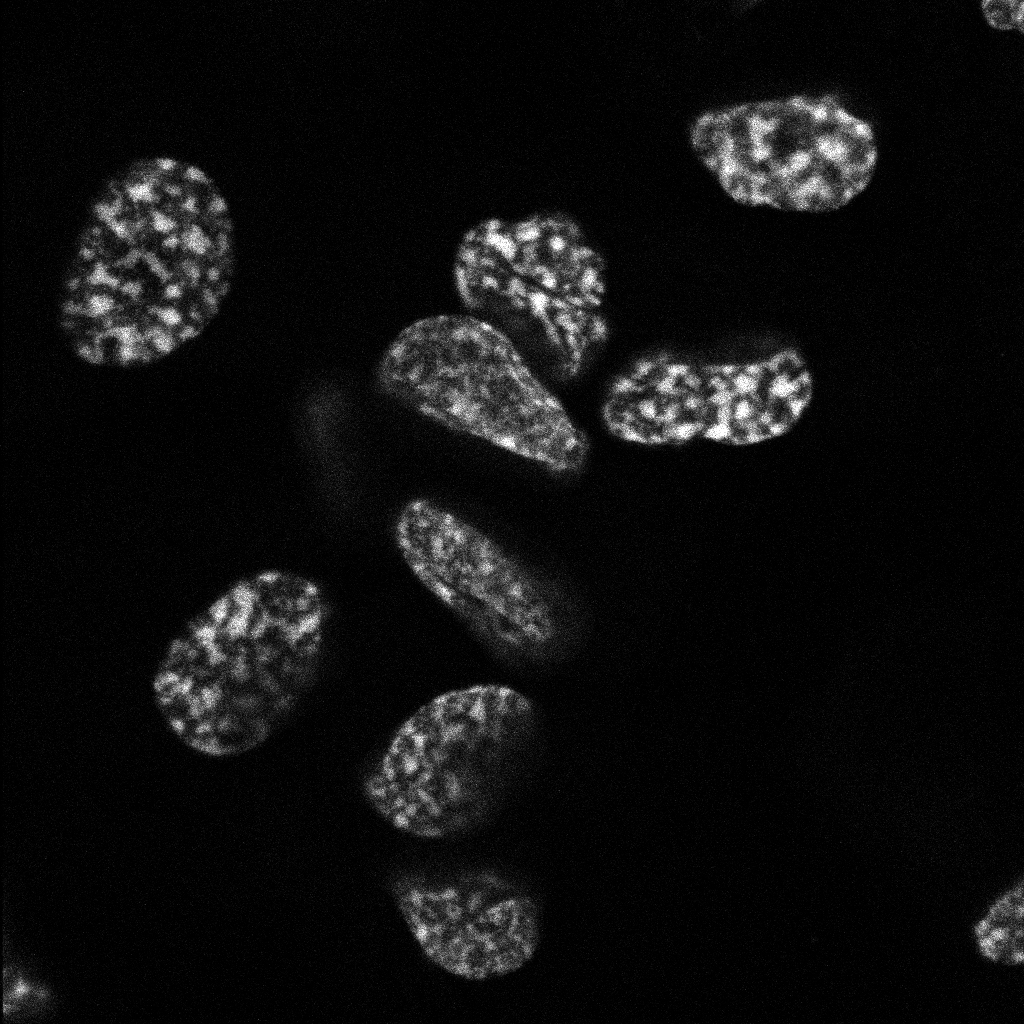

Supplement: Figure 7—source data 2. [file elife-103725-fig7-data2.zip › Figure 7-source data 2/Figure 7A-source data 2/6hpi.tif]

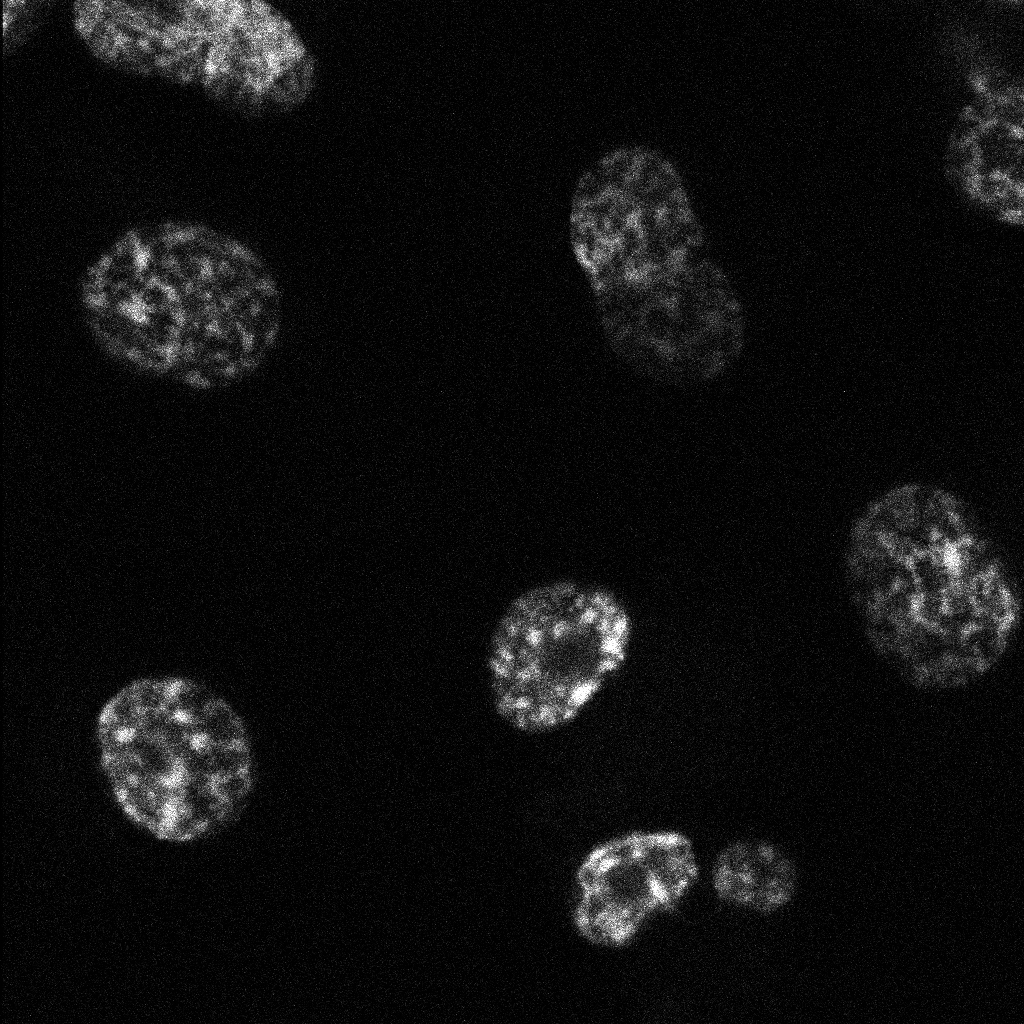

Supplement: Figure 7—source data 2. [file elife-103725-fig7-data2.zip › Figure 7-source data 2/Figure 7A-source data 2/9hpi.tif]

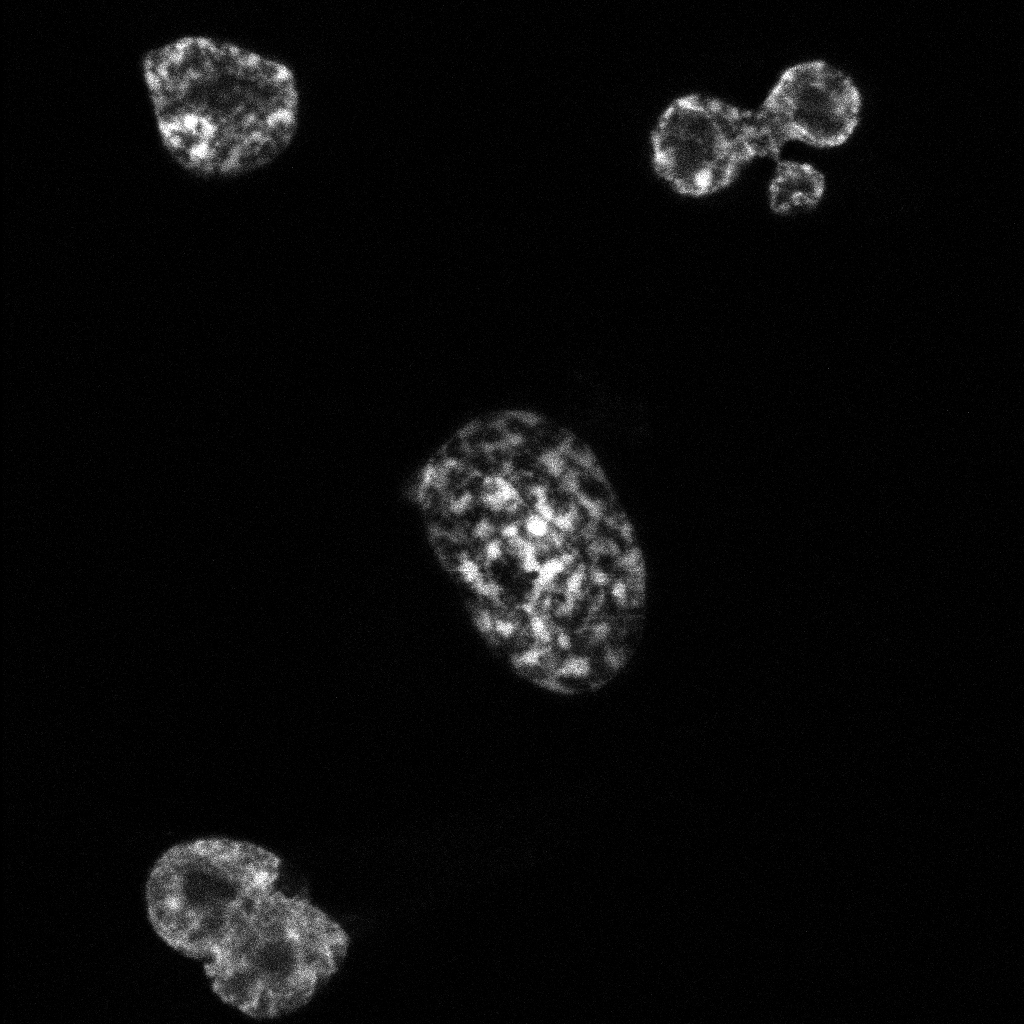

Supplement: Figure 7—source data 2. [file elife-103725-fig7-data2.zip › Figure 7-source data 2/Figure 7A-source data 2/Non inf.tif]

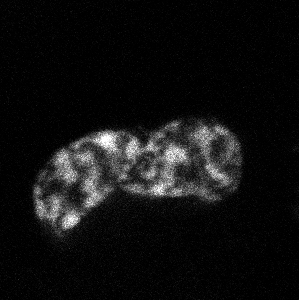

Supplement: Figure 7—source data 3. [file elife-103725-fig7-data3.zip › Figure 7-source data 3/Figure 7A-crops/Figure 7A 12h.tif]

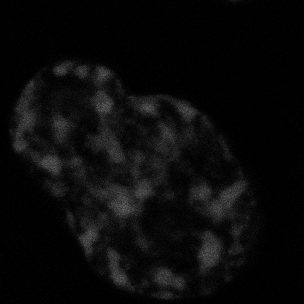

Supplement: Figure 7—source data 3. [file elife-103725-fig7-data3.zip › Figure 7-source data 3/Figure 7A-crops/Figure 7A 30h.tif]

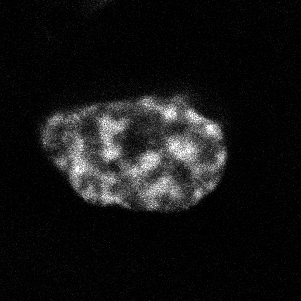

Supplement: Figure 7—source data 3. [file elife-103725-fig7-data3.zip › Figure 7-source data 3/Figure 7A-crops/Figure 7A 6h.tif]

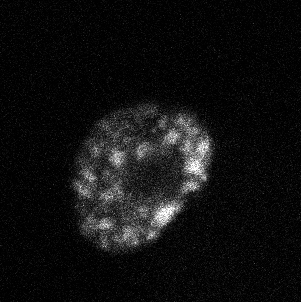

Supplement: Figure 7—source data 3. [file elife-103725-fig7-data3.zip › Figure 7-source data 3/Figure 7A-crops/Figure 7A 9h.tif]

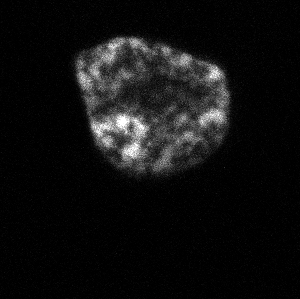

Supplement: Figure 7—source data 3. [file elife-103725-fig7-data3.zip › Figure 7-source data 3/Figure 7A-crops/Figure 7A Non inf.tif]

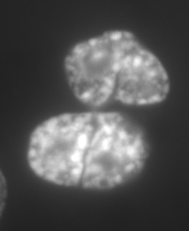

Supplement: Figure 8—source data 2. [file elife-103725-fig8-data2.zip › Figure 8-source data 2/Figure 8A/ctrl SON&SRRM2.tif]

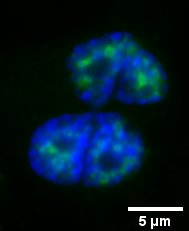

Supplement: Figure 8—source data 2. [file elife-103725-fig8-data2.zip › Figure 8-source data 2/Figure 8A/ctrl SON.jpg]

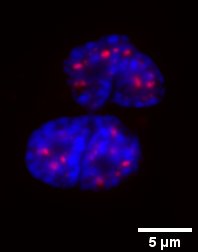

Supplement: Figure 8—source data 2. [file elife-103725-fig8-data2.zip › Figure 8-source data 2/Figure 8A/Ctrl SRRM2.jpg]

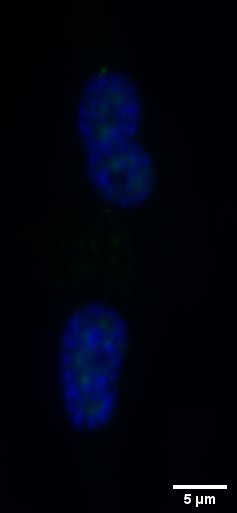

Supplement: Figure 8—source data 2. [file elife-103725-fig8-data2.zip › Figure 8-source data 2/Figure 8A/KD SON_SON.jpg]

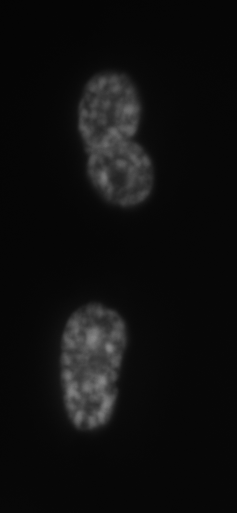

Supplement: Figure 8—source data 2. [file elife-103725-fig8-data2.zip › Figure 8-source data 2/Figure 8A/KD SON_SON.tif]

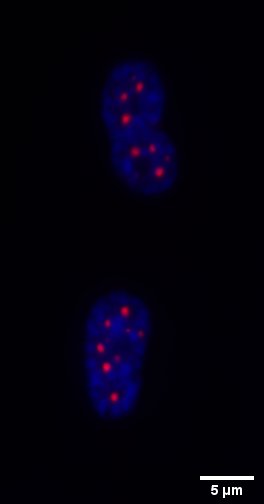

Supplement: Figure 8—source data 2. [file elife-103725-fig8-data2.zip › Figure 8-source data 2/Figure 8A/KD SON_SRRM2.jpg]

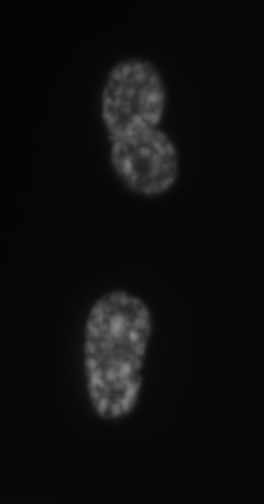

Supplement: Figure 8—source data 2. [file elife-103725-fig8-data2.zip › Figure 8-source data 2/Figure 8A/KD SON_SRRM2.tif]

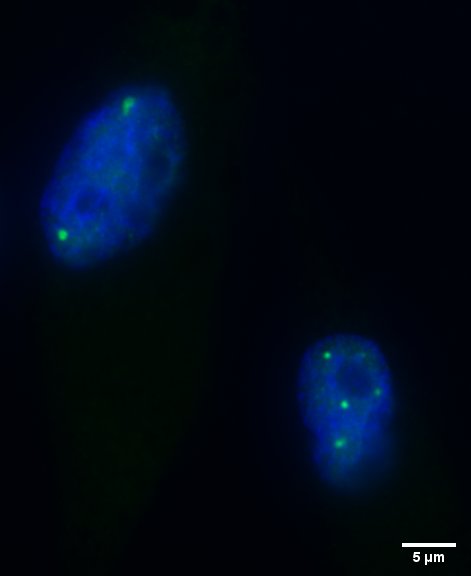

Supplement: Figure 8—source data 2. [file elife-103725-fig8-data2.zip › Figure 8-source data 2/Figure 8A/KD SRRM2_SON.jpg]

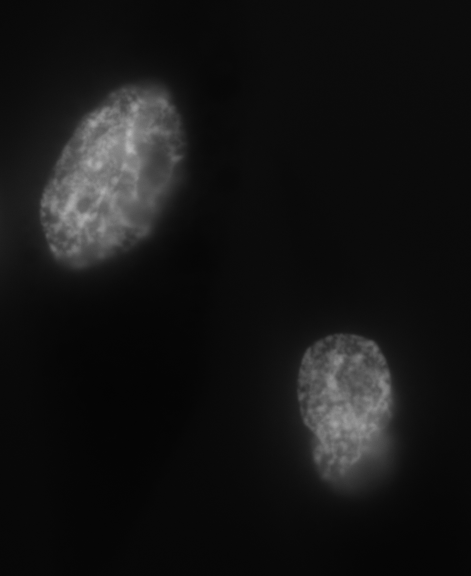

Supplement: Figure 8—source data 2. [file elife-103725-fig8-data2.zip › Figure 8-source data 2/Figure 8A/KD SRRM2_SON.tif]

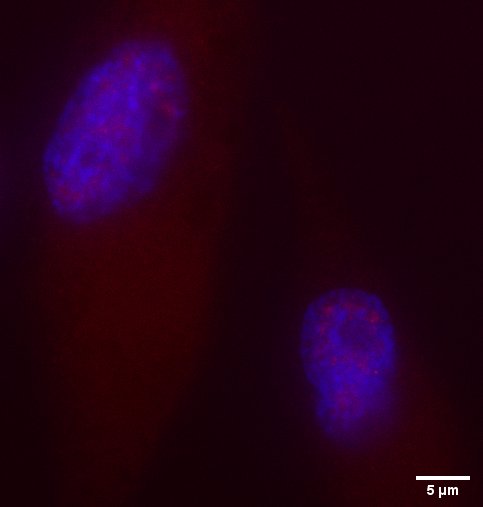

Supplement: Figure 8—source data 2. [file elife-103725-fig8-data2.zip › Figure 8-source data 2/Figure 8A/KD SRRM2_SRRM2.jpg]

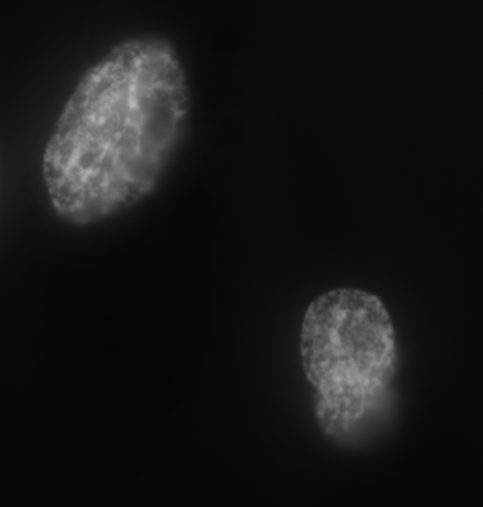

Supplement: Figure 8—source data 2. [file elife-103725-fig8-data2.zip › Figure 8-source data 2/Figure 8A/KD SRRM2_SRRM2.tif]

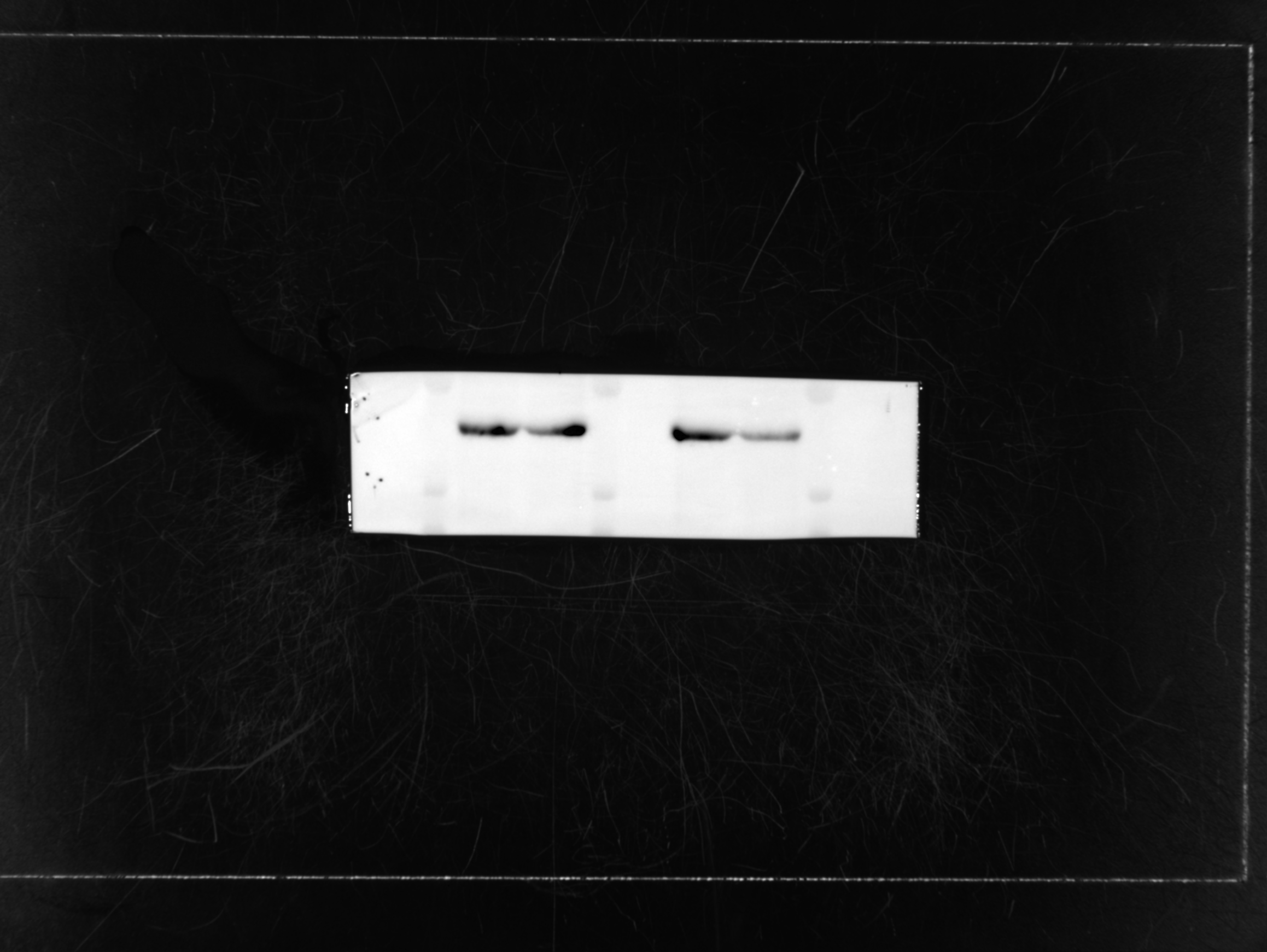

Supplement: Figure 8—source data 3. [file elife-103725-fig8-data3.zip › Figure 8-source data 3/Actin SON gel.tif]

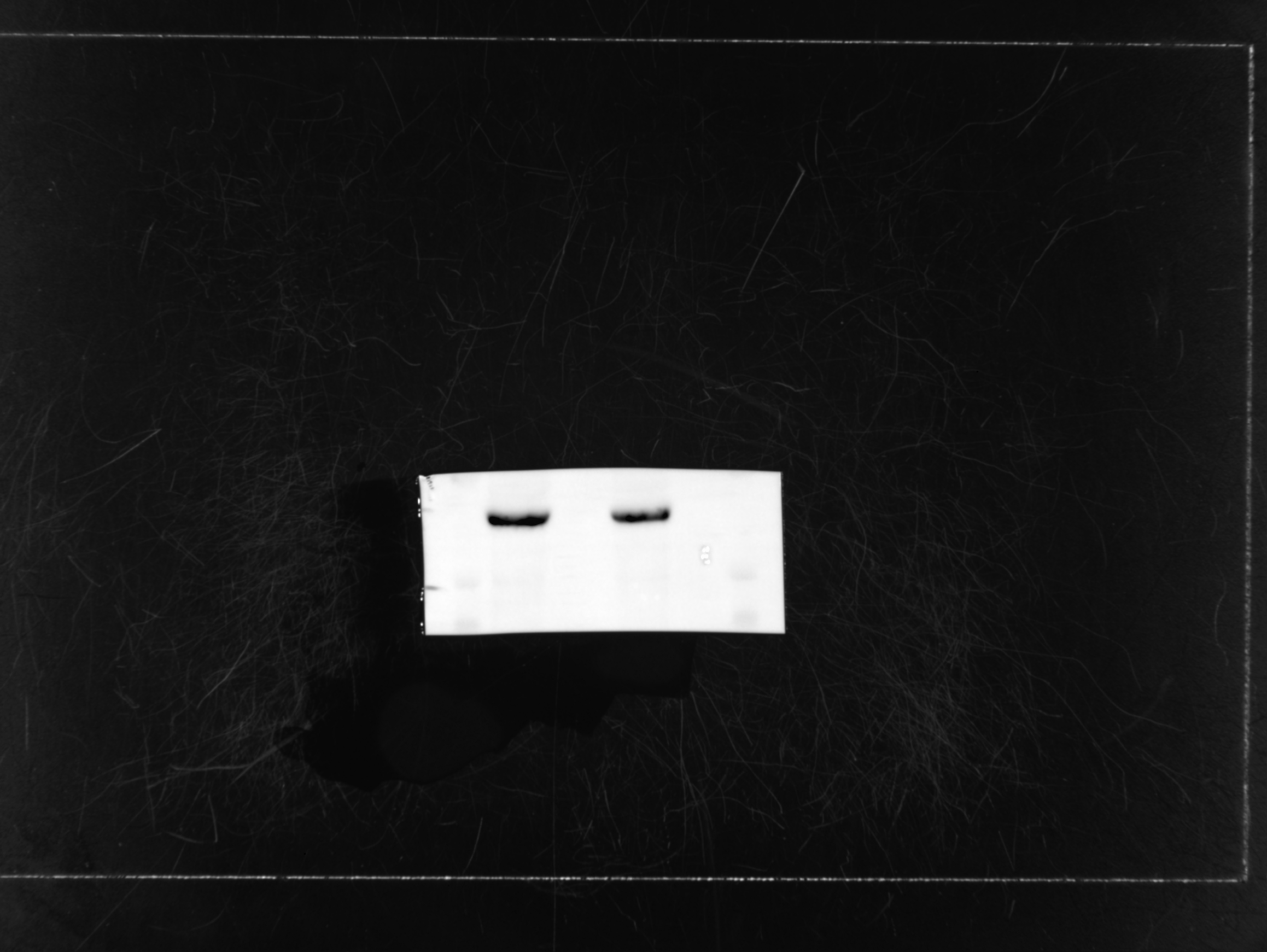

Supplement: Figure 8—source data 3. [file elife-103725-fig8-data3.zip › Figure 8-source data 3/Actin SRRM2 gel.tif]

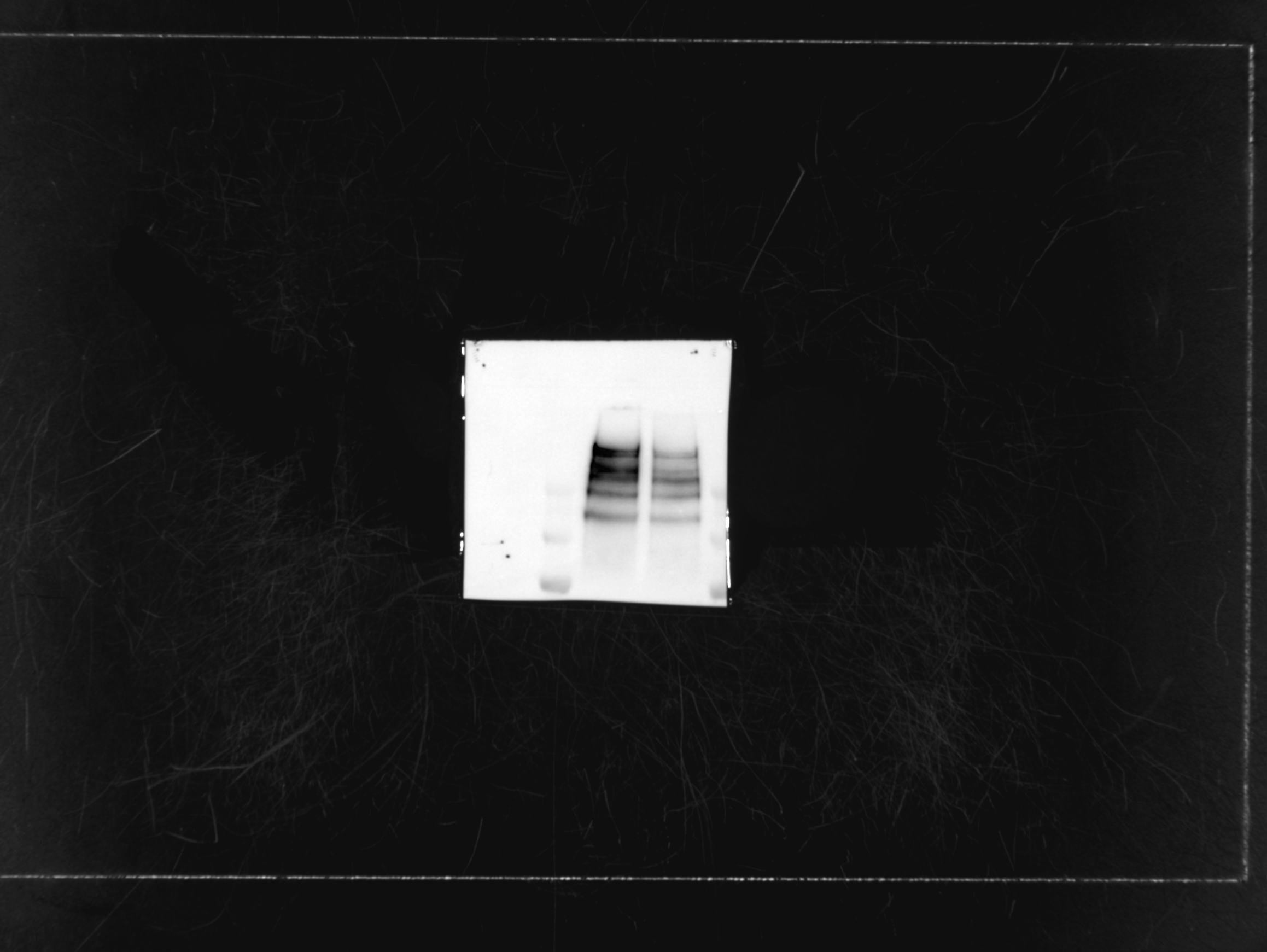

Supplement: Figure 8—source data 3. [file elife-103725-fig8-data3.zip › Figure 8-source data 3/SON.tif]

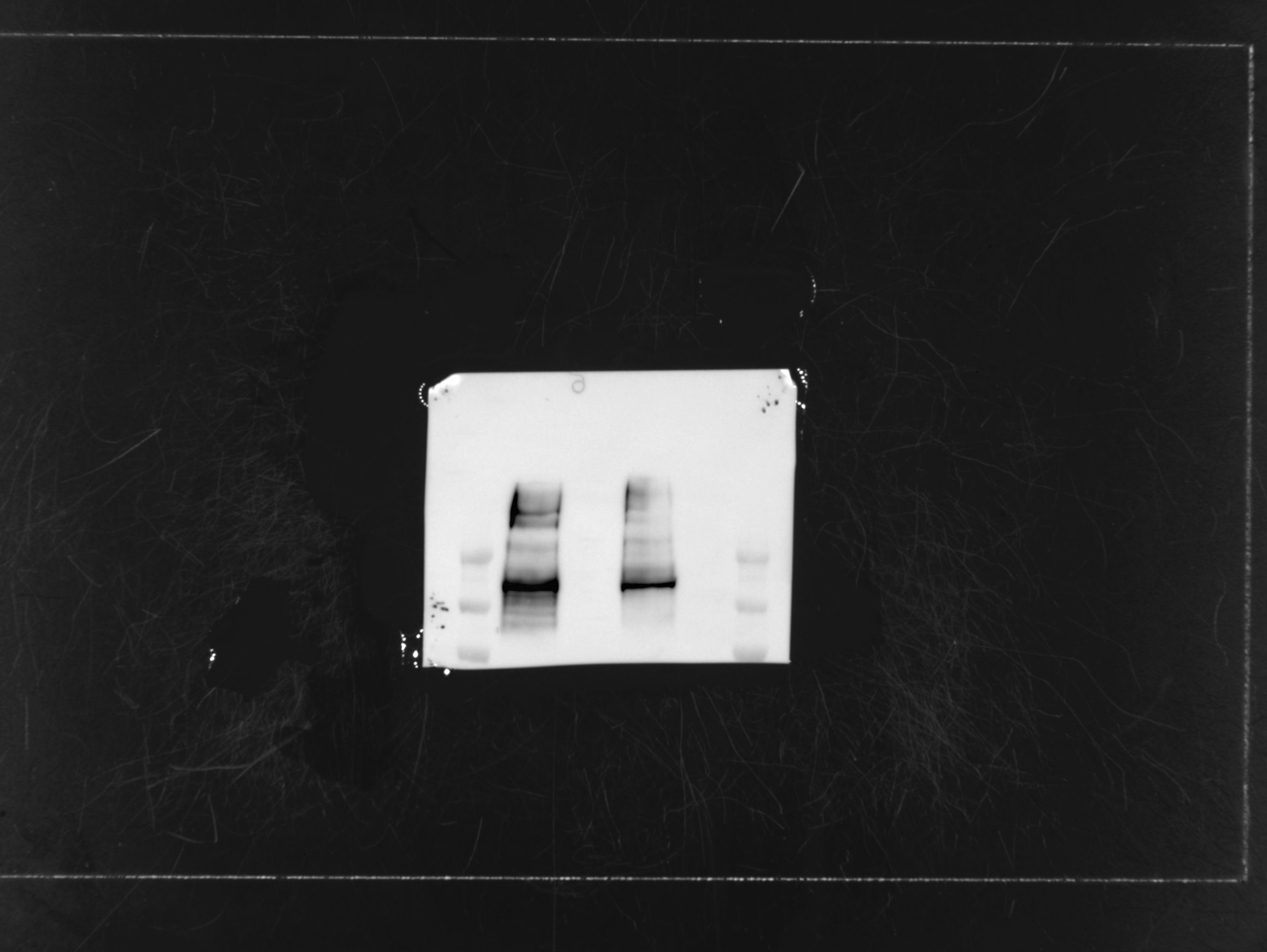

Supplement: Figure 8—source data 3. [file elife-103725-fig8-data3.zip › Figure 8-source data 3/SRRM2.tif]

Actin

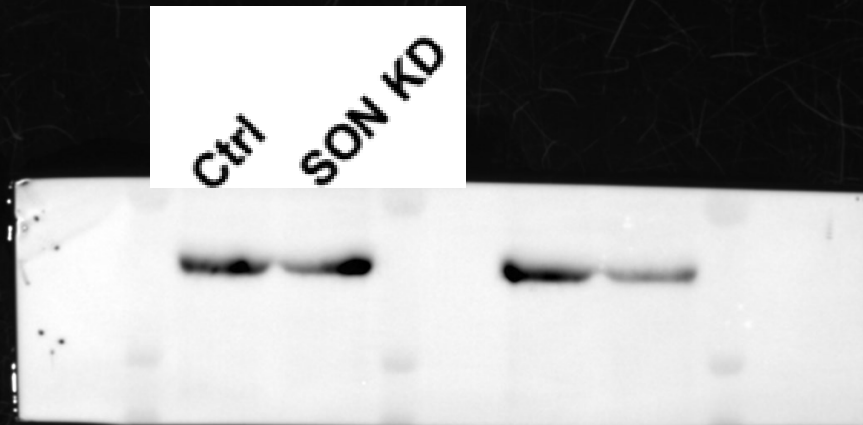

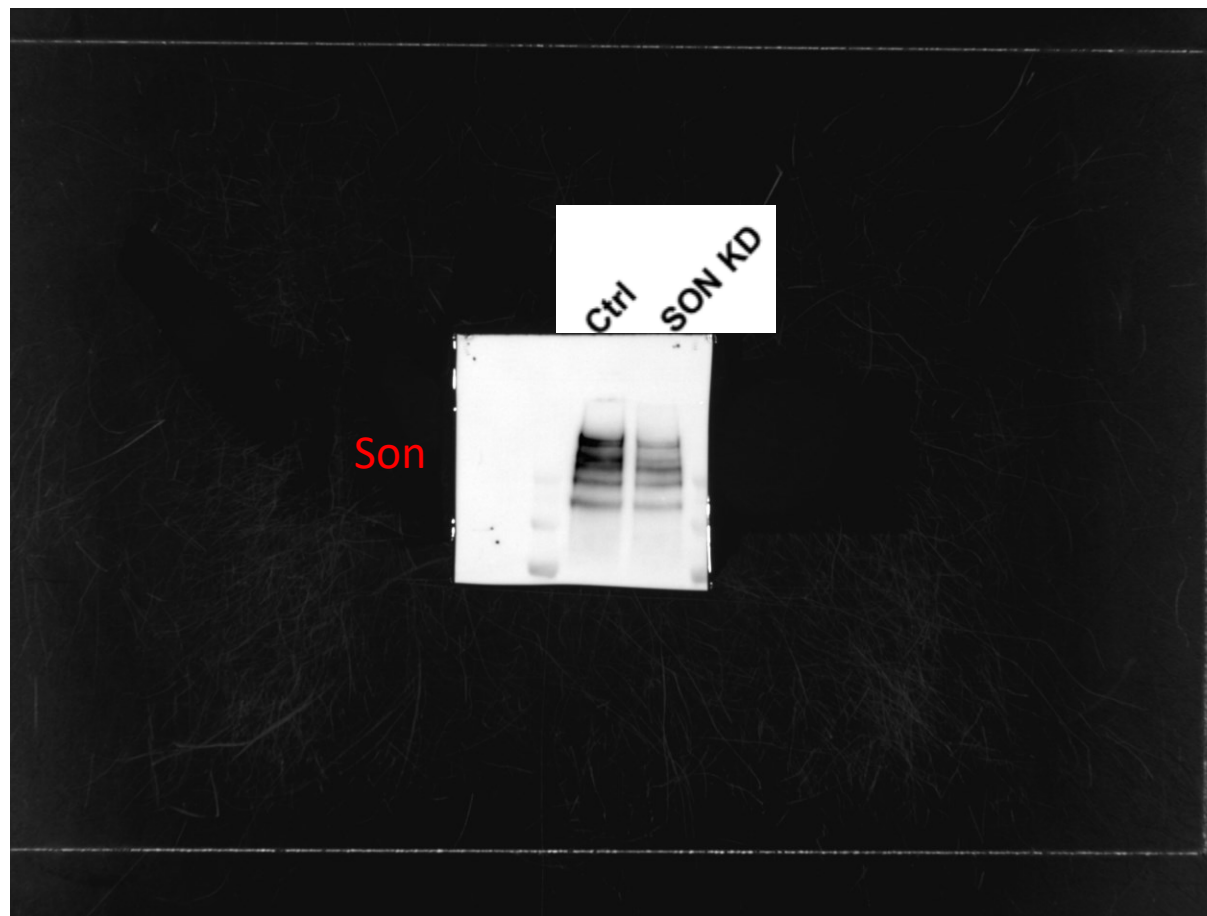

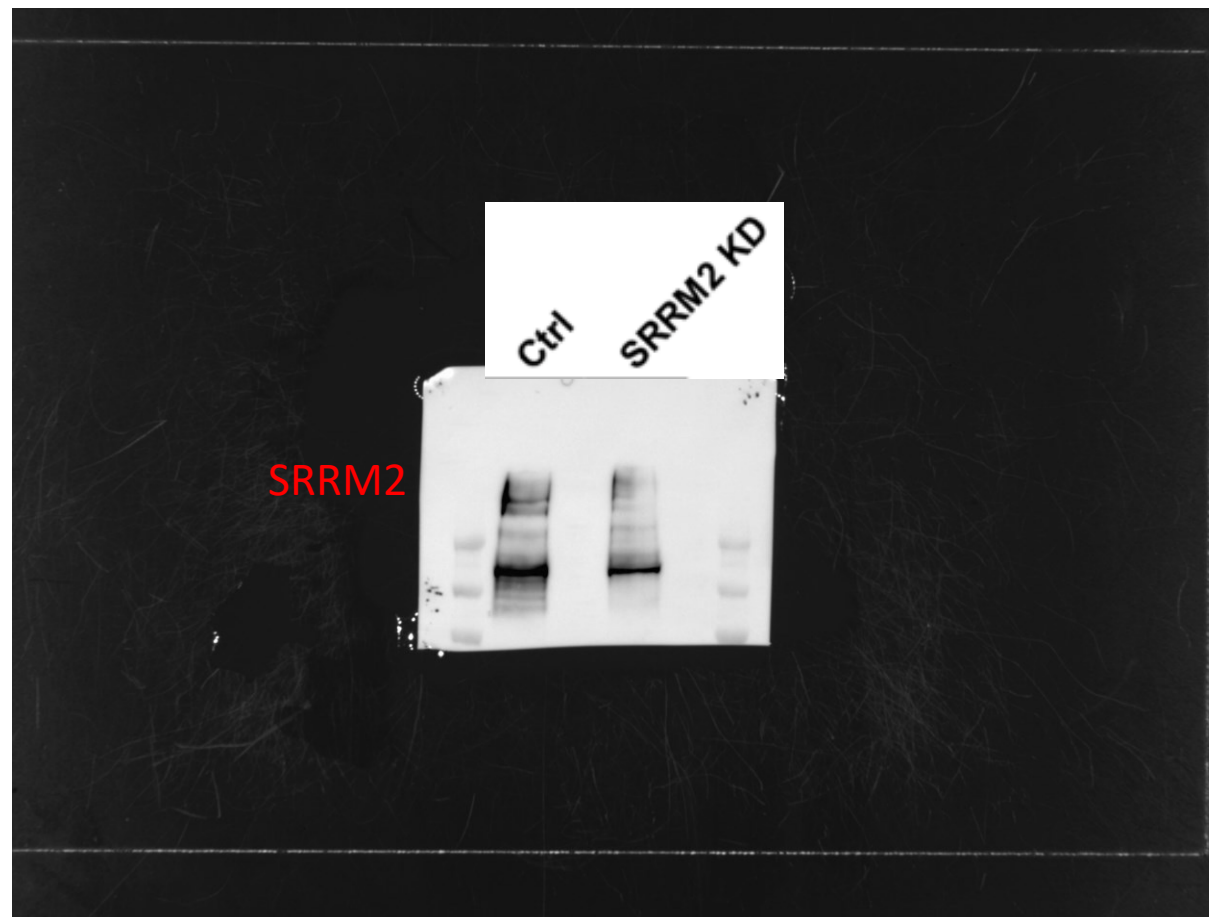

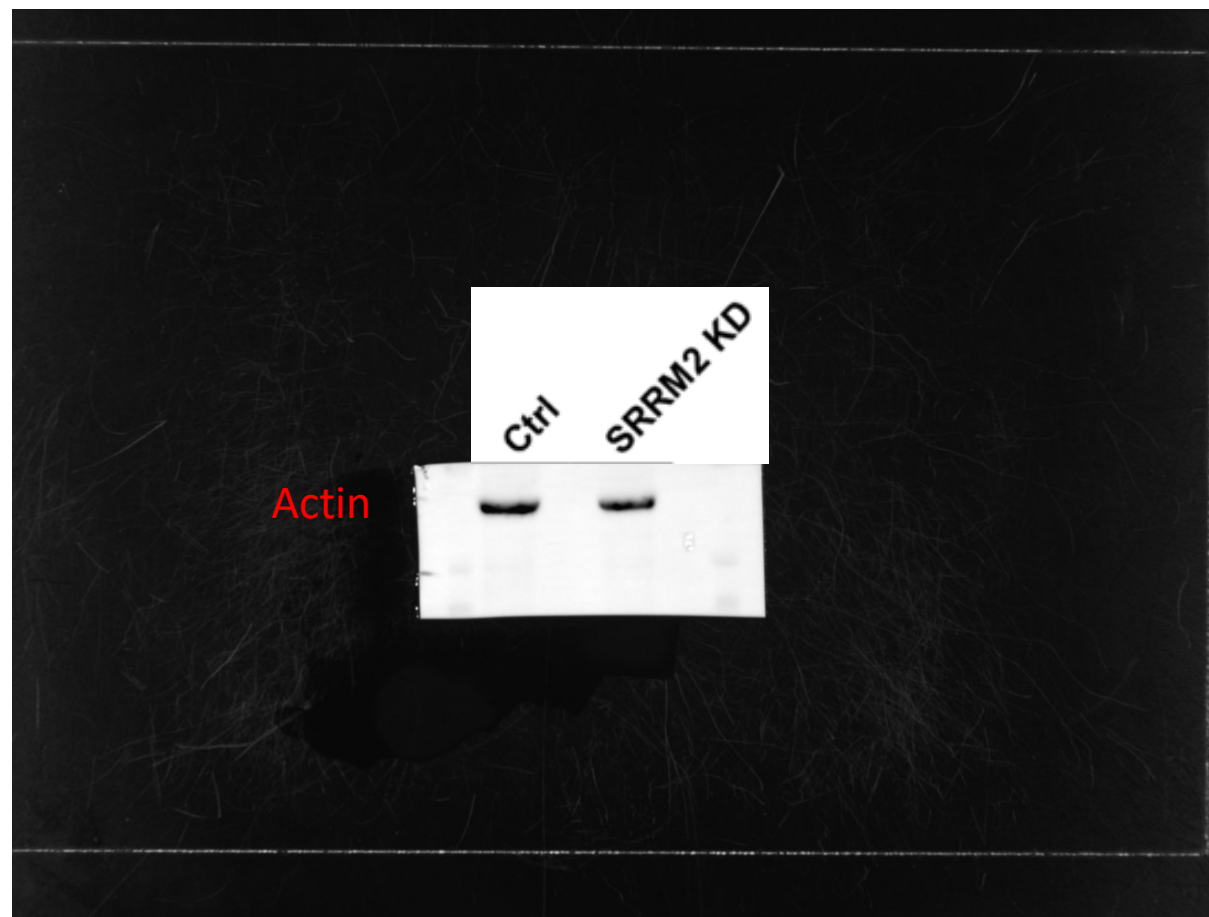

Supplement: Figure 8—source data 4. [file elife-103725-fig8-data4.zip › Figure 8-source data 4/Figure 8A-annotated.pdf]

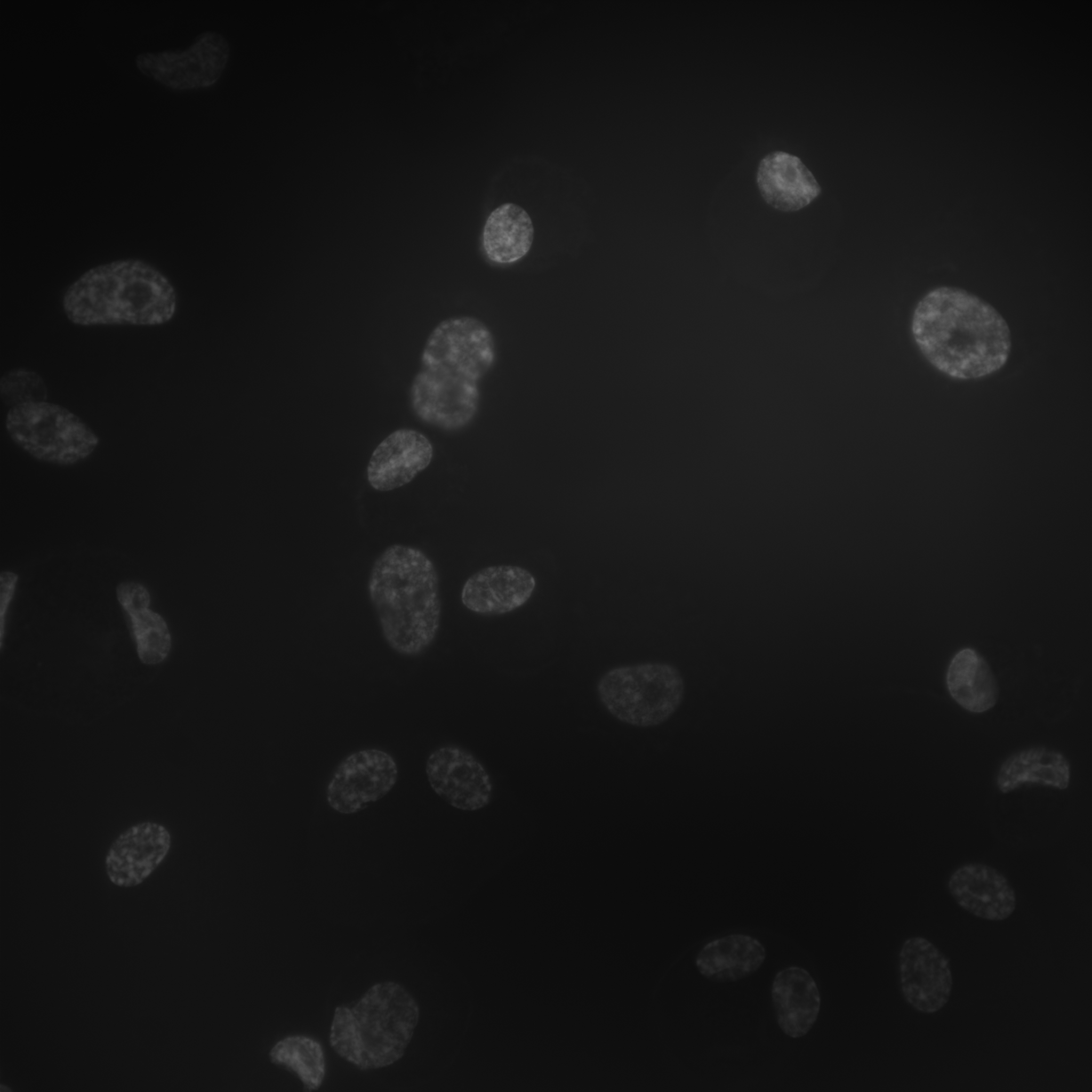

Supplement: Figure 8—source data 5. [file elife-103725-fig8-data5.zip › Figure 8-source data 5/Figure 8B-source data 2/Ctrl 1 CPSF6 clusters.tif]

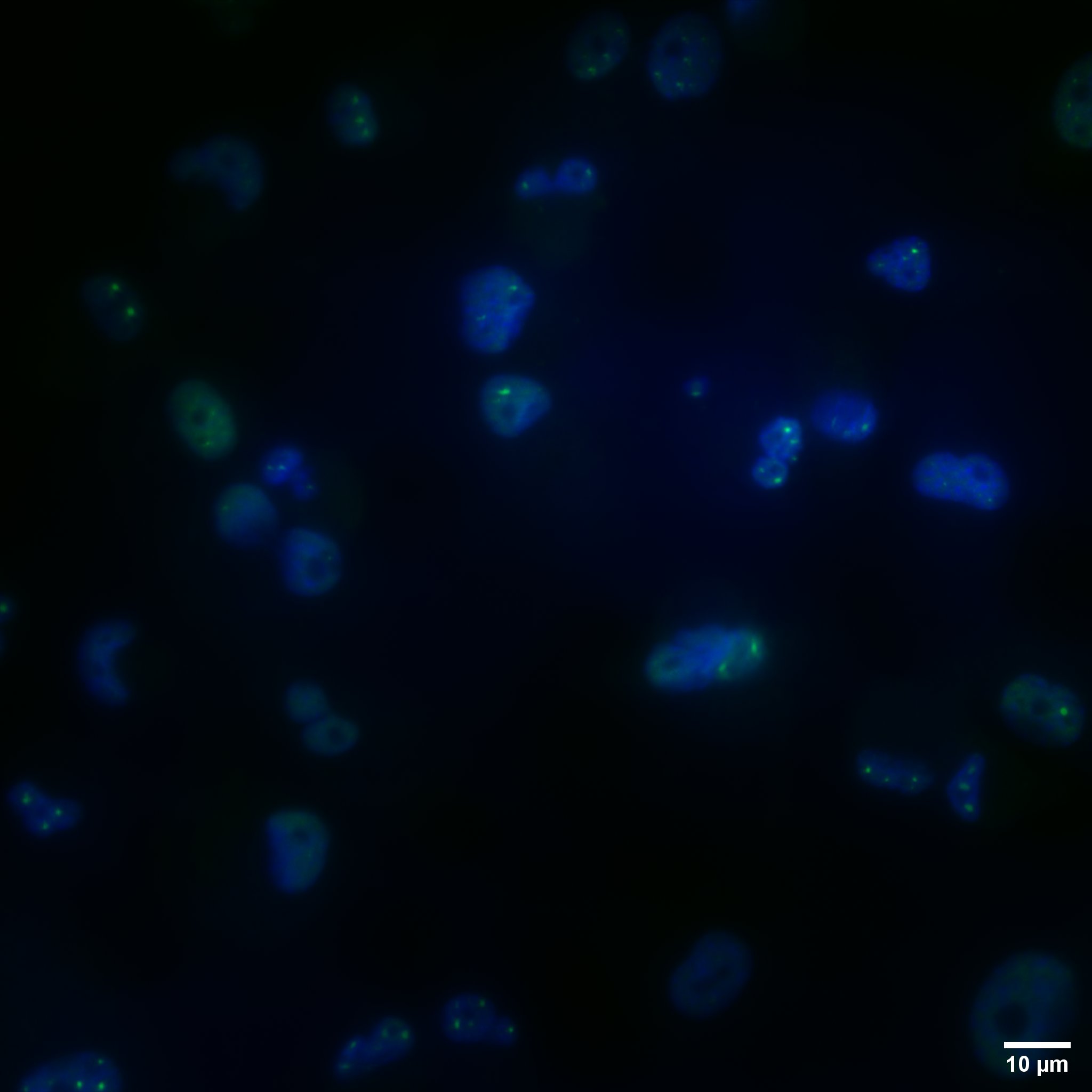

Supplement: Figure 8—source data 5. [file elife-103725-fig8-data5.zip › Figure 8-source data 5/Figure 8B-source data 2/SON -CPSF6 clusters.jpg]

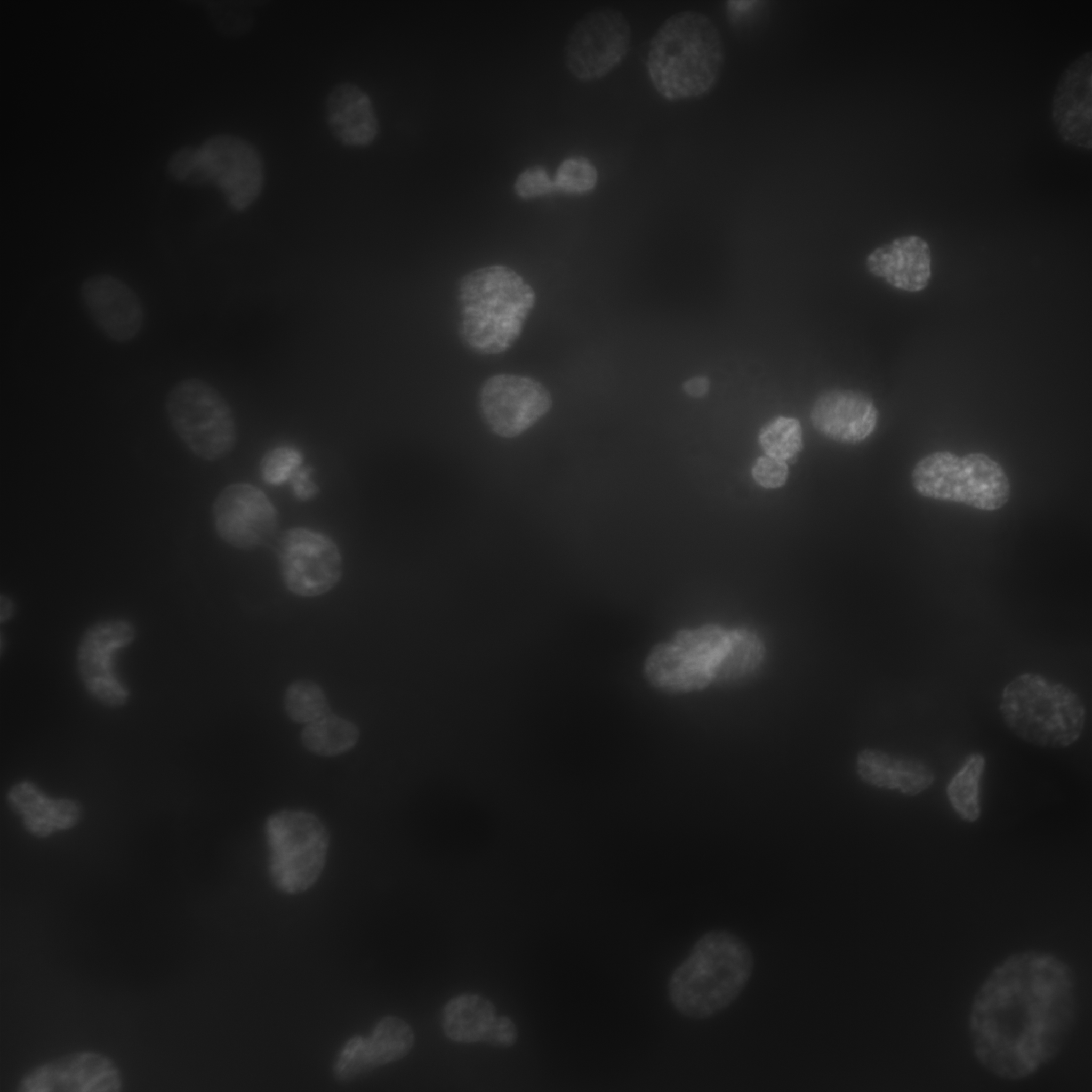

Supplement: Figure 8—source data 5. [file elife-103725-fig8-data5.zip › Figure 8-source data 5/Figure 8B-source data 2/SON -CPSF6 clusters.tif]

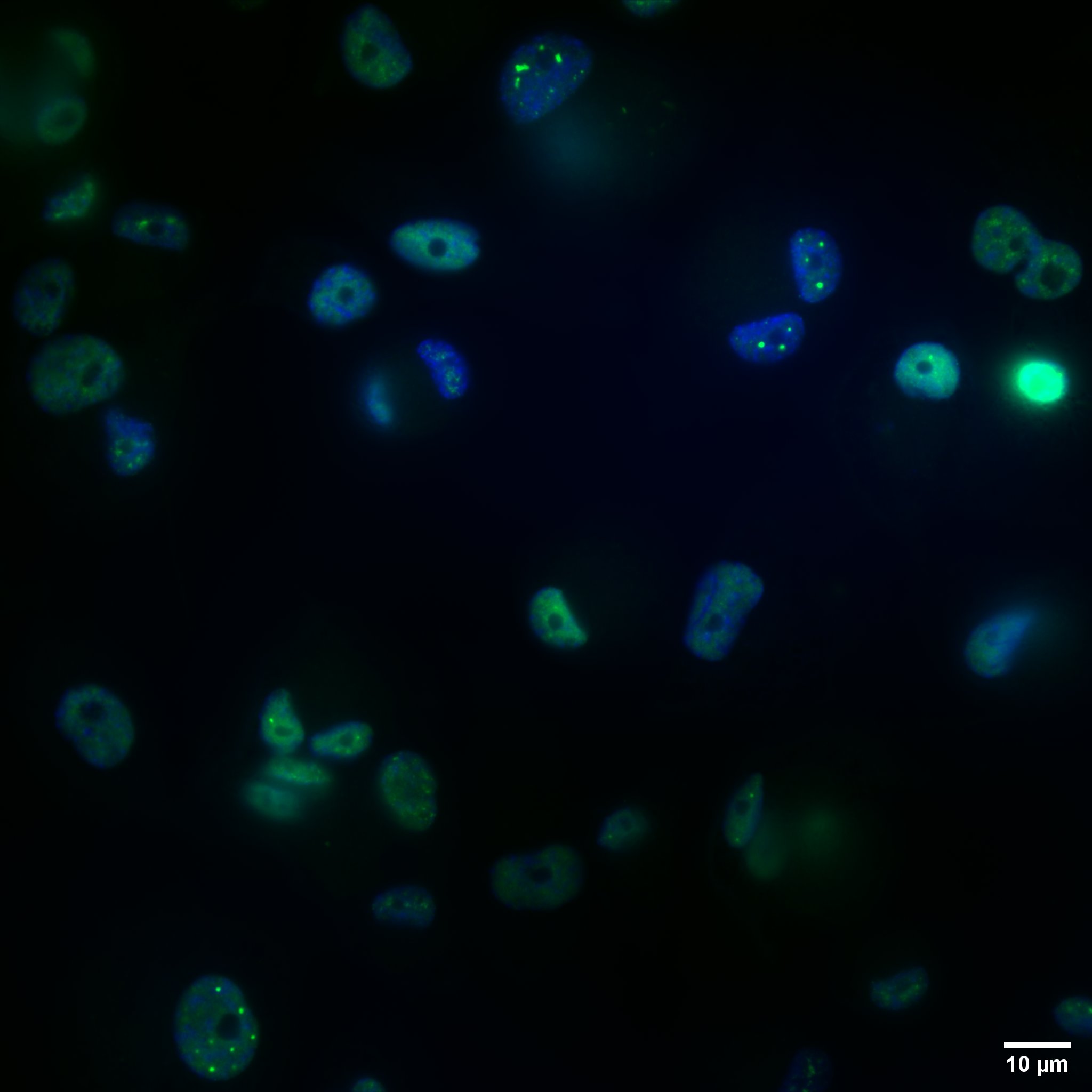

Supplement: Figure 8—source data 5. [file elife-103725-fig8-data5.zip › Figure 8-source data 5/Figure 8B-source data 2/SRRM2 -CPSF6 clusters.jpg]

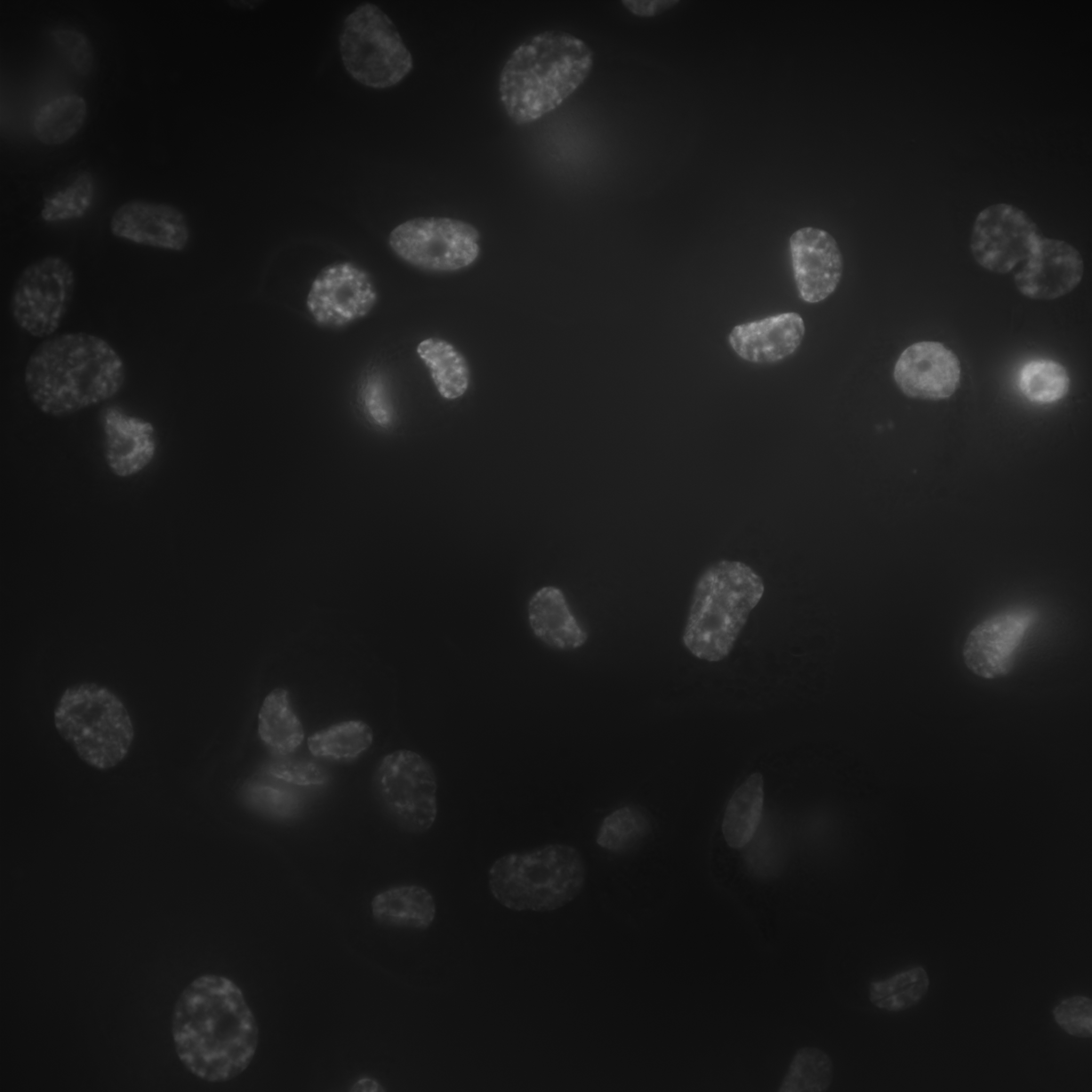

Supplement: Figure 8—source data 5. [file elife-103725-fig8-data5.zip › Figure 8-source data 5/Figure 8B-source data 2/SRRM2 -CPSF6 clusters.tif]

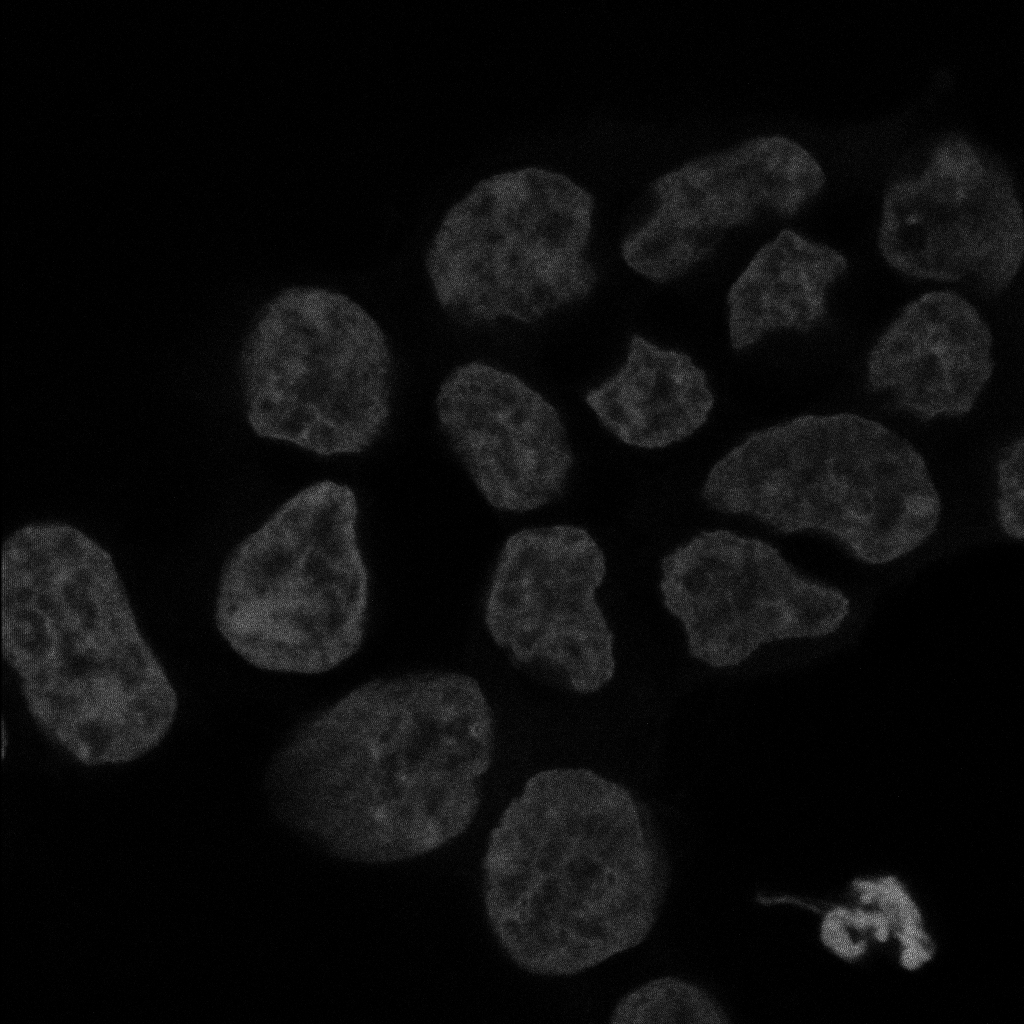

Supplement: Figure 8—source data 6. [file elife-103725-fig8-data6.zip › Figure 8-source data 6/Figure 8C-source data 2/Bottom pannel/DeltaIDRHaloTag_HaloTag and SON.tif]

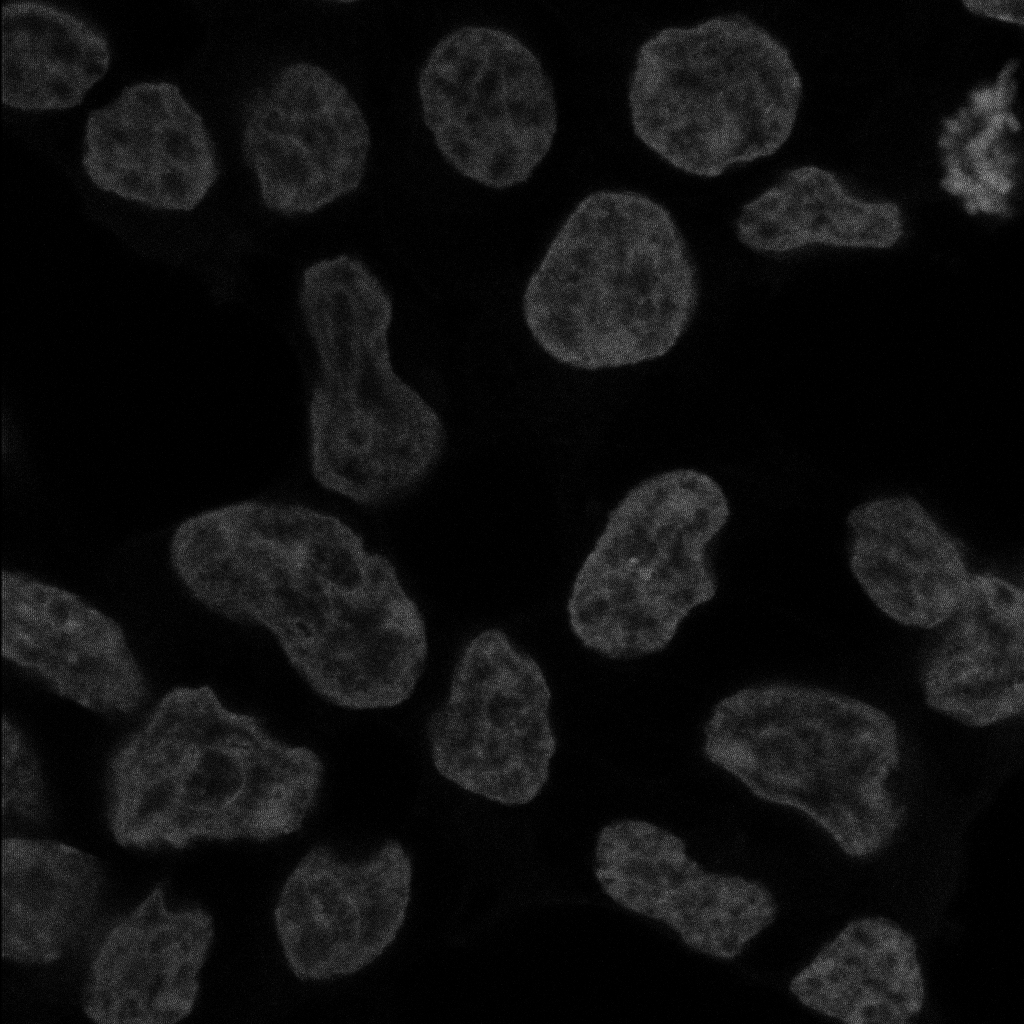

Supplement: Figure 8—source data 6. [file elife-103725-fig8-data6.zip › Figure 8-source data 6/Figure 8C-source data 2/Bottom pannel/HaloTag_HaloTag and SON.tif]

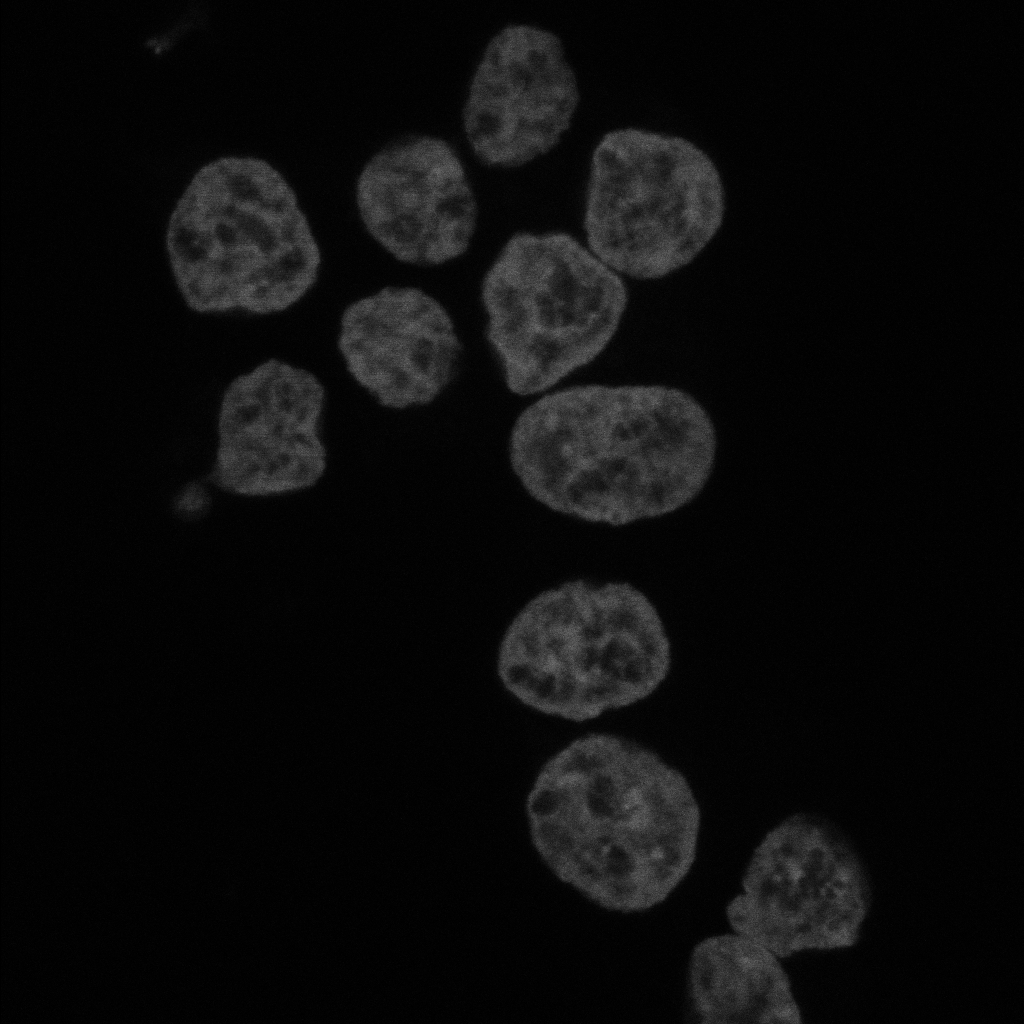

Supplement: Figure 8—source data 6. [file elife-103725-fig8-data6.zip › Figure 8-source data 6/Figure 8C-source data 2/Top pannel/DeltaIDR_HaloSRRM2.tif]

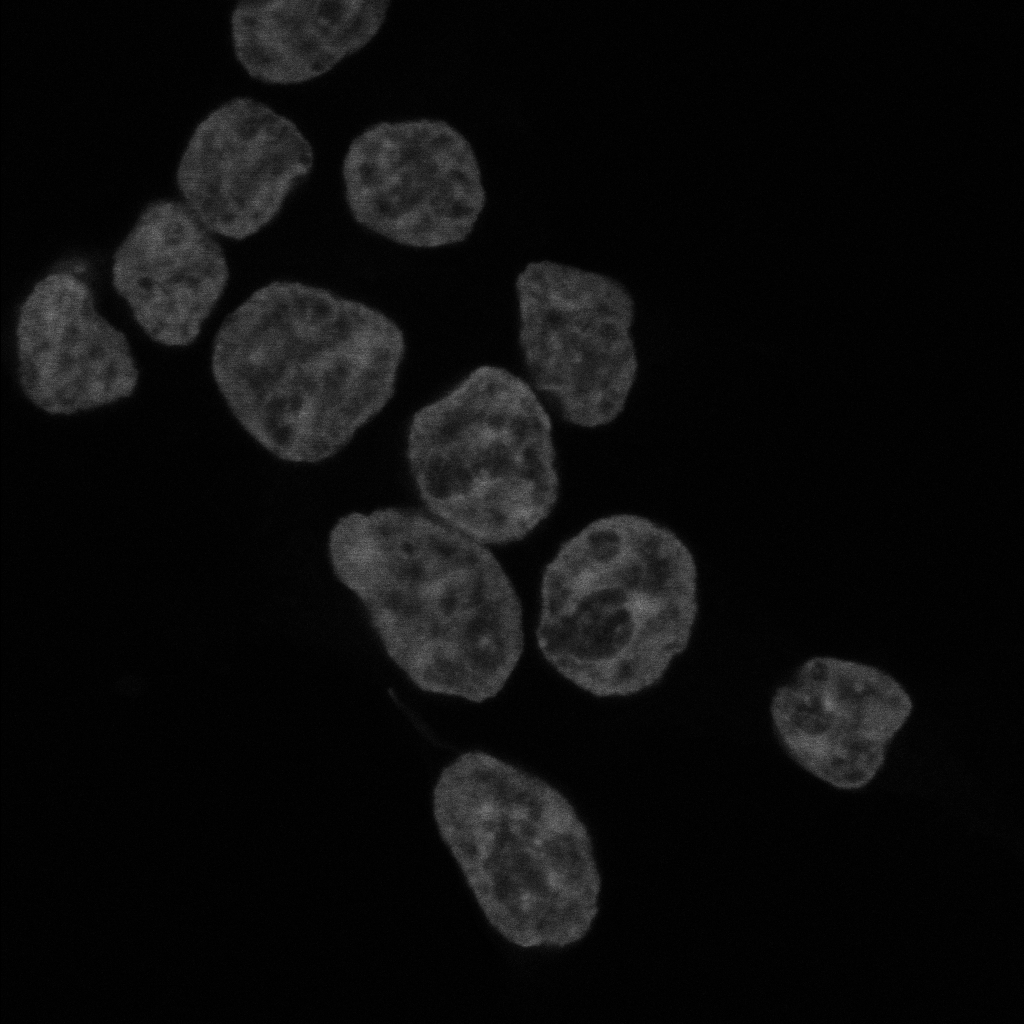

Supplement: Figure 8—source data 6. [file elife-103725-fig8-data6.zip › Figure 8-source data 6/Figure 8C-source data 2/Top pannel/Halo_HaloSRRM2.tif]
