# Supplementary material for: Decoding the biogenesis of HIV-induced CPSF6 puncta and their fusion with nuclear speckles
Source: eLife. 2026 Jan 6;13:RP103725. doi: 10.7554/eLife.103725 (PMC12774418; doi:10.7554/eLife.103725)
Supplement: Figure 3—source data 3. [file elife-103725-fig3-data3.zip › Figure 3-source data 3/Figure 3 WB annotated.pdf]

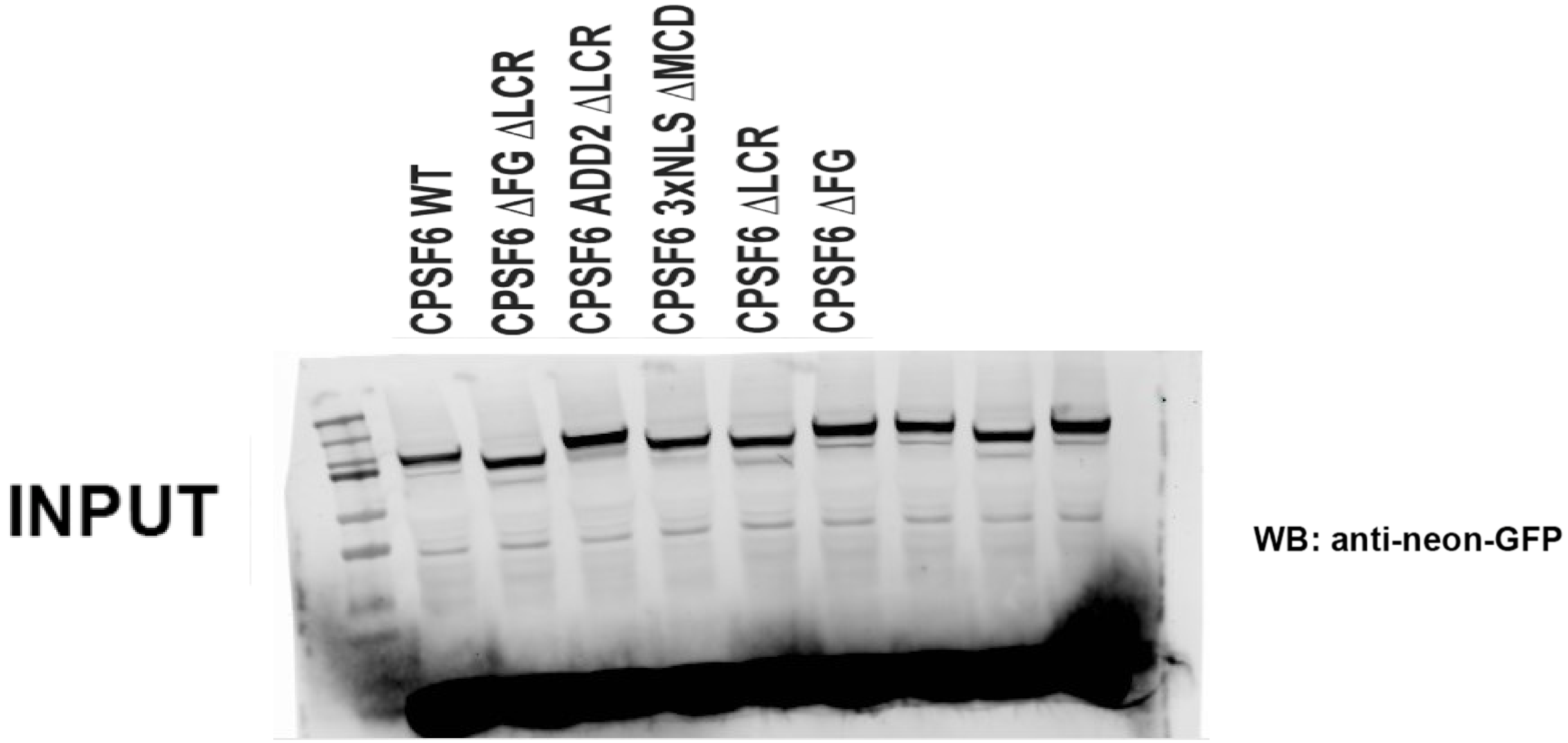
CPSF6 WT
CPSF6 ΔFG ΔLCR
CPSF6 ADD2 ΔLCR
CPSF6 3xNLS ΔMCD
CPSF6 ΔLCR
CPSF6 ΔFG
INPUT
WB: anti-neon-GFP

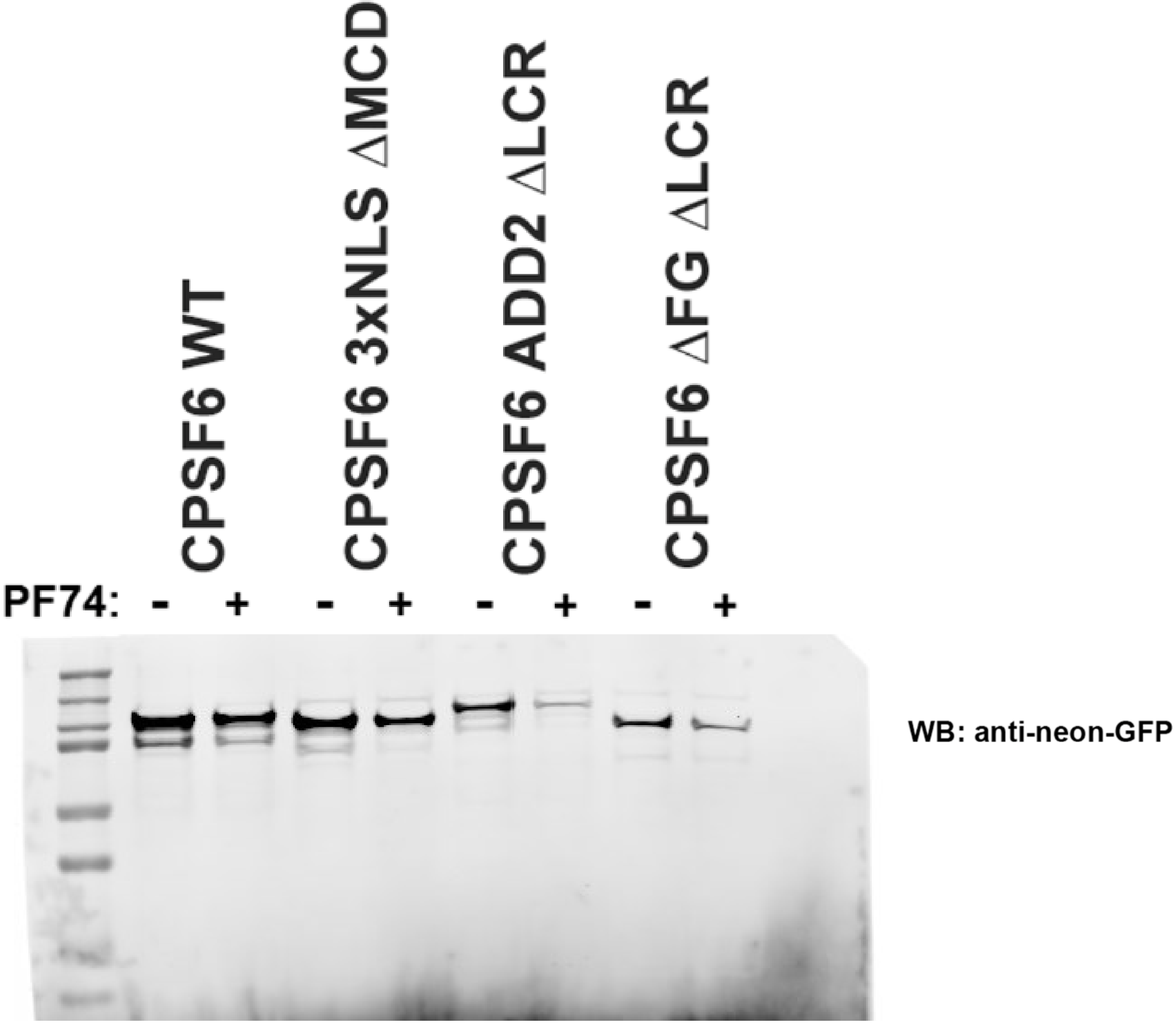
CPSF6 WT
CPSF6 3xNLS ΔMCD
CPSF6 ADD2 ΔLCR
CPSF6 ΔFG ΔLCR
PF74: - + - + - + - +
WB: anti-neon-GFP

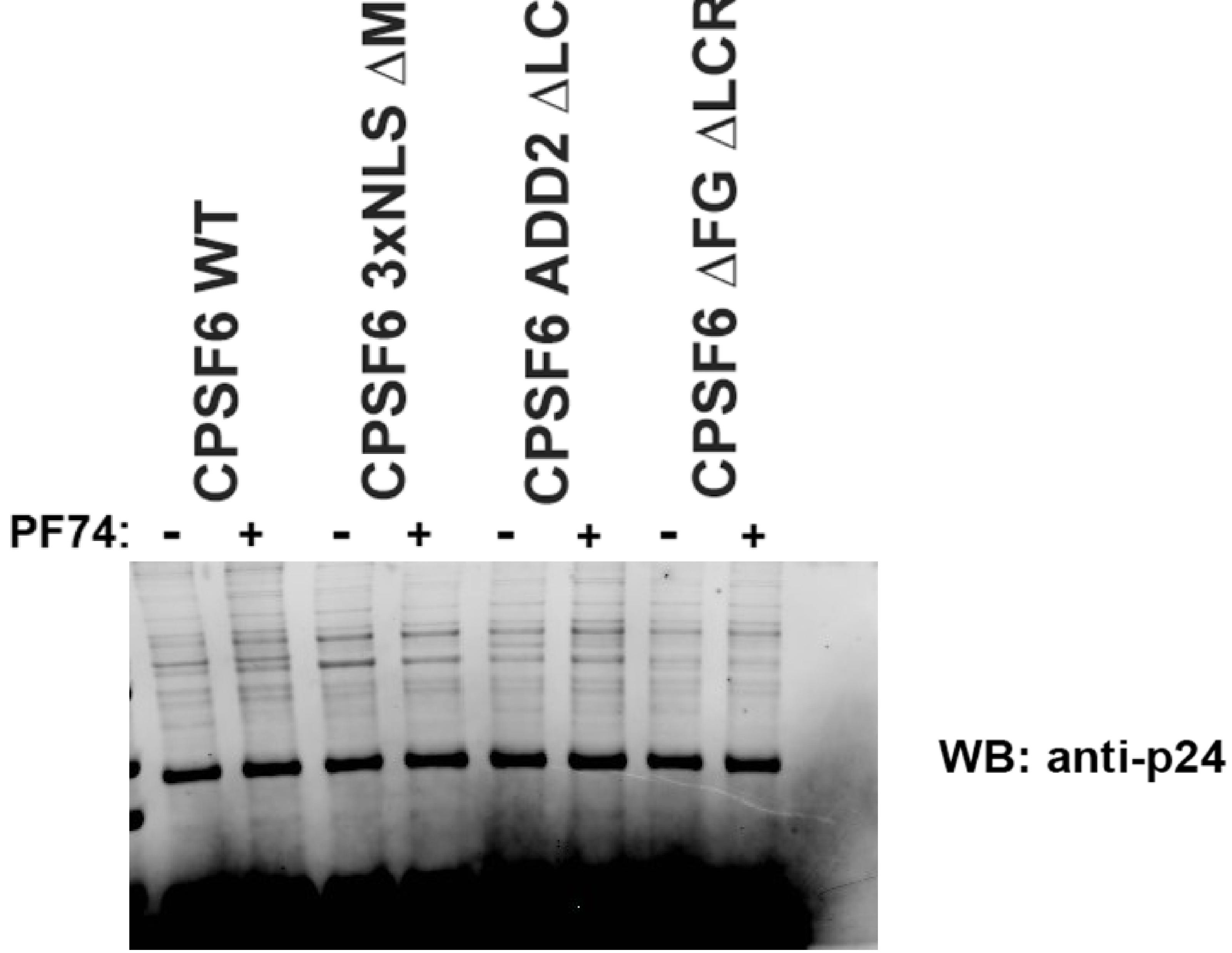

CPSF6 WT
CPSF6 3xNLS ΔM
CPSF6 ADD2 ΔLC
CPSF6 ΔFG ΔLCR
PF74: - + - + - + - +
WB: anti-p24

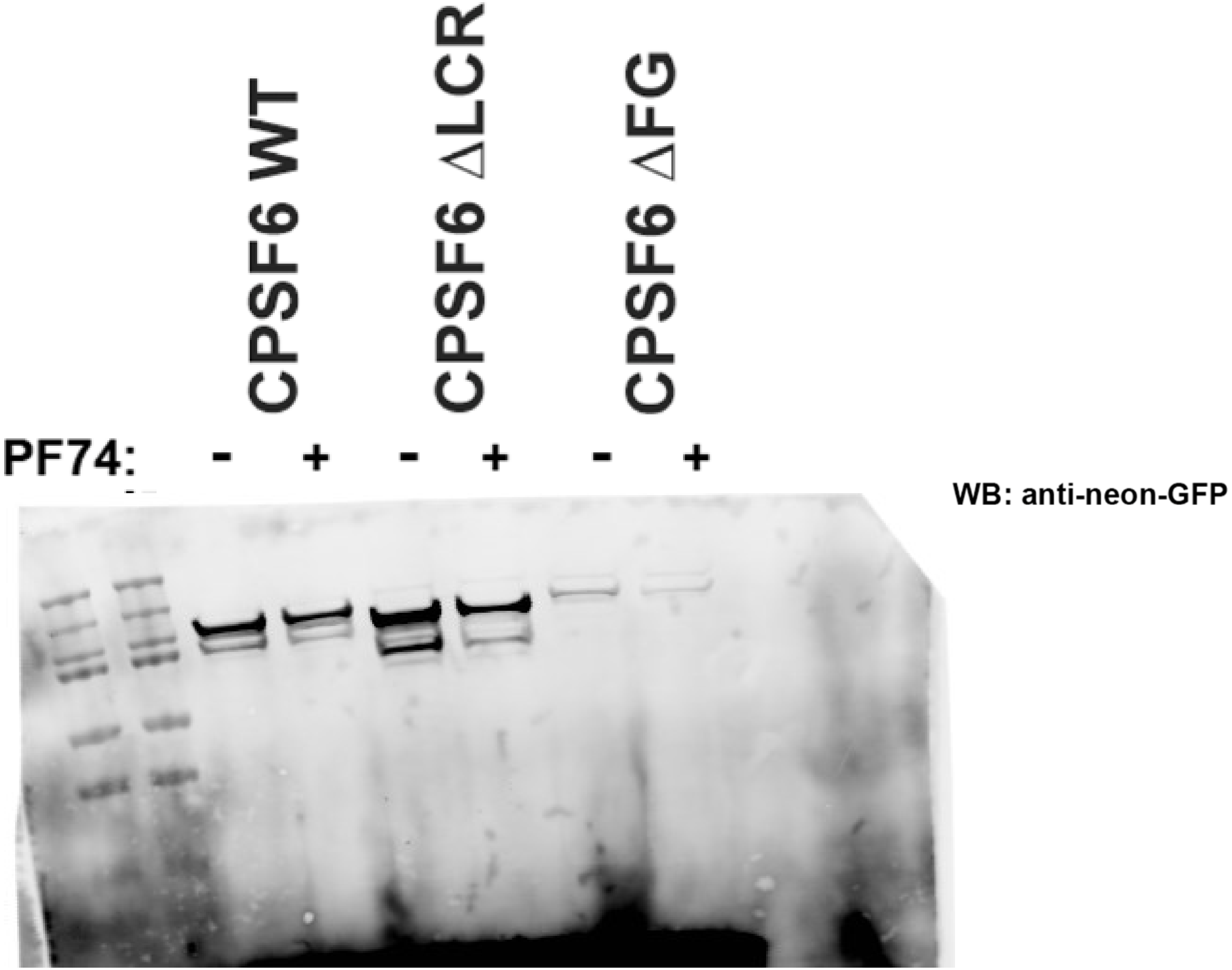
CPSF6 WT
CPSF6 ΔLCR
CPSF6 ΔFG
PF74:
-
+
-
+
-
+
WB: anti-neon-GFP

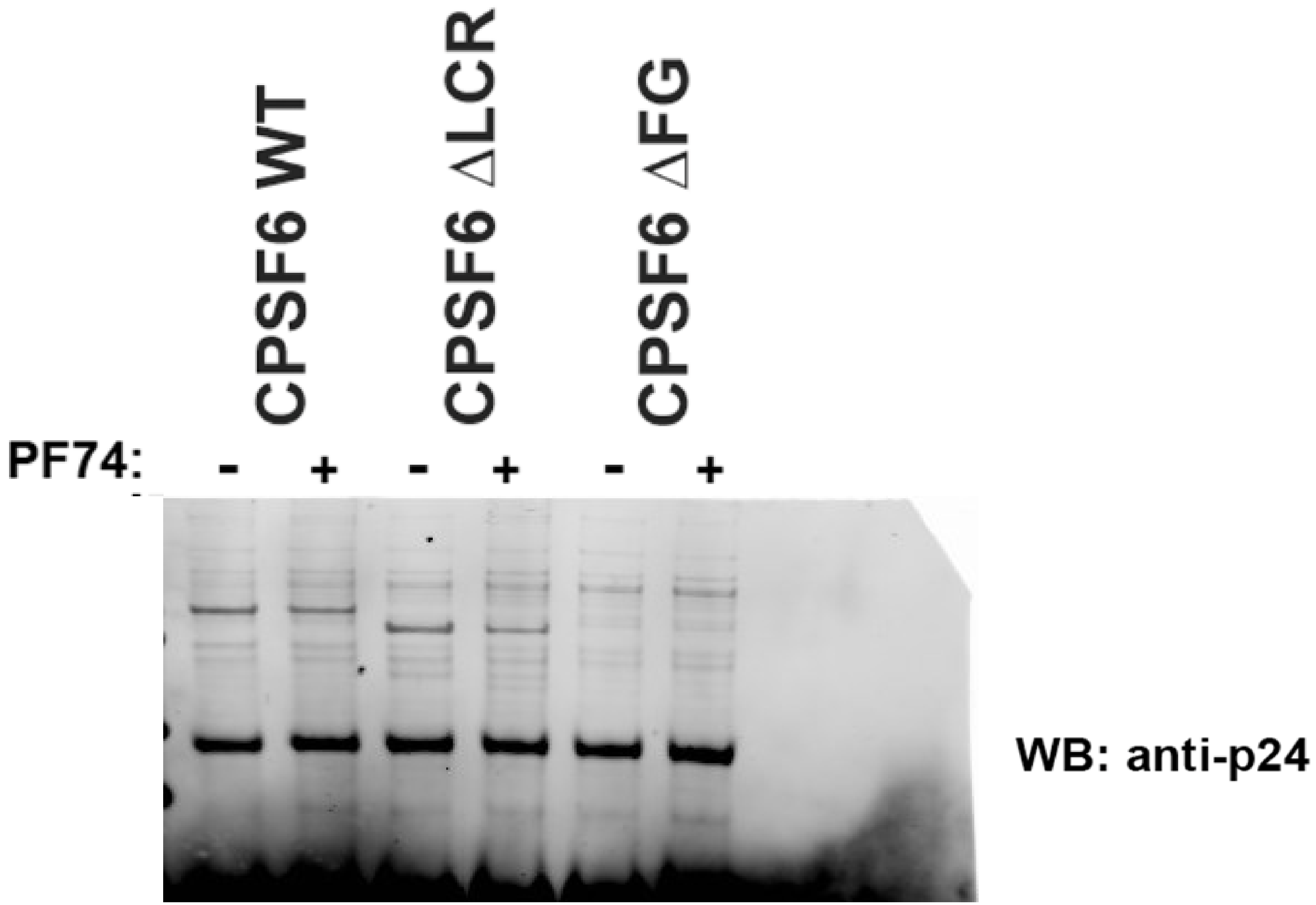
CPSF6 WT
CPSF6 ΔLCR
CPSF6 ΔFG
PF74:
- + - + - +
WB: anti-p24
