# Supplementary figures and images for: Decoding the biogenesis of HIV-induced CPSF6 puncta and their fusion with nuclear speckles (part 2 of 2)
Source: eLife. 2026 Jan 6;13:RP103725. doi: 10.7554/eLife.103725 (PMC12774418; doi:10.7554/eLife.103725)

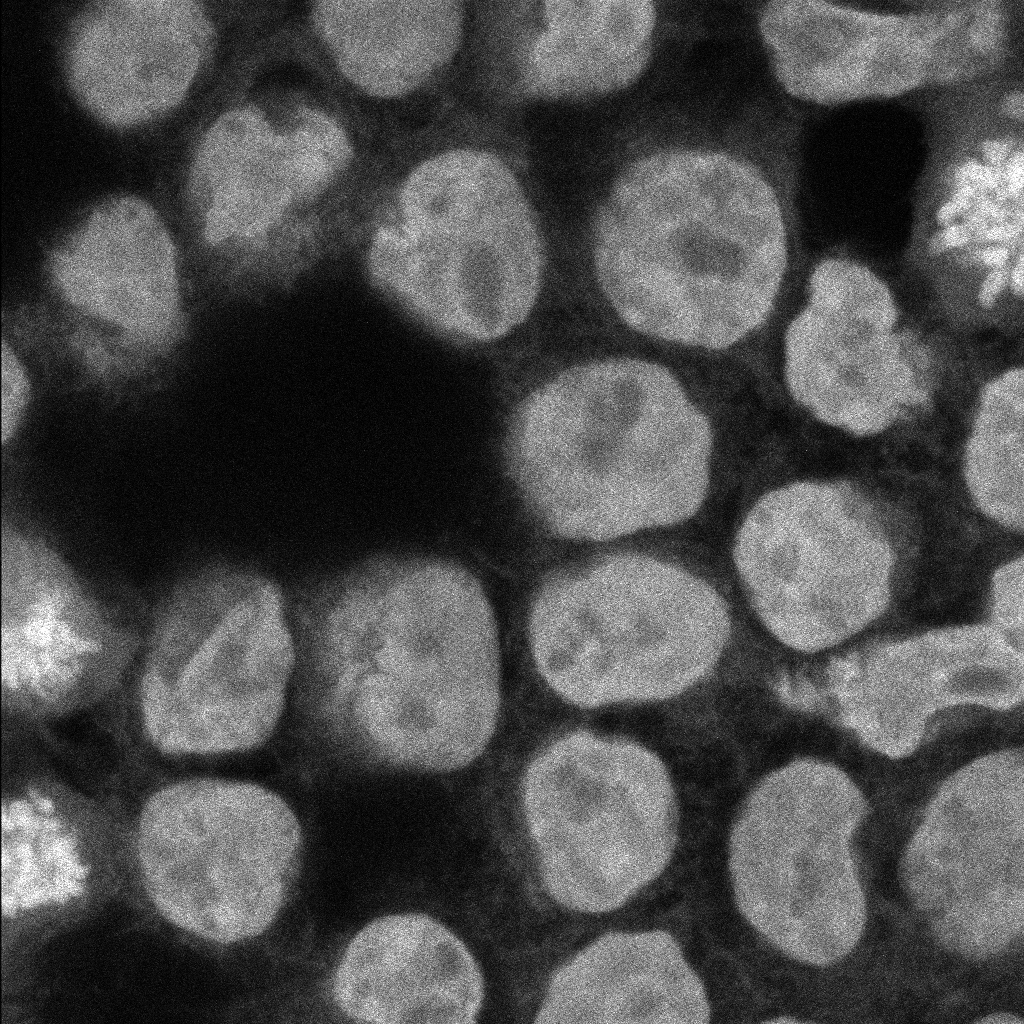

Supplement: Figure 8—source data 7. [file elife-103725-fig8-data7.zip › Figure 8-source data 7/Figure 8D-source data 2/DeltaIDR HaloTag SRRM2_Inf.tif]

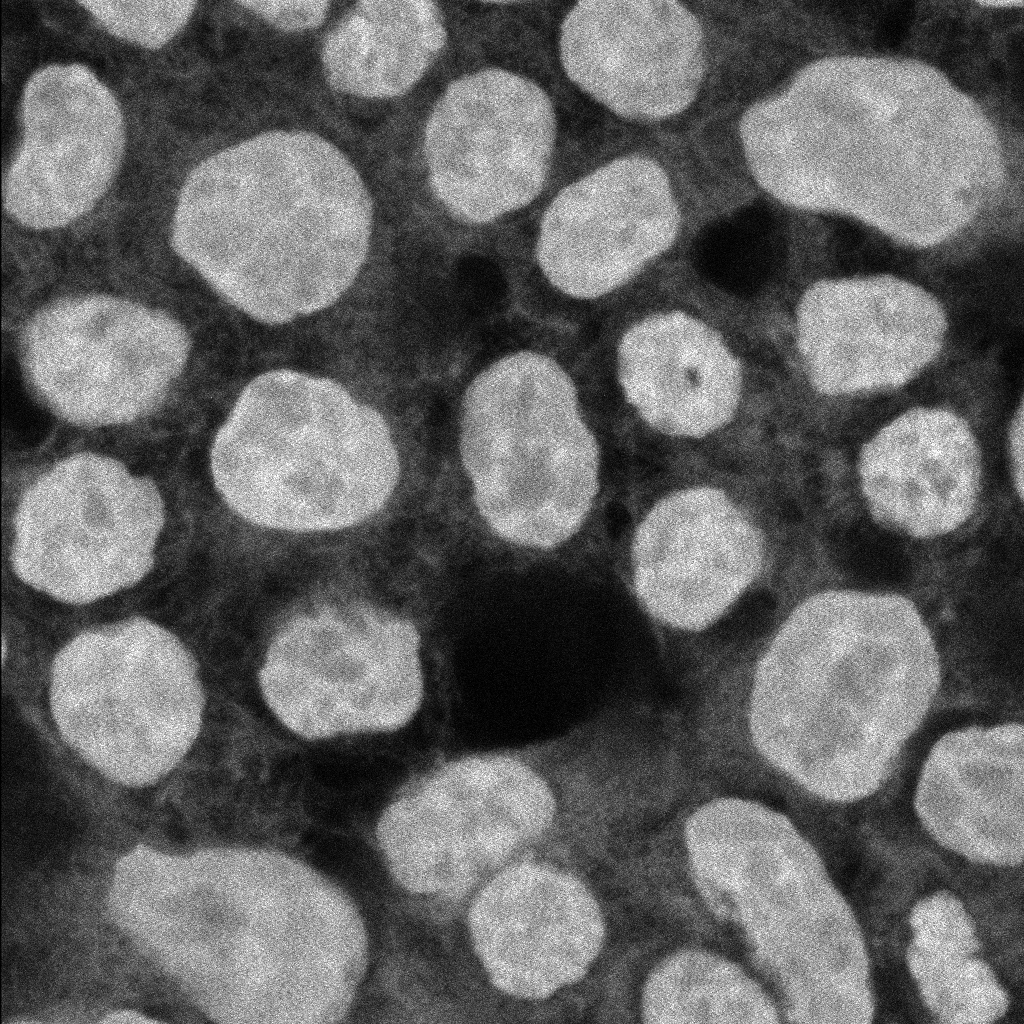

Supplement: Figure 8—source data 7. [file elife-103725-fig8-data7.zip › Figure 8-source data 7/Figure 8D-source data 2/DeltaIDR HaloTag SRRM2_NonInf.tif]

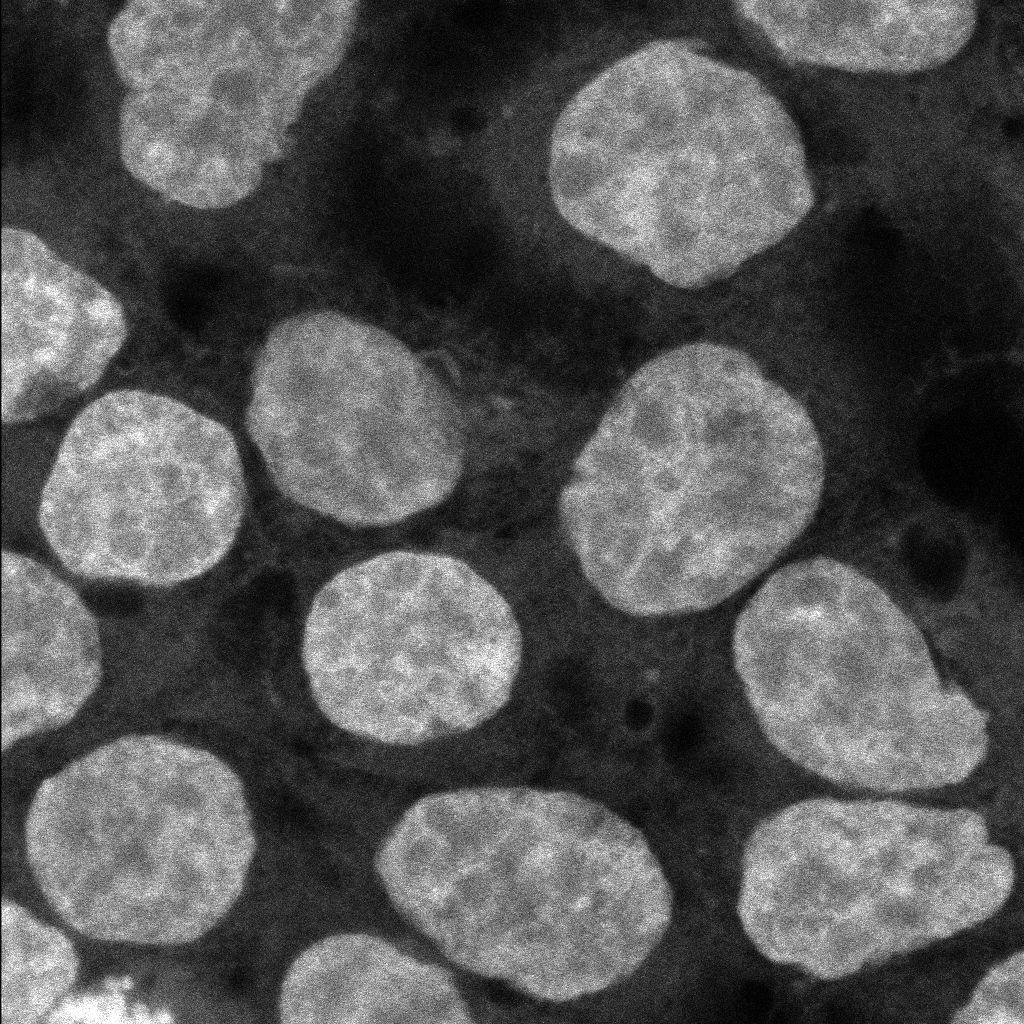

Supplement: Figure 8—source data 7. [file elife-103725-fig8-data7.zip › Figure 8-source data 7/Figure 8D-source data 2/HaloTag SRRM2_Inf.tif]

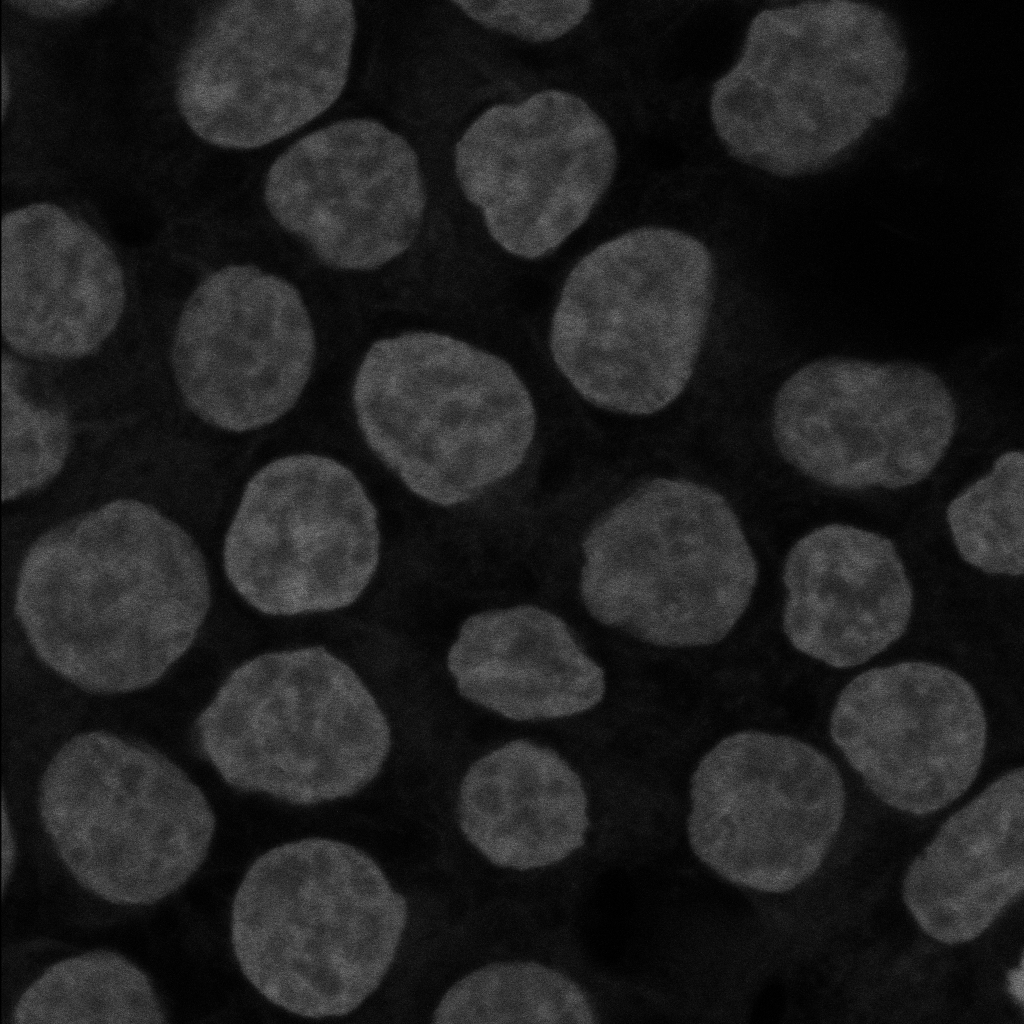

Supplement: Figure 8—source data 7. [file elife-103725-fig8-data7.zip › Figure 8-source data 7/Figure 8D-source data 2/HaloTag SRRM2_NotInf.tif]

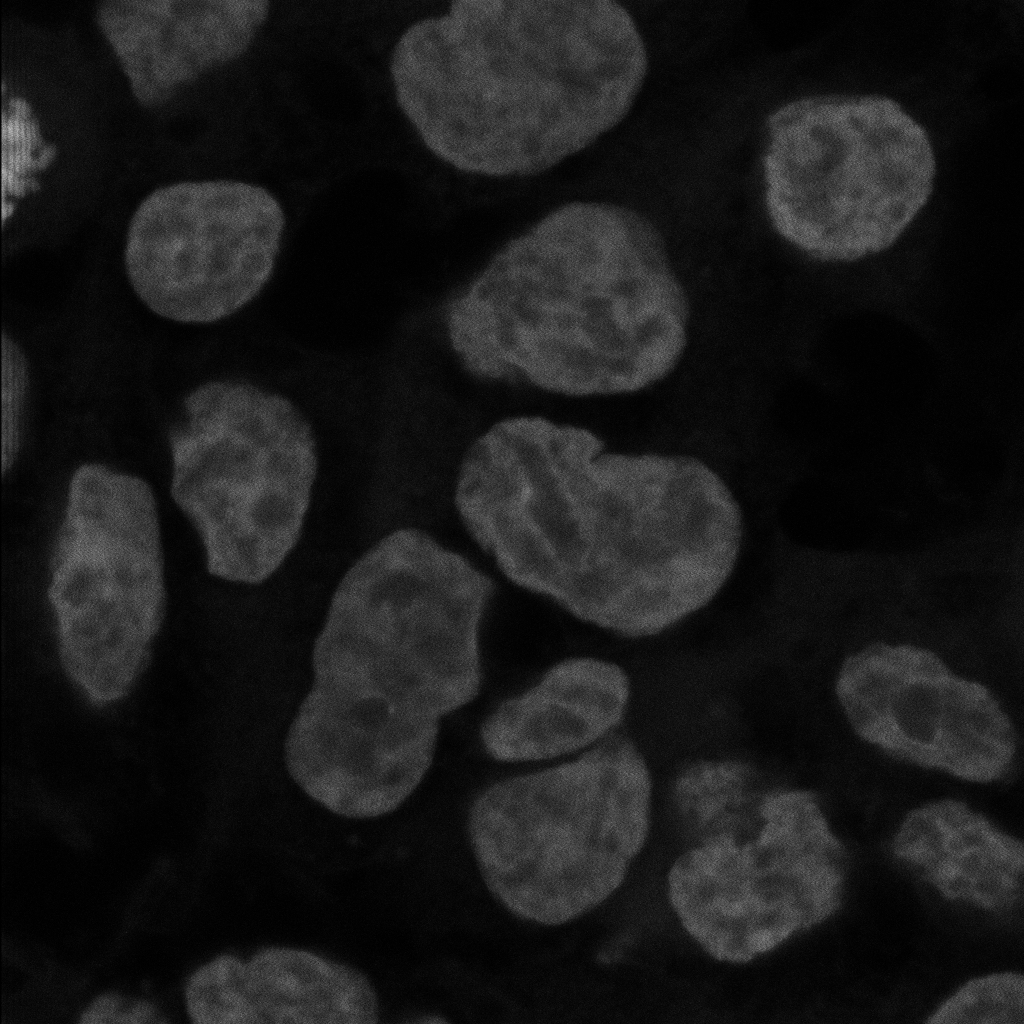

Supplement: Figure 8—source data 7. [file elife-103725-fig8-data7.zip › Figure 8-source data 7/Figure 8D-source data 2/HEK_Inf.tif]

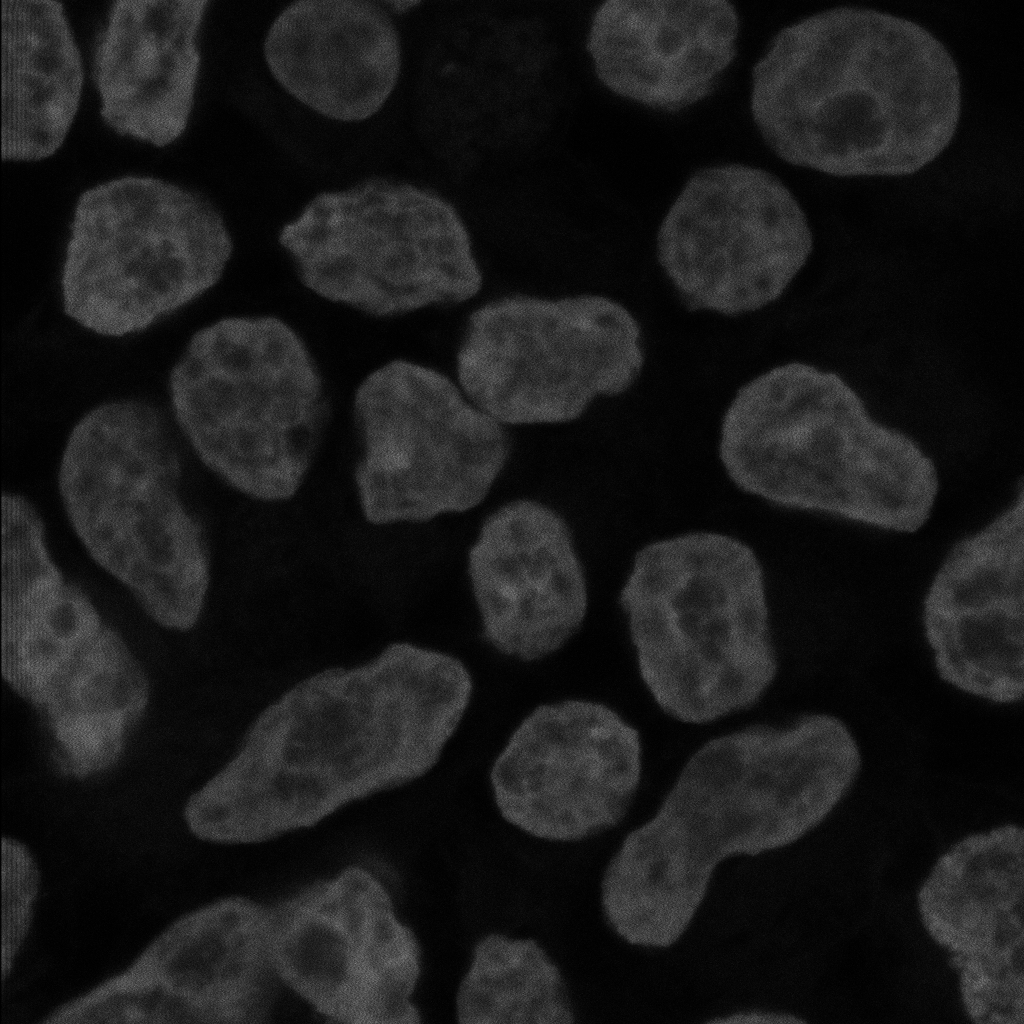

Supplement: Figure 8—source data 7. [file elife-103725-fig8-data7.zip › Figure 8-source data 7/Figure 8D-source data 2/HEK_NotInf.tif]

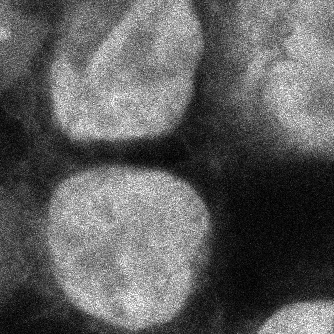

Supplement: Figure 8—source data 8. [file elife-103725-fig8-data8.zip › Figure 8-source data 8/Figure 8D-crops/DeltaIDR HaloTag SRRM2_Inf-crop and scale.tif]

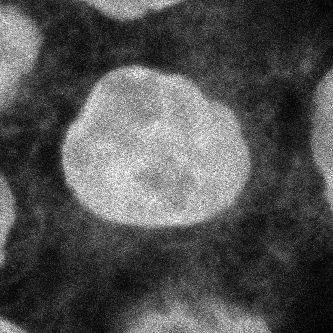

Supplement: Figure 8—source data 8. [file elife-103725-fig8-data8.zip › Figure 8-source data 8/Figure 8D-crops/DeltaIDR HaloTag SRRM2_NonInf-crop and scale.tif]

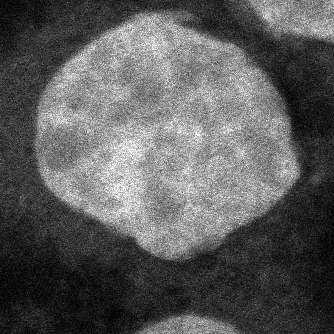

Supplement: Figure 8—source data 8. [file elife-103725-fig8-data8.zip › Figure 8-source data 8/Figure 8D-crops/HaloTag SRRM2_Inf-crop and scale.tif]

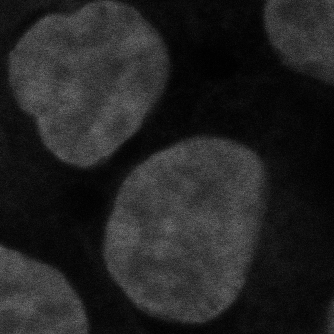

Supplement: Figure 8—source data 8. [file elife-103725-fig8-data8.zip › Figure 8-source data 8/Figure 8D-crops/HaloTag SRRM2_NotInf-crop and scale.tif]

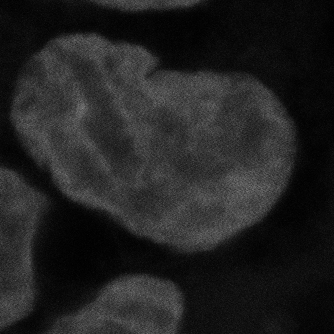

Supplement: Figure 8—source data 8. [file elife-103725-fig8-data8.zip › Figure 8-source data 8/Figure 8D-crops/HEK_Inf-crop and scale.tif]

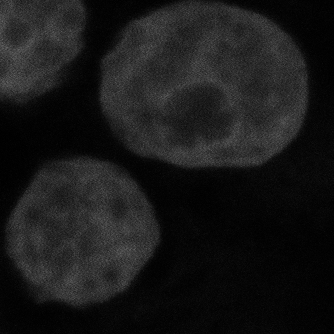

Supplement: Figure 8—source data 8. [file elife-103725-fig8-data8.zip › Figure 8-source data 8/Figure 8D-crops/HEK_NotInf-crop and scale.tif]
