# Supplementary material for: Decoding the biogenesis of HIV-induced CPSF6 puncta and their fusion with nuclear speckles
Source: eLife. 2026 Jan 6;13:RP103725. doi: 10.7554/eLife.103725 (PMC12774418; doi:10.7554/eLife.103725)
Supplement: Supplementary file 1. [file elife-103725-supp1.docx]

**Supplementary file 1**

**Deletion mutants of CPSF6 and respective plasmids.**

| **Construct** | **Modification (aa)** | **Plasmid/s used** |
| --- | --- | --- |
| pSICO CPSF6 ∆FG | Deletion, aa 313-327 | pSICO CPSF6-mNeonGreen |
| pSICO CPSF6 ∆LCR | Deletion, aa 260-312 and 328-359 | pSICO CPSF6-mNeonGreen |
| pSICO CPSF6 ∆LCR-∆FG | Deletion, aa 260-359 | pSICO CPSF6-mNeonGreen |
| pSICO CPSF6-∆LCR ADD2 | Replacement, CPSF6 -∆LCR ADD2 from pLPCX CPSF6 ADD2 at the place of CPSF6 in pSICO CPSF6-mNeonGreen | pSICO CPSF6-mNeonGreen (backbone)  pLPCX CPSF6 ADD2 (insert) |
| pSICO CPSF6-∆MCD-3xNLS | Deletion, aa 520-588; Insertion of 3xNLS at C term | pSICO CPSF6-mNeonGreen |
| pSICO CPSF6-∆MCD | Deletion, aa 520-588 | pSICO CPSF6-∆MCD NLS mNeonGreen |
| pSICO CPSF6-∆MCD PY-NLS | Deletion, aa 520-588; Insertion of PY-NLS at C term | pSICO CPSF6-∆MCD NLS mNeonGreen |
