# Supplementary material for: Decoding the biogenesis of HIV-induced CPSF6 puncta and their fusion with nuclear speckles
Source: eLife. 2026 Jan 6;13:RP103725. doi: 10.7554/eLife.103725 (PMC12774418; doi:10.7554/eLife.103725)
Supplement: Supplementary file 2. [file elife-103725-supp2.docx]

**Supplementary file 2.**

**Oligonucleotides**

| **Name** | **Sequence** |
| --- | --- |
| For CA insertion | TGGACAACCTTTTGGGCAGCCTCCATTGGGTCCCTTTCCACCTCGTCCACC |
| Rev CA insertion | CCAAAAGGTTGTCCAGGAAAAAGAACTGGCTCTATGCTGTGCCCCATATGC |
| For del CA binding | CTCCACCACCACTTCCTCCTGGCCCT |
| Rev del CA binding | GAAGTGGTGGTGGAGGGCGATCTCCT |
| For BamHI (∆LCR+ADD2) | GAACCGTCGGATCCCAGTGTGGTGGTACGGGAATTCAGGAAGATGGCGGACGGCGTGGACCACATAGACATTTACG |
| Rev BamHI (∆LCR+ADD2) | GACCGGTGGATCCCGGGCCCGCGGTACCTCACGATGACGATATTCGCGCTCTCGGTCACG |
| For del MCD + 3NLS | CAAAGTGGGCGGCAGCCCGAAAAAAAAACGCAAAGTGCCAAAAAAGAAAAGAAAAGTTGGCC |
| Rev del MCD + 3NLS | CTGCCGCCCACTTTGCGTTTTTTTTTCGGTTCCAGAATAAGAACTTTGCAACGATCATC |
| For del MCD 520-588 | AGTCCAAGCGTGAGGTACCGCGGGCC |
| Rev del MCD 520-588 | CCTCACGCTTGGACTCAATTCCATGAAGGC |
| For CPSF6 260-359 | GCATAGAGCCCTTTCCACCTCGTCCACC |
| Rev CPSF6 260-359 | GAAAGGGCTCTATGCTGTGCCCCATATGC |
| For CPSF6 w/o mNG | GTCATCGTTAAGCGGCCGCATAACTTCG |
| Rev CPSF6 w/o mNG | CCGCTTAACGATGACGATATTCGCGCTCTC |
| For CPSF6 del MCD w/o mNG | AAGTTGGCTAAGCGGCCGCATAACTTCG |
| Rev CPSF6 del MCD w/o mNG | CCGCTTAGCCAACTTTTCTTTTCTTTTTTGGC |
| For BamHI CPSF6 | GACCGGTGGATCCCGGGCCCGCGGTACCTCACGATGACGATATTCGCGCTCTCGGTCACGC |
| Rev BamHI CPSF6 | GAACCGTCGGATCCCAGTGTGGTGGTACGGGAATTCAGGAAGATGGCGGACGGCGTGGACCACATAGACATTTACG |
| For del MCD w/o NLS w/o mNG | AGTCCAAGTAAGCGGCCGCATAACTTCGT |
| Rev del MCD w/o NLS w/o mNG | CCGCTTACTTGGACTCAATTCCATGAAGGCA |
| For del MCD w/o mNG with PY NLS | GCGAGCACAGACAGGAGAGAAGAGACAGACCCTACTAAGCGGCCGCATAACTTCGT |
| Rev del MCD w/o mNG with PY NLS | CCTGTCTGTGCTCGCCCTTGTCCATCTTGCCGGGCTTGGACTCAATTCCATGAAGGCA |
| Rev seq CPSF6 mut | CATGATCTCGTGAAGCGAGC |
| For seq CPSF6ADD2 | AAATGTCGTAACAACTCCGC |
| Rev seq CPSF6ADD2 | CTTTGCTACCCAATGGAGG |
| U5R titer For | GGCTAACTAGGGAACCCACTG |
| U5R titer Rev | GCTAGAGATTTTCCACACTGACTAA |
| CD3 titer For | GGCTATCATTCTTCTTCAAGGTA |
| CD3 titer Rev | CCTCTCTTCAGCCATTTAAGTA |
| SRRM2-ASO | ATCTACTACGAGACCTGCGTC |
| SON-ASO | ACATCTGAGCATCCATGGTGC |
| SCR-ASO | TGACCCTATGCTGTTCCTATA |
